# Role of non-coding RNAs in maintaining primary airway smooth muscle cells

**DOI:** 10.1186/1465-9921-15-58

**Published:** 2014-05-16

**Authors:** Mark M Perry, Eleni Tsitsiou, Philip J Austin, Mark A Lindsay, David S Gibeon, Ian M Adcock, Kian Fan Chung

**Affiliations:** 1 Experimental Studies, National Heart and Lung Institute, Imperial College, London & Royal Brompton NIHR Biomedical Research Unit, Dovehouse Street, London SW3 6LY, UK; 2 Respiratory Research Group, University Hospital of South Manchester, University of Manchester, Southmoor Road, Manchester M23 9LY, UK; 3 Department of Pharmacy and Pharmacology, University of Bath, Claverton Down, Bath BA2 7AY, UK

**Keywords:** Lung disease, Dexamethasone, Transcriptome, Long noncoding RNA, microRNA

## Abstract

**Background:**

The airway smooth muscle (ASM) cell maintains its own proliferative rate and contributes to the inflammatory response in the airways, effects that are inhibited by corticosteroids, used in the treatment of airways diseases.

**Objective:**

We determined the differential expression of mRNAs, microRNAs (miRNAs) and long noncoding RNA species (lncRNAs) in primary ASM cells following treatment with a corticosteroid, dexamethasone, and fetal calf serum (FCS).

**Methods:**

mRNA, miRNA and lncRNA expression was measured by microarray and quantitative real-time PCR.

**Results:**

A small number of miRNAs (including miR-150, −371-5p, −718, −940, −1181, −1207-5p, −1915, and −3663-3p) were decreased following exposure to dexamethasone and FCS. The mRNA targets of these miRNAs were increased in expression. The changes in mRNA expression were associated with regulation of ASM actin cytoskeleton. We also observed changes in expression of lncRNAs, including natural antisense, pseudogenes, intronic lncRNAs, and intergenic lncRNAs following dexamethasone and FCS. We confirmed the change in expression of three of these, LINC00882, LINC00883, PVT1, and its transcriptional activator, c-MYC. We propose that four of these lincRNAs (RP11-46A10.4, LINC00883, BCYRN1, and LINC00882) act as miRNA ‘*sponges*’ for 4 miRNAs (miR-150, −371-5p, −940, −1207-5p).

**Conclusion:**

This *in-vitro* model of primary ASM cell phenotype was associated with the regulation of several ncRNAs. Their identification allows for *in-vitro* functional experimentation to establish causality with the primary ASM phenotype, and in airway diseases such as asthma and chronic obstructive pulmonary disease (COPD).

## Introduction

Airway smooth muscle cells (ASMCs) are an integral part of the airways (from large to small) and contribute to the airflow obstruction in chronic airway respiratory diseases such as asthma and chronic obstructive pulmonary disease (COPD) through its contractile properties that lead to airway narrowing. In addition, ASM cells are in a state of increased proliferation due to the effect of growth factors that lead to an increase in ASM mass in these diseases [1-4]. Furthermore, they are pro-inflammatory, releasing many cytokines and chemokines [5,6]. The regulation of the contractile and proliferative responses of ASM in disease remains unclear. In order to understand this, we have examined the potential contribution of noncoding RNA in these processes in ASM cells.

We have recently found that the proliferation and increased release of IL-6 found in ASM cells from patients with asthma was under the negative regulation of cyclin inhibitors, p21^WAF1^ & p27^kip1^, which were controlled by the microRNA; mir-221 [7]. We hypothesized that other miRNAs may also be involved in other aspects of ASM function. In addition to the miRNA family of short noncoding RNAs that consist of less than 200 nucleotides, there is now accumulating evidence that long noncoding RNAs (lncRNAs with more than 200 nucleotides) can regulate multiple biological responses and that changes in their expression may be related to the development of disease [8,9].

There are a surprisingly few studies demonstrating the importance of miRNAs in ASM function (Extensively reviewed in [10]) and these only validate the importance of a handful of miRNAs; let-7a, miR-16, miR-25, miR-26a, miR-133, miR-143, miR-145, miR-146a and miR-206. Furthermore, there are currently, no studies examining the roles of lncRNAs in ASM. Therefore, we have adopted a transcriptomic approach to investigate the differential expression of mRNAs, miRNAs and lncRNAs in primary ASM cells and exposed them to a known inducer of proliferation, fetal calf serum (FCS), and also examined the effect of the corticosteroid, dexamethasone.

## Methods

### Patient recruitment

Healthy subjects with no previous history of disease were recruited. Each subject underwent a fiberoptic bronchoscopic study under sedation with midazolam and topical anaesthesia to the airways with lidocaine. Airway biopsies were taken from segmental and sub-segmental airways of the right lower lobe. This study was approved by the Royal Brompton & Harefield NHS Trust Ethics committee and all subjects gave written informed consent. Subject data are shown in Table 1.

**Table 1:** Subject data

*n*	9
Age (yrs.)	36.4 ± 12.7
Sex (♂ / ♀)	7 / 2
Smoking history	0
Atopy (*n*)*	0
Receiving medications (*n*)	0
FEV_1_ (L)	4.02 ± 0.48
FEV_1_ (% Predicted)	104.23 ± 7.28
FEV_1_/FVC (%)	78.89 ± 5.98
PC_20_ (mg/ml)	> 16

FEV_1_, forced expiratory volume in 1 s; FVC, forced vital capacity; PC_20_, provocative concentration of methacholine causing a 20% fall in FEV_1_; N/A: not available. *Defined as positive skin prick tests to one or more common aeroallergens. Data shown as mean ± SEM.

### ASM cell culture and stimulation

ASM cells were picked from bronchial biopsies and cultured in Dulbecco’s Modified Eagles Medium (DMEM) supplemented with 4 mM L-glutamine, 20 U/l of penicillin, 20 μg/ml streptomycin, and 2.5 μg/ml amphotericin B and 10% foetal calf serum (FCS), as previously described [7,11]. At confluence, ASM cell cultures exhibited a typical “hill-and-valley” appearance. Immunofluorescence techniques for calponin, smooth muscle α-actin and myosin heavy chain revealed that > 95% of the cells displayed the characteristics of smooth muscle cells in culture. Cells at passages 3–4 were used for all experiments, from 9 different donors.

Confluent cells were growth-arrested by FCS deprivation for 24 h in DMEM supplemented with sodium pyruvate (1 mM), L-glutamine (2 mM), nonessential amino acids (1:100), penicillin (100 U/ml)/streptomycin (100 μg/ml), amphotericin B (1.5 μg/ml), and bovine serum albumin (0.1%). Cells were pre-treated with dexamethasone (10^−7^ M) for 1 h, before being stimulated with 2.5% FCS for 24 h. Supernatants were removed and IL-6 and CXCL8 levels determined by DuoSet ELISA (R&D Systems). Cell proliferation was measured by the Cell Proliferation ELISA BrdU kit (Roche Applied Science), an assay comparable to cell counting as confirmed by flow cytometry [7,11]. Cellular viability was assessed by MTT assay [12]. Each patient sample was replicated over 3 wells.

### RNA extraction

Total RNA was extracted using the *mir*Vana™ miRNA isolation kit (Ambion Europe). RNA was eluted in 50 μl RNase-free water (Promega UK, Southampton, UK) and stored at −80°C. RNA content and purity was measured using a BioTek PowerWave XS (SSi Robotics, Tustin, CA, U.S.A.) spectrophotometer.

### Microarray analysis

LncRNA and mRNA expression was determined using the Agilent SurePrint G3 Human GE microarrays and miRNA expression was determined using Agilent Human miRNA microarray following the manufacturer’s instructions.

Total RNA samples (50 ng) used in lncRNA and mRNA microarrays were initially labeled with Spike-In control A (for Cyanine 3-CTP) or B (for Cyanine 5-CTP). Labelled samples were then used for cDNA synthesis using the cDNA Master Mix (Agilent) and were incubated for 2 h at 40°C followed by 15 min at 70°C to inactivate the AffinityScript enzyme. The synthesized cDNA was then used for cRNA synthesis and amplification using the Transcription Master Mix with either Cyanine 3 or Cyanine 5 and incubated at 40°C for 2 h.

Labeled and amplified RNA was then purified using the RNeasy Mini Kit (Qiagen) and quantified using the NanoDrop Spectrophotometer’s Microarray Measurement function. The Cyanine 3 or Cyanine 5 concentrations and the cRNA concentration were used to calculate the yield (μg) and the specific activity (pmol Cy3 or Cy5 per μg cRNA) of each sample. For each microarray reaction, 300 ng of Cyanine 3- and 300 ng of Cyanine 5-labelled, linearly amplified cRNA samples were mixed and incubated together with Fragmentation buffer for 30 min, followed by the addition of Hybridization buffer. Samples were loaded onto SurePrint G3 (8 × 60 K) microarray slides (Agilent) and hybridized at 65°C for 17 h at 10 rpm using Agilent’s Hybridization oven and SureHyb chamber. The microarrays were then disassembled and washed in GE Wash Buffer 1 (Agilent) for 60 sec at RT, followed by GE Wash Buffer 2 for 60 sec at 37°C, followed by an acetonitrile wash (10 sec at RT) and the final wash in Stabilization and Drying solution for 30 sec at RT, to improve microarray results by preventing ozone-mediated fluorescent signal degradation. The microarrays were scanned with the Agilent Microarray Scanner G2565BA using the profile for 2-colour microarrays (AgilentG3_GX_2Color) at 5 μm resolution, dyes channel Red&Green, Scan Area 61 × 21.6 mm.

miRNA-enriched RNA samples (100 ng) used in the microRNA microarrays were labeled with the Labeling Spike-In solution using Agilent’s miRNA Complete Labeling and Hyb Kit and then the samples were dephosphorylated by incubation at 37°C on a heat block for 30 min. DMSO was added to the dephosphorylated RNA for denaturation for 10 min, followed by the assembly of the Labeling Reaction, where samples were incubated with Cyanine 3-pCp and allowed to ligate for 2 h at 16°C. The labeled RNA was purified using Micro Bio-Spin 6 columns (Bio-Rad) and the desalted samples were dried up in a vacuum concentrator at 50°C for 1 h to remove DMSO. Samples were resuspended in Hybridization Mix using Hyb Spike-In solution and Hybridization Buffer and were loaded onto Agilent Human miRNA microarrays and hybridized at 55°C for 20 h at 20 rpm using Agilent’s Hybridization oven and SureHyb chamber. The microarrays were then disassembled and washed in GE Wash Buffer 1 (Agilent) for 60 sec at RT, followed by GE Wash Buffer 2 for 60 sec at 37°C. The microarrays were scanned with the Agilent Microarray Scanner G2565BA using the profile for miRNA microarrays (AgilentHD_miRNA) at 2 μm resolution, dye channel Green, Scan Area 61 × 21.6 mm.

Following normalization against internal controls provided within the labeling kits, probes which had background expression (signal value of mRNA < 4.5 and miRNA < 5.0) were removed. The threshold of background expression was determined using samples that processed but which contained no RNA. Since initial analysis of mRNA, lncRNA and miRNA between baseline and FCS-treated cells or dexamethasone + FCS-treated cells showed none that gave a FDR < 0.1, differential expression (p value) was determined by 3-way ANOVA using the Partek Genomics Suite. We report changes in expression with p < 0.05. Predicted interactions between miRNAs and mRNA were determined using TargetScan 5.2 within the Partek Genomics Suite. The changes in profile of mRNA expression were analyzed using the bioinformatics database, DAVID 6.7 [13,14] and possible miRNA–lncRNA interactions, were examined using LNCipedia 2.0 [15]. Microarray data has been deposited at GEO.

### Quantitative PCR measurement of miRNA and mRNA expression

miRNA and lncRNA expression was undertaken with the 2-step Applied Biosystems TaqMan RT-PCR protocol (Applied Biosystems) and normalized to 18S, as previously described [7,11,16]. mRNA expression was determined using TaqMan RT-PCR with Assays on Demand (Applied Biosystems). The separate-well 2^-(ΔΔCt)^ method was used to determine relative quantitative levels of individual miRNAs, lincRNAs and mRNAs.

### Data and statistical analysis

The RT-PCR results are presented as the mean ± SEM of at least nine independent experiments. Statistical analysis was performed by Mann–Whitney U-test test, assuming non-parametric distribution. P values of < 0.05 were considered significant.

## Results

### Effect of Dexamethasone and FCS upon ASM cell proliferation, IL-6 and CXCL8 release

We have previously demonstrated that 2.5% v/v FCS and 10^−7^ M dexamethasone are optimum concentrations to induce and, conversely, inhibit both a pro-inflammatory and proliferative state in primary ASM [7,11,17].

**Figure 1:**
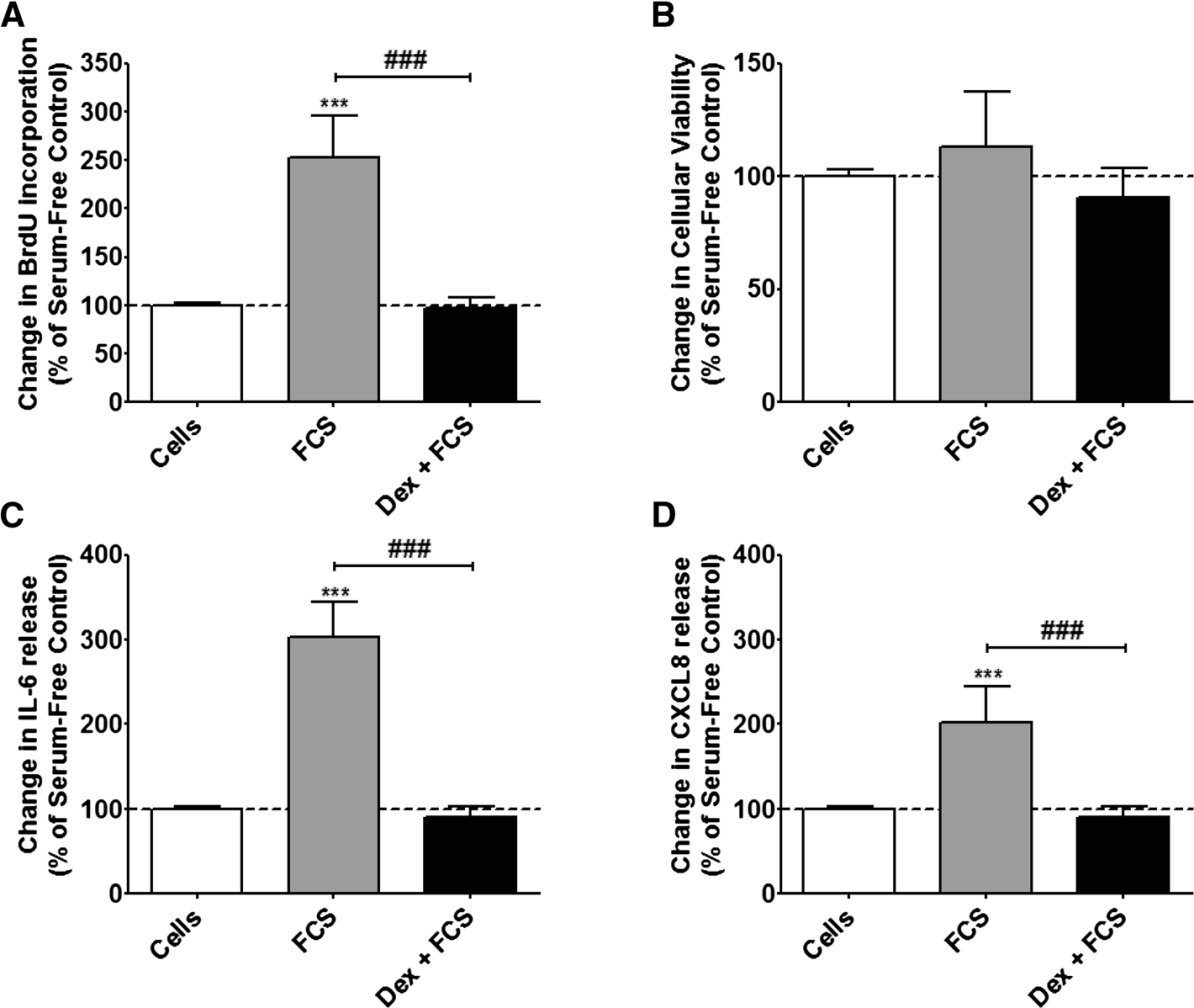
**Effect of dexamethasone and FCS upon primary ASM cell proliferation, IL-6 and CXCL8 release.** ASM cells were incubated with dexamethasone (10^−7^ M) for 1 h before being stimulated with FCS (2.5%) for 24 h. DNA synthesis **(A)**, cell viability **(B)**, IL-6 **(C)** and CXCL8 **(D)** release were measured by BrdU ELISA, DuoSet ELISA or MTT assay, respectively. Bars represent mean ± SEM from 9 primary ASM cells. ^***/###^p < 0.001.

To confirm activation, in this study, of primary ASM by the stimulus (FCS), and pre-treatment with corticosteroid (dexamethasone), we measured DNA synthesis, IL-6, and CXCL8 release by ELISA. At 24 h, FCS (2.5%) induced DNA synthesis by ~2.5-fold (p < 0.001), IL-6 release ~3-fold (p < 0.001) and CXCL8 release ~2-fold (p < 0.001) (Figure 1A, C & D). When ASM cells were exposed to dexamethasone (10^−7^ M) for 1 h prior to stimulation with FCS, this increase was completely attenuated (p < 0.001) (Figure 1A, C & D). No effect was observed upon cellular viability (Figure 1B).

### Effect of dexamethasone and FCS upon mRNA expression

**Figure 2:**
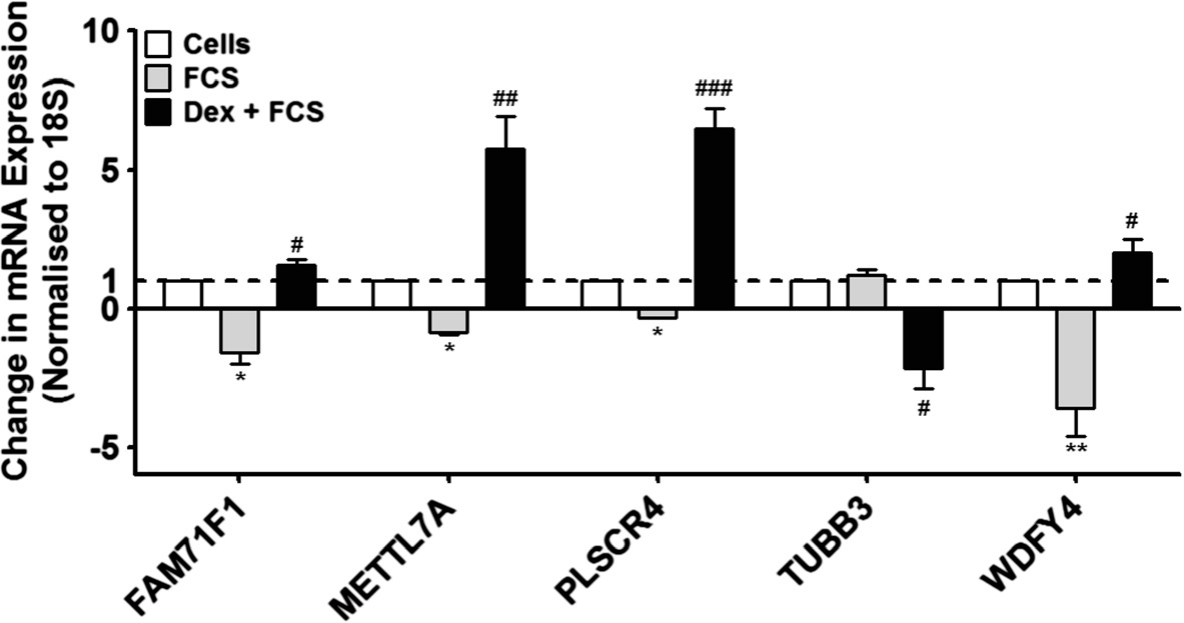
**Effect of dexamethasone and FCS upon mRNA expression in primary ASM cells.** ASM cells were incubated with dexamethasone (10^−7^ M) for 1 h before being stimulated with FCS (2.5%) for 24 h. To validate the array data, the expression of 5 mRNAs were confirmed by TaqMan RT-PCR. Bars represent mean ± SEM from 9 primary ASM cells. ^+++^p < 0.001 vs. 18S; ^*/#^p < 0.05 vs. baseline; ^**/##^p < 0.01 vs. baseline; ^###^p < 0.001 vs. baseline.

Following stimulation with FCS (2.5%) 525 mRNA were increased in expression and 680 were decreased (Additional file 1: Table S1 & S2). Following treatment with Dexamethasone (10^−7^ M) for 1 h before subsequent stimulation with FCS (2.5%) 436 mRNA were increased in expression and 457 were decreased (Additional file 1: Table S3 & S4). Interestingly, only 5 mRNAs were differentially expressed in primary ASM cells after treatment with FCS or dexamethasone + FCS. The pre-treatment of the ASM cells with dexamethasone, prevented the subsequent change induced by FCS as confirmed by RT-PCR (Figure 2 and Table 2). For the first time, we report here the differential expression of these mRNAs in primary ASM cells.

**Table 2:** Differential mRNA expression changes in healthy ASM cells after treatment with FCS or Dex + FCS

**mRNA**	**FCS**		**Dex + FCS**	
	**Microarray (FC)**	**qRT-PCR (FC)**	**Microarray (FC)**	**qRT-PCR (FC)**
FAM71F1	−1.7 (<.05)	−1.62 (<.05)	1.7 (<.05)	1.56 (<.05)
METTL7A	−1.9 (<.01)	−0.85 (<.05)	2.1 (<.01)	5.77 (<.01)
PLSCR4	−1.5 (<.001)	−0.33 (<.05)	1.6 (<.001)	6.47 (<.001)
TUBB3	1.5 (<.05)	1.22 (<.05)	−1.7 (<.001)	−2.18 (<.05)
WDFY4	−1.8 (<.05)	−3.60 (<.01)	1.8 (<.05)	2.01 (<.05)

This table lists those mRNAs that were differentially expressed in healthy ASM cells following stimulation with FCS (2.5%) or treatment with dexamethasone (10^−7^ M) before stimulation with FCS (2.5%). Microarray data are denoted as the fold change against baseline control and is the mean of 9 individual patients. These values are derived from Additional file 1: Table S1, S2, S3, S4 and are for those genes that demonstrated an FDR of less than 0.05; associated P values are shown in parentheses. FC, Fold change.

To identify the pathways that these mRNAs are involved in, we analysed the changes in profile of mRNA expression using the bioinformatics database, DAVID 6.7 (http://david.abcc.ncifcrf.gov/) [13,14]. Significantly, this showed a deregulation of those mRNAs involved in inflammatory and other signalling pathways in primary ASM cells following stimulation with FCS (2.5%). These included those involved in ASM cell signalling (the NOD-like receptor signalling pathway, cell cycle and p53 signalling pathway) and inflammation (Cytokine-cytokine receptor interaction and the TGF-β signaling pathway). Interestingly, when the same ASM cells were pre-treated with dexamethasone (10^−7^ M) before being stimulated with FCS (2.5%), these pathways were not activated, and instead normal cell function was resumed (e.g. phenylalanine and tyrosine metabolism) (Table 3).

**Table 3:** Pathway analysis in primary ASM cells

**Gene set name**	P-value*
*2.5% FCS*	
Cell cycle	0.0045
NOD-like receptor signalling pathway	0.0046
Cytokine-cytokine receptor interaction	0.05
P53 signalling pathway	0.03
TGF-β signalling pathway	0.02
*2.5% FCS + 10*^*−7*^ *M dexamethasone*	
Phenylalanine metabolism	0.011
Tyrosine metabolism	0.04

Pathway analysis of those mRNAs changed in expression using the bioinformatics database, DAVID 6.7 (http://david.abcc.ncifcrf.gov/). *Modified Fisher Exact P-Value, EASE Score (Maximum Probability). The threshold of EASE Score, a modified Fisher Exact P-Value, for gene-enrichment analysis. Fisher Exact P-Value = 0 represents perfect enrichment. P-Value is equal or smaller than 0.05 to be considered strongly enriched in the annotation categories. Default is 0.1.

### miRNA expression levels in primary ASM cells and their respective targets

**Figure 3:**
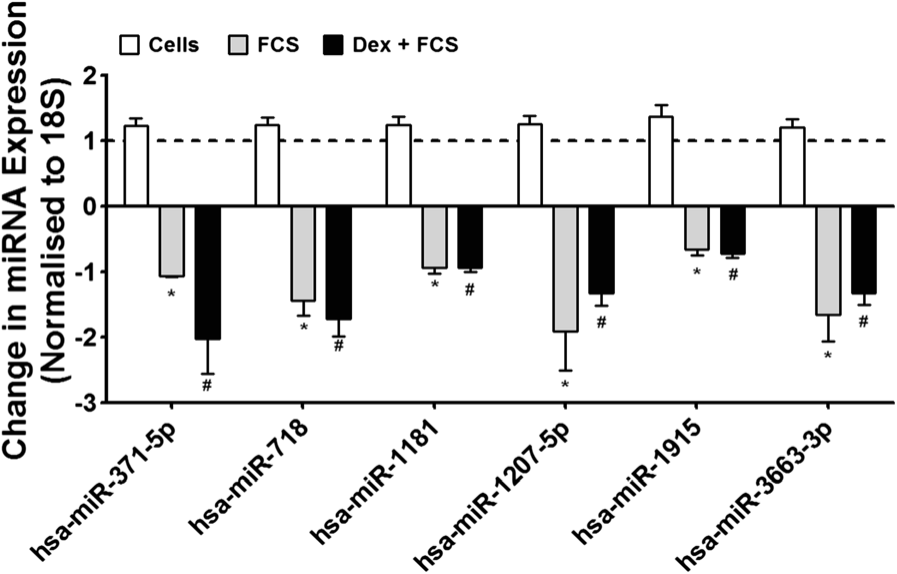
**Effect of dexamethasone and FCS upon miRNA expression in primary ASM cells.** ASM cells were incubated with dexamethasone (10^−7^ M) for 1 h before being stimulated with FCS (2.5%) for 24 h. To validate the array data, the expression of 6 miRNAs were confirmed by TaqMan RT-PCR. Bars represent mean ± SEM from 9 primary ASM cells. ^*/#^p < 0.05 vs. baseline.

We extracted RNA from ASM cells and measured the expression of the miRNAs. At baseline, we detected 230 miRNAs (Additional file 1: Table S5). Following exposure to FCS (2.5%), 10 of these were decreased in expression; a further 3 more were decreased in expression when the ASM cells were exposed to dexamethasone prior to addition of FCS (Table 4). We determined the expression of 6 of these miRNAs by RT-PCR and confirmed that the expression of miR-371-5p, −718, −1181, −1207-5p, −1915, and −3663-3p were reduced by dexamethasone and FCS (Figure 3).

**Table 4:** miRNA expression changes in healthy ASM cells after treatment with FCS or Dex + FCS

**miRNA**	**Baseline expression (ΔCT)**	**FCS**		**Dex + FCS**	
		**Microarray (FC)**	**qRT-PCR (FC)**	**Microarray (FC)**	**qRT-PCR (FC)**
hsa-miR-134	6.1 (<.001)	-	-	−1.5 (<.001)	−1.32 (<.05)
hsa-miR-150*	6.2 (<.001)	-	-	−1.5 (<.001)	−1.25 (<.05)
hsa-miR-371-5p	6.3 (<.001)	−1.6 (<.001)	−0.97 (<.05)	−1.7 (<.001)	−1.73 (<.05)
hsa-miR-718	5.4 (<.001)	−1.5 (<.001)	−1.26 (<.05)	−1.6 (<.001)	−1.44 (<.05)
hsa-miR-762	7.6 (<.001)	−1.7 (<.001)	-	−1.9 (<.001)	-
hsa-miR-940	6.7 (<.001)	−1.5 (<.001)	-	−1.6 (<.001)	-
hsa-miR-1181	7.0 (<.001)	−1.7 (<.001)	−0.89 (<.05)	−1.8 (<.001)	−0.89 (<.05)
hsa-miR-1207-5p	7.7 (<.001)	−1.8 (<.001)	−1.73 (<.05)	−2.0 (<.001)	−1.13 (<.05)
hsa-miR-1915	9.0 (<.001)	−1.8 (<.001)	−0.58 (<.05)	−1.9 (<.001)	−0.66 (<.05)
hsa-miR-3663-3p	8.0 (<.001)	−1.8 (<.001)	−1.10 (<.05)	−2.0 (<.001)	−1.36 (<.05)
hsa-miR-3665	9.7 (<.001)	−2.1 (<.001)	-	−2.3 (<.001)	-
hsa-miR-4271	6.1 (<.001)	-	-	−1.5 (<.001)	-
hsa-miR-4281	9.6 (<.001)	−1.6 (<.001)	-	−1.5 (<.001)	-

This table lists those miRNAs that were differentially expressed in healthy ASM cells following stimulation with FCS (2.5%) or treatment with dexamethasone (10–7 M) before stimulation with FCS (2.5%). Baseline expression levels are expressed as Δ cycle threshold (ΔCT) and have been normalized against RNU44. Microarray data are denoted as the fold change against baseline control and is the mean of 9 individual patients. These values are for those genes that demonstrated an FDR of less than 0.05; associated P values are shown in parentheses. FC, Fold change.

**Figure 4:**
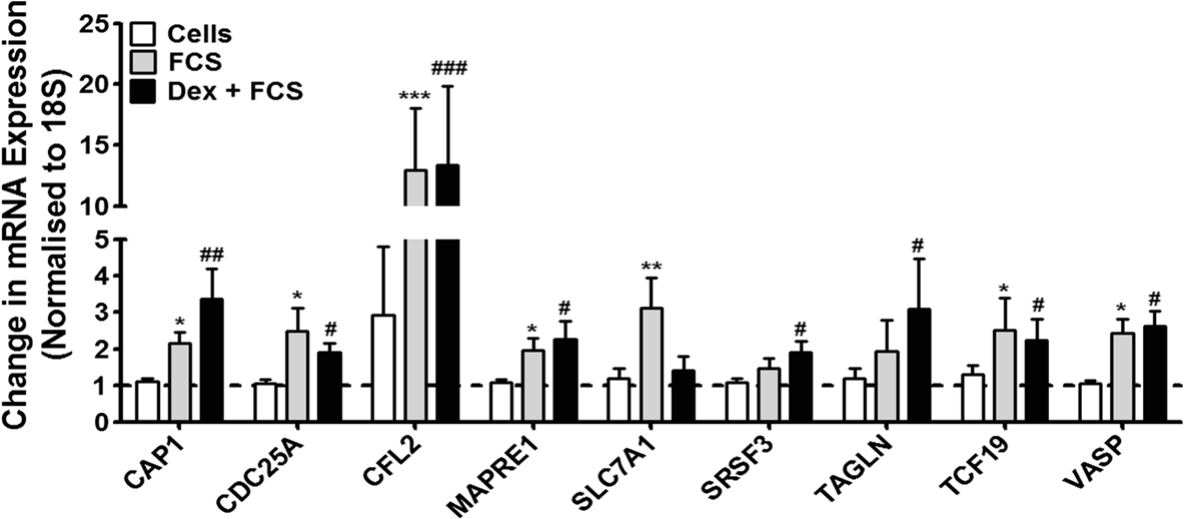
**Effect of dexamethasone and FCS upon the predicted mRNA targets of miRNAs decreased in expression in primary ASM cells.** ASM cells were incubated with dexamethasone (10^−7^ M) for 1 h before being stimulated with FCS (2.5%) for 24 h. 9 mRNAs were changed in expression following treatment. To validate the array data, the expression of these mRNAs were confirmed by TaqMan RT-PCR. Bars represent mean ± SEM from 9 primary ASM cells. ^*/#^p < 0.05 vs. baseline; ^**/##^p < 0.01 vs. baseline; ^###^p < 0.001 vs. baseline.

We utilized the miRNA target prediction program TargetScan (http://www.targetscan.org/) to review the targets of these miRNAs. Of the miRNAs *decreased* in expression following FCS, 3 have predicted mRNA targets that were subsequently *increased* in expression (Additional file 1: Table S1 & S3). To identify the pathways that these mRNAs are involved in, we analysed the changes in profile of mRNA expression using the bioinformatics database, DAVID 6.7 (http://david.abcc.ncifcrf.gov/) [13,14]. This showed that these mRNAs are essential in regulation of the actin cytoskeleton, the remodelling of which is an important mechanism of airway smooth muscle contraction [18]. We confirmed the increased expression of these mRNAs by RT-PCR (Figure 4).

### lncRNA expression levels in primary ASM cells

Long noncoding RNAs may regulate multiple biological pathways that could lead to the development of disease. Currently, lncRNAs can be broadly divided into 4 families based on their sequence and relative position to the exonic regions of protein-coding sequences and include pseudogenes, natural antisense (to exonic regions), intronic lncRNAs, and intergenic lncRNAs [19]. To identify novel lncRNAs, we used ENSEMBLE (http://www.ensembl.org/index.html) to determine the genomic position of those probe sets from the microarray that did not match known protein-coding genes.

**Figure 5:**
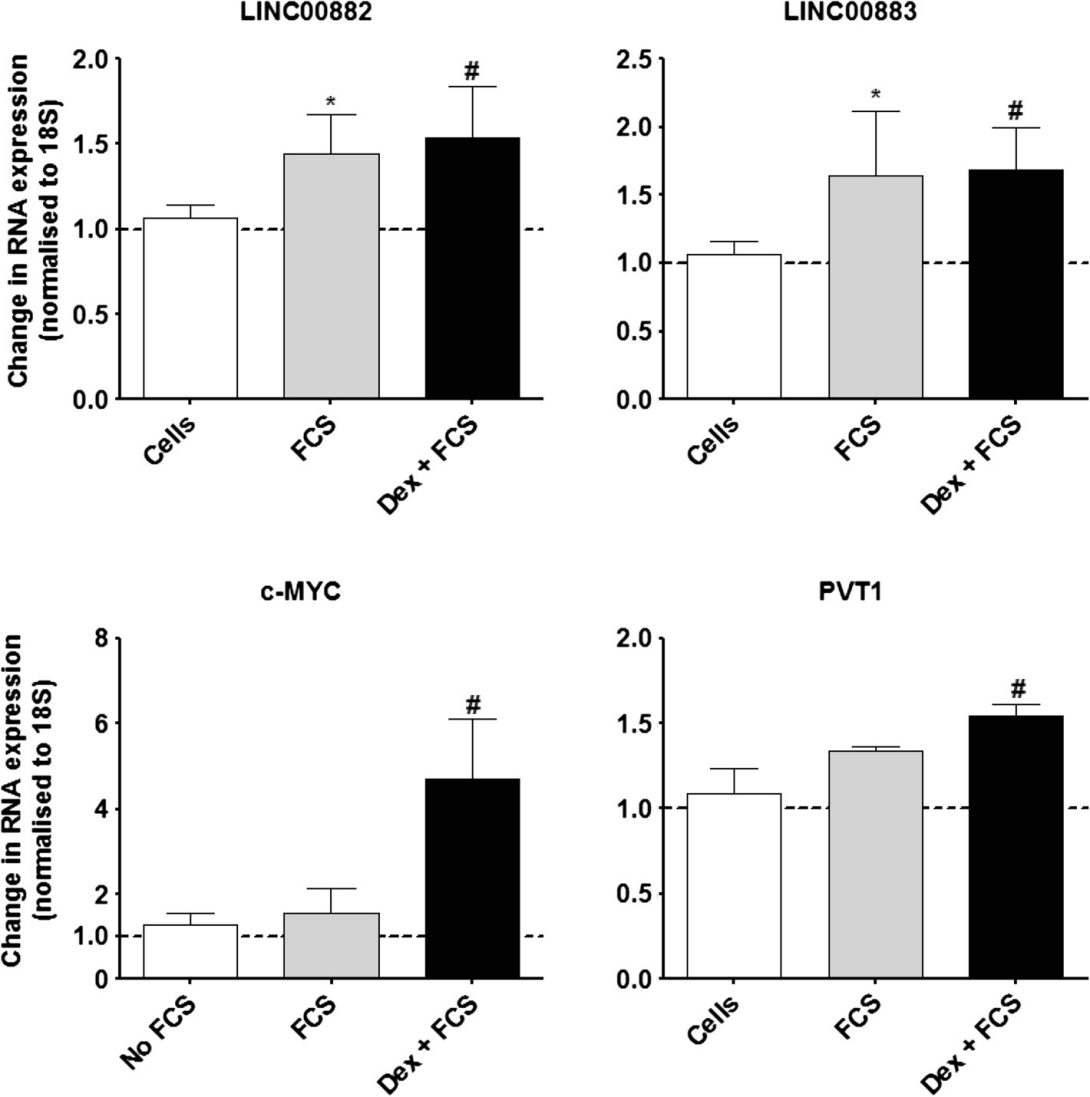
**Effect of dexamethasone and FCS upon lncRNA expression in primary ASM cells.** ASM cells were incubated with dexamethasone (10^−7^ M) for 1 h before being stimulated with FCS (2.5%) for 24 h. To validate the array data, the expression of LINC00882, LINC00883, c-MYC and PVT1 were confirmed by TaqMan RT-PCR. Bars represent mean ± SEM from 9 primary ASM cells. ^*/#^p < 0.05.

Following stimulation with FCS, 17 lncRNAs were increased (Additional file 1: Table S6) and 40 lncRNAs were decreased in expression (Additional file 1: Table S7). In the presence of dexamethasone (10^−7^ M) and FCS, the lncRNA expression profile changed dramatically with 27 lncRNAs increasing and 39 decreasing in expression (Additional file 1: Tables S8 & S9, respectively). Interestingly, 29 lncRNAs were altered after FCS and after dexamethasone + FCS (Table 5). We confirmed the increased expression of LINC00882, LINC00883, and PVT1 oncogene by RT-PCR, and found them to be significantly increased in expression in primary ASM cells that had been pre-treated with dexamethasone (10^−7^ M), before being stimulated with FCS (2.5%) (P < 0.05) (Figure 5). Furthermore, PVT1 is known to be transcriptionally activated by the oncogene c-MYC and p53 both of which were increased in expression in our screen of mRNAs, and we confirmed the expression of c-MYC by RT-PCR, and in a similar pattern to the PVT1 we found it to be significantly increased in expression in primary ASM cells that had been pre-treated with dexamethasone, before being stimulated with FCS (p < 0.05) (Figure 5).

**Table 5:** Common lncRNA expression changes in healthy ASM cells after treatment with FCS or Dex + FCS

			**FCS**	**Dex + FCS**
**Class of lncRNA**	**Ensemble gene ID**	**Transcript**	**Microarray (FC)**	**Microarray (FC)**
Known sense overlapping	ENST00000314957	CTD-2201E18.3	−1.73 (<.01)	1.78 (<.05)
lincRNA	ENST00000414120	LINC00887	−1.81 (<.05)	−1.93 (<.01)
Known antisense	ENST00000415647	RP11-46A10.4	−1.62 (<.05)	−1.65 (<.05)
lincRNA	ENST00000417485	AC012668.2	−2.28 (<.01)	−1.81 (<.01)
lincRNA	ENST00000418539	BCYRN1	−1.53 (<.05)	2.37 (<.05)
Known antisense	ENST00000420095	LMCD1-AS1	−1.57 (<.05)	5.65 (<.05)
lincRNA	ENST00000426635	LINC00472	1.86 (<.05)	2.64 (<.05)
lincRNA	ENST00000431759	SLC2A1-AS1	−1.95 (<.05)	−2.09 (<.05)
lincRNA	ENST00000433843	SNHG5	−1.74 (<.05)	−1.51 (<.01)
lincRNA	ENST00000442316	AC074363.1	−1.60 (<.05)	−1.62 (<.05)
lincRNA	ENST00000444958	DANCR	2.59 (<.05)	2.38 (<.05)
Known antisense	ENST00000446423	SDCBP2	−1.82 (<.05)	−1.82 (<.05)
lincRNA	ENST00000448738	RP1-295F6.2	1.62 (<.05)	1.53 (<.05)
lincRNA	ENST00000451910	RP11-344J7.2	2.06 (<.01)	1.66 (<.05)
Known retained intron	ENST00000453698	SNHG11	−2.85 (<.01)	−2.24 (<.01)
lincRNA	ENST00000473636	LINC00882	2.14 (<.05)	2.08 (<.05)
lincRNA	ENST00000495228	LINC00883	1.63 (<.05)	1.78 (<.05)
lincRNA	ENST00000505448	RP11-774O3.3	−1.57 (<.001)	−1.66 (<.05)
Known antisense	ENST00000507963	NR2F1-AS1	−1.66 (<.05)	−1.61 (<.05)
Known antisense	ENST00000509179	MEF2C-AS1	−2.59 (<.05)	−2.16 (<.01)
lincRNA	ENST00000515296	CTB-35F21.1	−1.83 (<.05)	−1.94 (<.05)
lincRNA	ENST00000515306	CTB-35F21.2	−2.23 (<.05)	−2.15 (<.05)
lincRNA	ENST00000515871	CTC-325J23.3	1.60 (<.05)	1.72 (<.01)
lincRNA	ENST00000518765	RP11-527N22.1	−1.69 (<.05)	−1.53 (<.05)
lincRNA	ENST00000518771	RP11-37B2.1	−1.85 (<.001)	−1.71 (<.001)
lincRNA	ENST00000521128	CTB-43E15.3	−2.07 (<.05)	−2.06 (<.05)
lincRNA	ENST00000523242	CTB-43E15.1	−1.79 (<.05)	−2.02 (<.05)
lincRNA	ENST00000523328	PVT1	1.39 (<.01)	1.66 (<.001)
lincRNA	ENST00000538996	AP003486.1	−2.69 (<.01)	−1.81 (<.05)

This table lists those lncRNAs that have been manually annotated in the Ensembl database and the expression of which is significantly changed in healthy ASM cells after stimulation with FCS (2.5%) or treatment with dexamethasone (10^−7^ M) before stimulation with FCS (2.5%). Microarray data are denoted as the fold change against baseline control and is the mean of 9 individual patients. These values are for those genes that demonstrated an FDR of less than 0.05; associated P values are shown.

Finally, we reviewed the possibility of some of these expressed lncRNAs acting as miRNA ‘sponges’, a concept well documented (69). For possible miRNA–lncRNA interactions, we consulted the online database LNCipedia 2.0 (http://lncipedia.org/). We found that 4 lncRNAs were predicted to act as miRNA ‘*sponges*’ for miRNAs that were detected in our screen. For example, RP11-46A10.4 has complementary regions for miR-1207 (12 sites) and −150 (4 sites). LINC00883 has a binding region for miR-150 (6 sites), BCYRN1 is proposed to be complementary for miR-150 (49 sites), −940 (14 sites) and −371 (18 sites), and LINC00882 is complementary for miR-371 (7 sites). However, to confirm these interactions, subsequent binding studies will need to be undertaken.

## Discussion

We have made several important observations in the expression profiles of non-coding RNAs, including mRNAs, miRNAs and lncRNAs in primary human ASM cells, following stimulation with a well-characterised inducer of proliferation and inflammatory mediator release (FCS), and treatment with a corticosteroid (dexamethasone).

Of all the mRNAs that we examined, only 5 were differentially expressed following treatment. These included a methyltransferase (METTL7A) which is a type of transferase enzyme that transfers a methyl group from a donor to an acceptor, a scramblase (PLSCR4) which is a protein responsible for the translocation of phospholipids between the two monolayers of a lipid bilayer of a cell membrane [20], a member of the tubulin family (TUBB3) which plays a critical role in proper axon guidance and maintenance and is expressed in non-small cell lung cancer [21] and WDFY4, a protein with unknown function but is predominantly expressed in primary and secondary immune tissues [22]. Of those mRNA changed in expression, we have previously reported upon the action of many of these in primary human ASM cells. For example, disintegrin and metalloprotease proteins (ADAMs) inhibit ASM cell adhesion and migration through the β(1)-integrin [23]. Here, we demonstrate others to be increased in expression after stimulation with FCS (2.5%) (ADAM19 & ADAMTS4) (Additional file 1: Table S1), and two more were decreased in expression after stimulation with FCS (2.5%) (ADAM8 & ADAMTS10) (Additional file 1: Table S2) and both of these were further decreased in expression when the ASM cells were pre-treated with dexamethasone (10^−7^ M) before subsequent stimulation with FCS (ADAM8 & ADAMTS10) (Additional file 1: Table S4). Furthermore, 4 additional ADAMs were increased in expression when the ASM cells were pre-treated with dexamethasone (10^−7^ M) before subsequent stimulation with FCS (ADAM28, ADAMTS1, ADAMTS4, and ADAMTS9), and the previously up-regulated ADAM19 was further increased in expression (Additional file 1: Table S3). ASM cells pre-treated with dexamethasone (10^−7^ M) before subsequent stimulation with FCS resulted in the decreased expression of a further 3 (ADAMTS14, ADAMTS19, and ADAMTSL1) (Additional file 1: Table S4). Furthermore, we have previously demonstrated that CCL11 (Eotaxin), is not only released in higher concentrations from ASM cells of patients with asthma compared to nonasthmatic control subjects, but also that dexamethasone can’t repress this release effectively in asthmatic patients [2]. This comprehensive screen of mRNAs shows that CCL11 is decreased in expression in response to FCS stimulation (Additional file 1: Table S2) and pre-treatment with dexamethasone before stimulation results in a further decrease in expression (Additional file 1: Table S4). Interestingly, activation of NF-κB may be the basis for increased expression of many inflammatory genes and for the perpetuation of chronic airway inflammation in asthma [24]. Once again, we show that 2 mRNAs essential for the de-activation of NF-κB are decreased in expression following stimulation with FCS. This includes NFKBIE, which binds to components of NF-kappa-B, trapping the complex in the cytoplasm and preventing it from activating genes in the nucleus, and NFKBIZ, involved in regulation of NF-κB transcription factor complexes (Additional file 1: Table S2). ASM cells are able to express the toll-like receptors, TLR1 to TLR10 via different stimuli. We demonstrated that dexamethasone inhibited cytokine- and ligand-induced TLR2, TLR3, and TLR4 expression and chemokine release. However, dexamethasone potentiated TLR2 expression induced by combined IFN-gamma and TNF-alpha stimulation [25]. Here we see that TLR2 is decreased in expression following stimulation with FCS (Additional file 1: Table S2) and is further reduced when the cells are pre-treated with dexamethasone before said stimulation (Additional file 1: Table S4). Interestingly, TLR4 was increased in expression when the cells are pre-treated with dexamethasone before said stimulation (Additional file 1: Table S3). Additionally, we have previously demonstrated that IRAK-1 mRNA expression is significantly decreased in expression following IL-1β stimulation, in ASM cells [17]. Here we show that ASM cells pre-treated with dexamethasone before stimulation with FCS causes an increase in expression of IRAK1 (Additional file 1: Table S3). Here we demonstrate that FCS induces IL-6 release from primary ASM cells. We have previously found that TGF-β induced the expression of NOX4 and was accompanied by elevated IL-6 release in ASM cells [26]. Further studies revealed a role for SMAD3 pathways. ASM cells treated with dexamethasone, before stimulation with FCS, further increases the expression of NOX4 (Additional file 1: Table S3), and decreases the expression of SMAD3 (Additional file 1: Table S4). Finally, and most recently, we have previously shown that the cyclin-dependent inhibitor p27^KIP1^ (CDKN1B) is vitally important in driving the hyperproliferation seen in ASM cells from asthmatics [7]. Here we see that cells treated with dexamethasone before stimulation with FCS show a decreased expression in this inhibitor (Additional file 1: Table S4).

We then proceeded to measure the expression of miRNAs in our human primary ASM cells. Over 200 miRNAs were detected, and we detected a change in the expression of a handful of these miRNAs following subsequent stimulation with FCS and treatment with dexamethasone. All of these miRNAs have previously been shown to be associated with cancer, for example, miR-371-5p is associated with gastric cancer [27], miR-718 with breast cancer [28], miR-1181 with ovarian cancer [29], miR-1207-5p is associated with a novel cancer treatment, isoliquiritigenin [30], miR-1915 is associated with drug resistance in colorectal cancer [31] and miR-3663-3p is associated with skin cancer [32]. Although these miRNAs have a role in aberrant cellular proliferation, this is the first time that these miRNAs have reported to be expressed in proliferating primary ASM cells. However, miR-1915 and −1207 have been previously reported to be increased in expression in asthmatic epithelial cells from airway brushings [33], and miR-1207 has been shown to be associated with vascular smooth muscle proliferation [34]. Furthermore, we propose that the decreased expression of three of these miRNAs (miR-371-5p, −940 and −1207-5p), allows for the expression of their target mRNAs which are involved in critical aspects of ASM cell cycle regulation such as regulation of microtubule structure, actin-filament dynamics and later stage cell cycle progression, although targeting studies need to be performed to confirm this [35-43].

lncRNAs are widely expressed and yet their importance in physiological and pathological response has only recently been elucidated [8,44]. They are known to have a mechanism of action upon activation of common transcription factors such as NF-κB, Sox2, p53, Oct4 and Nanog [45]. We detected the altered expression of 29 lncRNAs following FCS stimulation, and dexamethasone + FCS treatment, including small nucleolar RNA host gene 5 (previously U50HG), known to be expressed in human B-cell lymphoma [46]; SDCBP2 antisense which is increased in expression in non-small cell lung cancer [47]; and small nucleolar host gene 11. 8 lncRNAs were increased in expression, including LINC00472 which is expressed in renal tissue [48] and small nucleolar host gene 13 (also known as DANCR) and 3 lncRNAs were differentially expressed between stimulation with FCS and those with dexamethasone and FCS which included brain cytoplasmic RNA 1 (also known as BC200) which is highly expressed in the hypothalamus [49] and is deregulated in lung cancer [50], and two novel lncRNAs; ENST00000314957 AND ENST00000420095. We validated our array data by confirming the expression of PVT1, and its transcriptional activator c-MYC. This lncRNA has been previously implicated in diabetes and cancer [51-53]. It has been demonstrated that PVT1 is overexpressed in ovarian and breast cancer cells and when this expression is inhibited by siRNAs, cell proliferation is decreased and apoptosis increased [53]. PVT1 is a downstream target of c-MYC that targets and binds PVT1, subsequently driving its transcription [52]. Interestingly both c-MYC and PVT1 are part of the same locus known to be frequently overexpressed in cancer [53] and c-MYC is an important oncogene known to increase cancer cell growth and proliferation [54]. Furthermore, it is known that PVT1 expression can be directly induced by p53, as well as expressing a cluster of 6 miRNAs, including miR-1207-5p, which as stated above is decreased in expression in the primary ASM cells [55].

**Figure 6:**
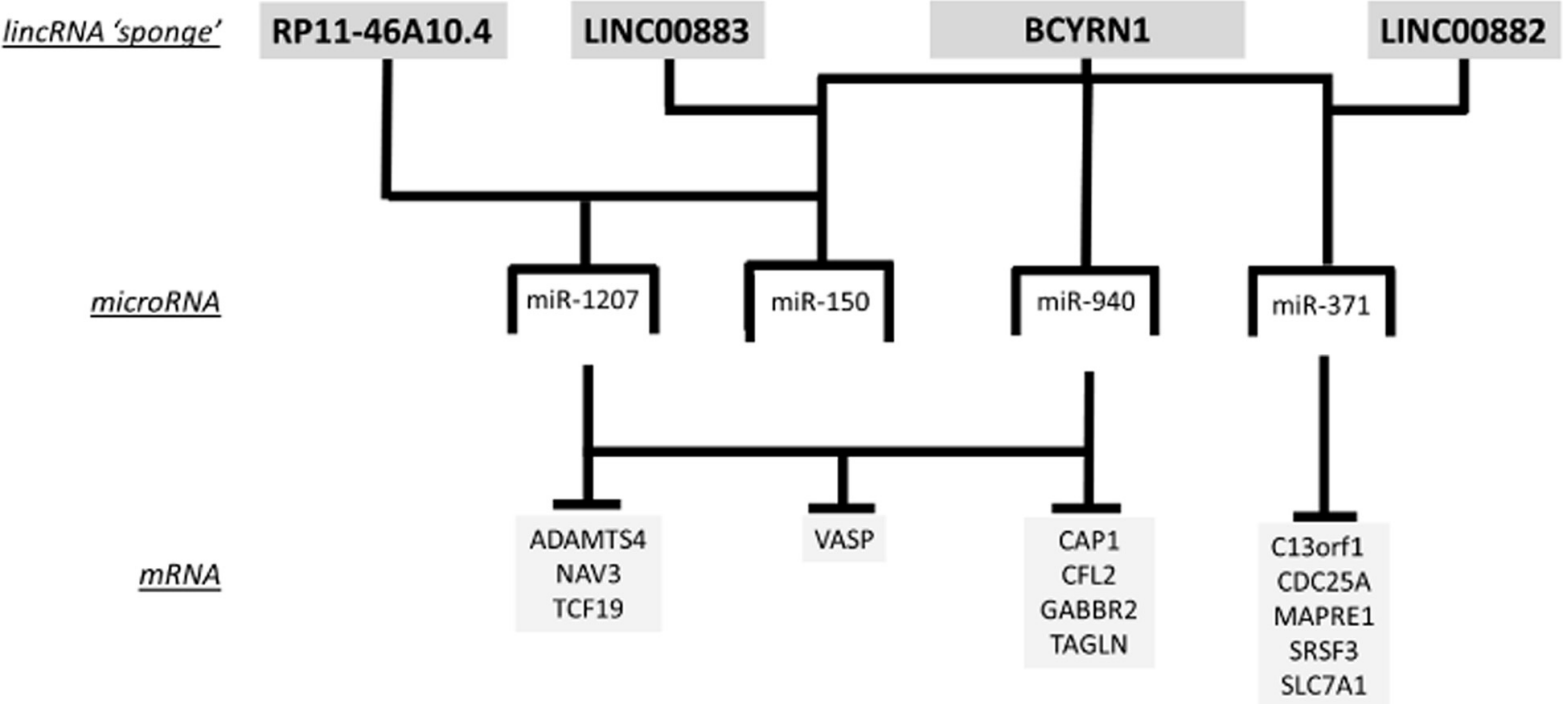
**Proposed ncRNA interactions in primary airway smooth muscle cells following treatment with dexamethasone before stimulation with FCS.** ASM cells were incubated with dexamethasone (10^−7^ M) for 1 h before being stimulated with FCS (2.5%) for 24 h. The lincRNAs RP11-46A10.4, LINC00883, BCYRN1 and LINC00882 act as ‘sponges’ for the miRNAs −1207, −150, −940 and −371. This, in turn, allows for the mRNA targets of these miRNAs to be expressed.

Finally, we address the possibility of lncRNAs acting as miRNA ‘*sponges*’, a concept well documented [56]. We utilized the online data base LNCipedia 2.0 (http://lncipedia.org/) and found that that 4 lncRNAs were predicted to act as miRNA ‘*sponges*’ for those miRNAs, that were seen to be decreased in expression. We propose that the lincRNAs RP11-46A10.4, LINC00883, BCYRN1 and LINC00882 act as *‘sponges’* for the miRNAs −1207, −150, −940 and −371 and that this allows for the mRNA targets of these miRNAs to be expressed, as summarised in Figure 6. Again, these interactions need to be confirmed with knock-down studies.

In summary, using a transcriptomics-based approach, we have demonstrated that primary human ASM cells have a transcript expression profile associated with an activated phenotype and have shown that these changes might, in part, be mediated through alterations in noncoding RNA expression. Clearly, it is important to confirm these observations by examining the expression of these lncRNAs in primary ASM cells from patients with asthma and then to follow up these observations by targeting specific lncRNAs (such as gain and loss-of-function studies). These studies are currently on-going.

## Competing interests

The authors declare that they have no competing interests.

## Authors’ contributions

MMP, cultured the ASM cells, analyzed the array data, performed the RT-PCRs and drafted the manuscript. ET performed the arrays. PJA performed some of the RT-PCRS. MAL, helped analyze the array data, and helped conceive the study. DSG performed the patient biopsies. IMA & KFA helped with writing the manuscript. All authors read and approved the final manuscript.

## Supplementary Material

Additional file 1: Table S1 Up-regulated mRNAs in healthy ASM cells after stimulation with FCS. **Table S2.** Down-regulated mRNAs in healthy ASM cells after stimulation with FCS. **Table S3.** Up-regulated mRNAs in healthy ASM cells after stimulation with Dex + FCS. **Table S4.** Down-regulated mRNAs in healthy ASM cells after stimulation with Dex + FCS. **Table S5.** Baseline levels of miRNAs in healthy ASM. **Table S6.** lncRNAs increased in expression in healthy ASM cells after stimulation with FCS. **Table S7.** lncRNAs decreased in expression in healthy ASM cells after stimulation with FCS. **Table S8.** lncRNAs increased in expression in healthy ASM cells after stimulation with Dex + FCS. **Table S9.** Changes in expression of lncRNAs in healthy ASM cells after stimulation with Dex + FCS. Click here for file
