# Supplementary material for: Role of non-coding RNAs in maintaining primary airway smooth muscle cells
Source: Respir Res. 2014 May 16;15(1):58. doi: 10.1186/1465-9921-15-58 (PMC4039655; doi:10.1186/1465-9921-15-58)
Supplement: Additional file 1: Table S1 — Up-regulated mRNAs in healthy ASM cells after stimulation with FCS. Table S2. Down-regulated mRNAs in healthy ASM cells after stimulation with FCS. Table S3. Up-regulated mRNAs in healthy ASM cells after stimulation with Dex + FCS. Table S4. Down-regulated mRNAs in healthy ASM cells after stimulation with Dex + FCS. Table S5. Baseline levels of miRNAs in healthy ASM. Table S6. lncRNAs increased in expression in healthy ASM cells after stimulation with FCS. Table S7. lncRNAs decreased in expression in healthy ASM cells after stimulation with FCS. Table S8. lncRNAs increased in expression in healthy ASM cells after stimulation with Dex + FCS. Table S9. Changes in expression of lncRNAs in healthy ASM cells after stimulation with Dex + FCS. [file 1465-9921-15-58-S1.docx]

# *Online Repository*

**Role of long non-coding RNAs In Maintaining Primary Airway Smooth Muscle Cells**

Mark M. Perry PhD^1*^, Eleni Tsitsiou PhD^2^, Philip J. Austin MSc^1^, Mark A. Lindsay PhD^,2,3^, David S. Gibeon MD^1^, Ian M Adcock^1^ and Kian Fan Chung MD^1^

**Additional Table 1: Up-regulated mRNAs in healthy ASM cells after stimulation with FCS.**

| **Gene Symbol** | ***P* value** | **Fold-Change** |
| --- | --- | --- |
| A2LD1 | 0.0473568 | 1.59523 |
| ABCC3 | 0.00359799 | 1.77518 |
| ABL2 | 0.0217408 | 1.68963 |
| ACTA2 | 0.00310887 | 2.89568 |
| ACTBL2 | 0.000356002 | 2.45771 |
| ACTG2 | 0.000982242 | 5.20555 |
| ACTN1 | 0.00263887 | 1.90799 |
| ACTN3 | 0.00895882 | 2.17395 |
| ACTR2 | 0.0181474 | 1.56134 |
| ACTR3 | 0.00129864 | 1.80328 |
| ADAM19 | 0.0124993 | 1.58407 |
| ADAMTS4 | 0.017624 | 1.88224 |
| ADAT1 | 0.0458691 | 1.51365 |
| AFMID | 0.0361731 | 1.61357 |
| ALDH1B1 | 0.00320423 | 2.04631 |
| AMIGO2 | 0.001229 | 1.62668 |
| ANLN | 0.0232678 | 2.60045 |
| AP1S3 | 0.0126273 | 2.11714 |
| ARHGAP11B | 0.00167626 | 2.32391 |
| ARHGDIB | 0.0119549 | 2.81529 |
| ARPC5 | 0.0106956 | 1.66691 |
| ARRDC4 | 0.0401313 | 1.50346 |
| ARSJ | 0.0214678 | 1.96761 |
| ASB2 | 0.0127708 | 1.98642 |
| ASF1B | 0.00848076 | 2.45925 |
| ASNS | 0.0350537 | 1.70716 |
| ASPM | 0.0352247 | 1.86709 |
| ATP8B1 | 0.00258924 | 1.63101 |
| AURKA | 0.00831511 | 3.0711 |
| AX747335 | 0.0102838 | 2.27068 |
| BLM | 0.00625749 | 3.68027 |
| BOLA3 | 0.00479253 | 1.74646 |
| BRI3BP | 0.0129721 | 2.02912 |
| BUB1B | 4.15E-06 | 4.48645 |
| BYSL | 0.0260106 | 1.51964 |
| C11orf24 | 0.00530953 | 1.55003 |
| C11orf82 | 0.0352926 | 3.57566 |
| C11orf94 | 0.0127201 | 1.78834 |
| C12orf4 | 0.00307875 | 1.52656 |
| C12orf75 | 0.0241044 | 1.52064 |
| C13orf1 | 0.00171324 | 1.80636 |
| C13orf15 | 0.00410959 | 1.61763 |
| C13orf34 | 0.0105848 | 2.2299 |
| C13orf37 | 0.0155602 | 1.72628 |
| C14orf142 | 0.0221392 | 2.34922 |
| C14orf145 | 0.0194211 | 1.96543 |
| C14orf80 | 0.00786414 | 1.93243 |
| C15orf23 | 0.0388462 | 1.93152 |
| C16orf59 | 0.00924119 | 2.10041 |
| C16orf87 | 0.0457798 | 1.54013 |
| C17orf60 | 0.00311916 | 1.73688 |
| C1GALT1 | 0.00326165 | 1.53913 |
| C1orf112 | 0.0294664 | 1.61584 |
| C1orf152 | 0.0389023 | 1.56979 |
| C1orf201 | 0.0111578 | 1.79828 |
| C4orf49 | 0.0222313 | 1.86003 |
| C5orf30 | 0.0106706 | 1.63476 |
| C7orf40 | 0.0201262 | 1.61206 |
| CA6 | 0.00624875 | 1.6645 |
| CALM2 | 0.00159172 | 1.72008 |
| CAP1 | 0.0100102 | 1.64071 |
| CASC5 | 0.00111404 | 2.82695 |
| CAV2 | 0.00815085 | 1.52694 |
| CCDC18 | 0.0415637 | 1.61074 |
| CCNB1 | 0.0267928 | 2.29475 |
| CCNE2 | 0.0197001 | 3.47304 |
| CCNYL1 | 0.0143844 | 1.83486 |
| CD3EAP | 0.00127532 | 2.00972 |
| CDC25A | 0.0419207 | 2.12668 |
| CDC45L | 0.0154664 | 2.97072 |
| CDCA3 | 0.0360473 | 1.99193 |
| CDCA5 | 0.0333657 | 2.19942 |
| CDCA7 | 0.0197732 | 1.8144 |
| CDCA8 | 0.000458842 | 3.47452 |
| CDCP1 | 0.00922004 | 1.82445 |
| CDH4 | 0.00488187 | 2.6152 |
| CDK1 | 0.000630106 | 3.05038 |
| CDT1 | 0.00784624 | 3.38734 |
| CECR3 | 0.0350506 | 2.92084 |
| CENPE | 0.0201434 | 2.12556 |
| CENPF | 0.022296 | 2.12717 |
| CENPJ | 0.00490155 | 1.60429 |
| CENPL | 0.0231718 | 2.23116 |
| CENPM | 0.0344694 | 2.00982 |
| CEP55 | 0.0449958 | 2.31017 |
| CEP72 | 0.00470819 | 2.40796 |
| CFL2 | 0.012937 | 1.51145 |
| CHAC1 | 0.027602 | 2.16924 |
| CHAC2 | 0.00919548 | 2.7833 |
| CHCHD3 | 0.0290917 | 1.60975 |
| CHORDC1 | 0.00673106 | 1.53081 |
| CKAP4 | 0.00125666 | 1.60197 |
| CLEC14A | 0.0126479 | 2.40322 |
| CLN6 | 0.00712464 | 1.65789 |
| CMTM4 | 0.00855129 | 1.54562 |
| CNN1 | 0.000314271 | 2.53258 |
| COL28A1 | 0.0353525 | 1.87883 |
| COQ3 | 0.0237535 | 1.62747 |
| CORO2A | 0.0028019 | 1.86528 |
| COTL1 | 0.00431895 | 1.98309 |
| CR592658 | 0.00861652 | 1.54451 |
| CSAG3 | 0.0438812 | 34.8519 |
| CSE1L | 0.036404 | 1.50799 |
| CSRP1 | 0.00674141 | 1.77805 |
| CTGF | 0.0356745 | 2.18414 |
| CTPS | 0.00638484 | 1.92042 |
| CYCS | 0.00514624 | 1.79454 |
| CYR61 | 0.00364933 | 2.36182 |
| DBF4 | 0.0143513 | 1.75725 |
| DCAF13 | 0.0207883 | 1.52838 |
| DCBLD1 | 0.00374507 | 1.50363 |
| DDAH1 | 0.00888684 | 2.14441 |
| DDX21 | 0.0254821 | 1.67479 |
| DDX31 | 0.00174958 | 1.5632 |
| DGKI | 0.0225379 | 1.86663 |
| DHDPSL | 0.00178342 | 1.70514 |
| DHODH | 0.00236091 | 1.77003 |
| DHRS3 | 0.000684298 | 2.05053 |
| DIAPH3 | 0.0148661 | 1.8022 |
| DKK1 | 0.0305069 | 2.88297 |
| DNAJC9 | 0.0462857 | 1.59174 |
| DNMT1 | 0.00745477 | 1.74978 |
| DOCK5 | 0.0280351 | 1.72381 |
| DONSON | 0.000155758 | 1.97775 |
| DPP3 | 0.0270335 | 1.50156 |
| DTL | 0.00244399 | 4.08965 |
| DUS2L | 0.0191879 | 1.51653 |
| DUSP1 | 0.0163001 | 2.0809 |
| DUSP14 | 0.00829877 | 1.70527 |
| DZIP1L | 0.0286215 | 1.55293 |
| E2F2 | 0.0492566 | 2.70585 |
| EBNA1BP2 | 0.0304092 | 1.56539 |
| EDARADD | 0.00236255 | 1.60031 |
| EDN1 | 0.00209379 | 3.21123 |
| EIF4EBP1 | 0.0471212 | 1.53087 |
| EIF5A | 0.00680388 | 1.53539 |
| EL947753 | 0.0217767 | 1.58732 |
| ELK1 | 0.00714714 | 1.98363 |
| ELN | 0.00514957 | 1.65628 |
| ELOVL6 | 0.0125648 | 1.71867 |
| EMB | 0.00237979 | 1.58564 |
| EMG1 | 0.000509884 | 1.66791 |
| ENO1 | 0.0120864 | 1.57344 |
| ENST00000306901 | 0.0492763 | 1.64769 |
| ENST00000319817 | 0.0273014 | 3.43523 |
| ENST00000358726 | 0.0344905 | 1.91685 |
| ENST00000375322 | 0.019868 | 1.68009 |
| ENST00000377561 | 0.0284819 | 2.59618 |
| ENST00000389690 | 0.0211908 | 1.5758 |
| ENST00000390300 | 0.0141332 | 2.19398 |
| ENST00000393215 | 0.0370357 | 2.55066 |
| ENST00000402420 | 0.0349485 | 4.6768 |
| ENST00000402630 | 0.0407533 | 3.04289 |
| ENST00000404580 | 0.0288434 | 1.9597 |
| ENST00000416025 | 0.0492603 | 1.59493 |
| ENST00000421635 | 0.0388962 | 1.71528 |
| EPT1 | 0.0186038 | 1.68156 |
| ERI2 | 0.0279329 | 1.7413 |
| EXOC6 | 0.0135531 | 1.5516 |
| EXOSC2 | 6.10E-05 | 1.72311 |
| F3 | 0.00423372 | 3.17125 |
| FAIM3 | 0.0242383 | 1.68634 |
| FAM105A | 0.00804309 | 2.32542 |
| FAM111B | 0.0416679 | 2.67362 |
| FAM132A | 0.0326316 | 1.67071 |
| FAM136B | 0.00770058 | 1.5015 |
| FAM176A | 0.0293408 | 1.82994 |
| FAM38A | 0.0393296 | 1.50968 |
| FAM83D | 0.0212091 | 3.7806 |
| FAM86B1 | 0.0245849 | 2.00161 |
| FAM98A | 0.0102513 | 1.54278 |
| FANCA | 0.0278484 | 2.01344 |
| FANCB | 0.0455303 | 1.82441 |
| FANCI | 0.0349605 | 1.91718 |
| FARSB | 0.01025 | 1.70789 |
| FBXO5 | 0.0226256 | 1.84115 |
| FEN1 | 0.0241833 | 2.35045 |
| FGF5 | 0.00543621 | 2.03224 |
| FHL2 | 0.00020471 | 1.54355 |
| FICD | 0.0152053 | 1.53836 |
| FJX1 | 0.0129155 | 1.92294 |
| FLJ40504 | 0.00457291 | 4.50374 |
| FLJ42393 | 0.0285267 | 1.67559 |
| FLNB | 0.0023196 | 1.66312 |
| FMN2 | 0.00811326 | 1.84142 |
| FOSL1 | 0.0369381 | 2.22236 |
| FUBP1 | 0.00193569 | 1.64141 |
| FUS | 0.00380931 | 2.26756 |
| GABBR2 | 0.0275003 | 1.89219 |
| GABRP | 0.0121715 | 1.52743 |
| GADD45B | 0.00756349 | 1.94996 |
| GALE | 7.37E-05 | 2.74876 |
| GBP1 | 0.0297375 | 1.9993 |
| GFI1B | 0.0135145 | 1.72586 |
| GGT5 | 0.000113797 | 3.06502 |
| GINS1 | 0.00818464 | 2.51653 |
| GINS3 | 0.020931 | 2.24386 |
| GLIPR1 | 0.0061139 | 1.57793 |
| GMPPB | 0.000802384 | 1.8673 |
| GPATCH4 | 0.0461517 | 1.5258 |
| GPSM2 | 0.0153476 | 1.59027 |
| GREM2 | 0.0153673 | 2.29357 |
| GRPEL1 | 0.000573995 | 1.73288 |
| GTF2H3 | 0.0257689 | 1.64635 |
| GTF2H4 | 0.0415882 | 1.72399 |
| GTPBP4 | 0.0166033 | 1.62195 |
| GTSE1 | 0.000760673 | 3.9854 |
| GYS1 | 0.000334955 | 1.61794 |
| H2AFX | 0.0149799 | 1.91264 |
| HAND1 | 0.0467191 | 2.97065 |
| HAP1 | 0.0249679 | 1.50463 |
| HAPLN3 | 0.0271947 | 1.55199 |
| HAS2 | 0.0149304 | 4.0856 |
| HAUS8 | 0.0351232 | 1.99843 |
| HCFC1 | 0.0323987 | 1.79262 |
| HCRT | 0.0176294 | 1.80459 |
| HEG1 | 0.00988159 | 1.6711 |
| HIF1A | 0.00280494 | 1.63095 |
| HIST1H2AI | 0.048675 | 2.27776 |
| HIST1H2AL | 0.0363451 | 2.40872 |
| HIST1H2BE | 0.0155159 | 2.73909 |
| HIST2H3A | 0.00189325 | 3.49773 |
| HJURP | 0.00808353 | 3.11079 |
| HK2 | 0.00578079 | 1.97009 |
| HMMR | 0.00317915 | 5.30962 |
| HN1 | 0.00586941 | 1.96224 |
| HN1L | 0.0138748 | 1.64134 |
| HNRNPA3 | 0.0250698 | 1.50182 |
| HNRNPAB | 0.000619707 | 1.79505 |
| HSPA2 | 0.0278059 | 2.85522 |
| HSPB7 | 0.0146177 | 1.76472 |
| ID1 | 0.00748058 | 5.78452 |
| ID3 | 0.00019791 | 3.76437 |
| IFRD2 | 0.000832163 | 1.69292 |
| IL7R | 0.0193223 | 3.23351 |
| INTS7 | 0.00280363 | 1.66323 |
| IPO11 | 0.0223085 | 1.77172 |
| IQGAP3 | 0.0365885 | 1.69212 |
| ITGA1 | 0.0022593 | 2.19959 |
| ITGA4 | 0.0138842 | 1.54992 |
| ITPRIPL1 | 0.0128132 | 2.19611 |
| JMJD6 | 0.00808433 | 1.69177 |
| KCNG4 | 0.0393494 | 3.79412 |
| KCNK6 | 0.018762 | 1.93167 |
| KCNN4 | 0.0211664 | 2.02133 |
| KIAA1715 | 0.00411375 | 1.52166 |
| KIAA1919 | 0.0214877 | 2.96947 |
| KIF11 | 0.0310097 | 1.92414 |
| KIF22 | 0.0195312 | 1.77975 |
| KIF23 | 0.00331533 | 2.66493 |
| KIF2C | 0.00173856 | 3.01697 |
| KIF4A | 4.84E-05 | 3.50625 |
| KLF7 | 0.0222497 | 1.69265 |
| KLHDC10 | 0.0155367 | 1.55281 |
| KLHL38 | 0.0374842 | 1.98989 |
| KRT18 | 0.041022 | 2.42737 |
| KRT19 | 4.37E-05 | 2.51543 |
| KRT19P2 | 0.0349763 | 1.7781 |
| KRT34 | 0.0362793 | 3.14486 |
| KRT7 | 0.00523799 | 1.71339 |
| KRT83 | 0.0374577 | 1.98773 |
| KRTAP12-1 | 0.0396133 | 2.75427 |
| KRTAP1-5 | 0.00383542 | 4.81174 |
| LARP4 | 0.000263592 | 1.58878 |
| LDHA | 0.00066032 | 1.77633 |
| LGALS8 | 0.000104249 | 1.6066 |
| LMNB1 | 0.000142366 | 2.79467 |
| LMNB2 | 0.0488896 | 1.78679 |
| LMO7 | 0.0306275 | 1.63992 |
| LOC100128086 | 0.0424341 | 1.64663 |
| LOC100129619 | 0.0363323 | 1.73826 |
| LOC100129700 | 0.0339306 | 1.57644 |
| LOC100130431 | 0.0463175 | 2.06273 |
| LOC100130827 | 0.0194501 | 1.89392 |
| LOC100131150 | 0.0273876 | 1.69618 |
| LOC100132509 | 0.00677071 | 1.87116 |
| LOC100133180 | 0.0252926 | 1.83961 |
| LOC100133264 | 0.0436695 | 1.86075 |
| LOC100133319 | 0.0264913 | 2.43581 |
| LOC100133478 | 0.002359 | 1.86738 |
| LOC145694 | 0.00569387 | 2.08839 |
| LOC387647 | 0.00166725 | 1.51063 |
| LOC389842 | 0.00160689 | 1.84452 |
| LOC390251 | 0.0325124 | 2.75689 |
| LOC391334 | 0.00117074 | 2.17981 |
| LOC401431 | 0.0174611 | 1.70299 |
| LOC442249 | 0.0291224 | 3.96944 |
| LOC442421 | 0.0319633 | 1.97042 |
| LOC554223 | 0.0329583 | 3.21439 |
| LOC642947 | 0.00879634 | 4.89175 |
| LOC643371 | 0.00105322 | 1.99877 |
| LOC644992 | 0.00369325 | 2.00295 |
| LOC646048 | 0.000312218 | 1.79143 |
| LOC646079 | 0.028393 | 2.84243 |
| LOC648740 | 0.000674804 | 1.64145 |
| LOC729378 | 0.0351831 | 2.48492 |
| LOC729454 | 0.00828882 | 1.77104 |
| LOXL2 | 0.0174751 | 1.5495 |
| LPXN | 0.0404443 | 1.8093 |
| LRP8 | 0.0153014 | 2.66328 |
| LRRC42 | 0.00122075 | 1.83798 |
| LRRC59 | 0.00522144 | 1.70432 |
| MANF | 0.0348441 | 1.53087 |
| MAPRE1 | 0.00195632 | 1.63517 |
| MCAM | 0.000803665 | 2.80743 |
| MCM2 | 0.0210543 | 1.93985 |
| MCM3 | 0.042744 | 1.68734 |
| MCM5 | 0.0243171 | 2.10559 |
| MEI1 | 0.0151583 | 4.01627 |
| MELK | 0.0164195 | 2.09686 |
| MID2 | 0.0456738 | 1.78033 |
| MLF1IP | 0.0333624 | 2.09132 |
| MLLT11 | 0.00111098 | 1.55577 |
| MMP24 | 0.00463227 | 2.59074 |
| MND1 | 0.0148934 | 2.66337 |
| MRTO4 | 0.0315914 | 1.58369 |
| MTHFD2 | 0.0098189 | 1.83029 |
| MTRR | 0.039417 | 1.99966 |
| MYL12A | 0.00650341 | 1.68098 |
| MYL6 | 0.00194664 | 1.58301 |
| MYLK | 0.000298128 | 1.68602 |
| MYO19 | 0.00272628 | 1.56646 |
| NAA15 | 0.00348916 | 2.41736 |
| NAV3 | 0.0097082 | 1.82441 |
| NCAPD3 | 0.00136729 | 1.67735 |
| NCAPG2 | 0.00610283 | 4.20294 |
| NCEH1 | 0.00366176 | 2.04831 |
| NEXN | 0.00709487 | 1.65021 |
| NIPSNAP3A | 0.00350686 | 1.58059 |
| NLN | 0.00108945 | 1.90227 |
| NOP16 | 0.00634381 | 2.00852 |
| NOP2 | 0.00374573 | 1.5322 |
| NPAS1 | 0.0307637 | 1.62189 |
| NPPB | 0.00653979 | 2.93824 |
| NQO1 | 0.000445536 | 1.61873 |
| NSL1 | 0.0199937 | 1.54699 |
| NT5DC3 | 0.0374198 | 2.63142 |
| NT5E | 0.0258296 | 1.64599 |
| NUP155 | 0.0184522 | 2.09068 |
| NUP205 | 0.00984885 | 1.53632 |
| NUP35 | 0.0078269 | 1.78598 |
| NUP93 | 0.0222961 | 1.50566 |
| NUPL1 | 0.0044224 | 2.15442 |
| NUSAP1 | 0.0455255 | 1.96689 |
| OC90 | 0.00455022 | 1.59083 |
| ODC1 | 0.0083076 | 1.92795 |
| OIP5 | 0.0182519 | 2.2305 |
| OLIG3 | 0.00427445 | 2.91079 |
| OPN1MW | 0.00118636 | 1.80235 |
| ORC6L | 0.00286673 | 2.10542 |
| P704P | 0.00893802 | 1.75169 |
| PAICS | 0.010631 | 1.50694 |
| PAQR4 | 0.00298978 | 4.0321 |
| PCBD2 | 0.00817189 | 1.50428 |
| PDCL3 | 0.0396062 | 1.54765 |
| PDIA6 | 0.0104978 | 1.58307 |
| PDLIM5 | 0.0126741 | 2.00568 |
| PDSS1 | 0.0255356 | 1.67895 |
| PFKP | 0.00097882 | 2.2745 |
| PGAM5 | 0.0319027 | 1.70017 |
| PGM2 | 0.019898 | 1.56852 |
| PI3 | 0.00813922 | 1.59564 |
| PKD2L1 | 0.0390485 | 1.58832 |
| PKMYT1 | 0.00207547 | 3.1837 |
| PLAUR | 0.0178654 | 2.05979 |
| PLCB2 | 0.0244653 | 2.44511 |
| PNP | 0.017154 | 2.11242 |
| POLA2 | 0.00104333 | 2.17132 |
| POLR2D | 0.00375386 | 1.53654 |
| POLR3G | 0.000846248 | 1.89074 |
| POMP | 0.00386729 | 1.73314 |
| POP1 | 0.00299567 | 1.99151 |
| POTEE | 0.012921 | 1.70344 |
| POTEF | 0.0160526 | 1.89097 |
| POTEKP | 0.0300031 | 1.67653 |
| PPIF | 0.00715156 | 1.73244 |
| PPP1R12B | 0.0129486 | 1.70853 |
| PRC1 | 0.0205059 | 2.17837 |
| PRO2852 | 0.0315912 | 2.11945 |
| PRPS1 | 0.0317039 | 1.57352 |
| PRPS1L1 | 0.00732765 | 1.93722 |
| PSAT1 | 0.0240063 | 2.1948 |
| PSG3 | 0.039584 | 2.08757 |
| PSMD12 | 0.0470892 | 1.50063 |
| PSMD14 | 0.0126347 | 1.5149 |
| PTPLA | 0.00357932 | 1.81451 |
| PTPLB | 0.00342578 | 1.67774 |
| PUS1 | 0.00936641 | 1.74233 |
| PUS7 | 0.0154351 | 1.70272 |
| PVR | 0.0172514 | 2.19913 |
| RAB3B | 0.00100881 | 1.9242 |
| RAB3IP | 0.0272213 | 1.87452 |
| RACGAP1 | 0.0432753 | 1.99296 |
| RAD18 | 0.0359714 | 1.61243 |
| RAD51 | 0.00144215 | 2.8472 |
| RAI14 | 0.000648719 | 1.64497 |
| RASAL1 | 0.0397733 | 1.90307 |
| RASAL2 | 0.0453258 | 1.73317 |
| RBL1 | 0.0403488 | 1.71538 |
| REEP1 | 0.0348705 | 1.66057 |
| REEP4 | 0.0137681 | 1.54727 |
| RFC2 | 0.00652339 | 1.85226 |
| RFC3 | 0.00133801 | 3.09934 |
| RFC4 | 0.0471821 | 1.66693 |
| RFC5 | 0.0285894 | 1.58844 |
| RGMB | 0.00812537 | 1.71161 |
| RGNEF | 0.0106744 | 1.54312 |
| RHBG | 0.0172536 | 10.2044 |
| RNASEH1 | 0.00118493 | 1.62431 |
| RNASEH2A | 0.0461202 | 1.61834 |
| RP11-631M21.2 | 0.000815088 | 2.19128 |
| RP6-213H19.1 | 0.0433633 | 1.50907 |
| RRP15 | 0.0236168 | 1.56386 |
| RRS1 | 0.0442757 | 1.55211 |
| SAMD4A | 0.00361931 | 1.57355 |
| SDF2L1 | 0.0185587 | 1.61172 |
| SEMA7A | 0.0135539 | 1.85401 |
| SERPINB9 | 0.0429189 | 1.74317 |
| SERPINE1 | 0.0195248 | 2.14883 |
| SFRS2 | 0.00516241 | 1.60224 |
| SFRS3 | 0.00897117 | 1.64037 |
| SFRS7 | 0.00511139 | 1.93955 |
| SH2D5 | 0.00682358 | 1.6895 |
| SH3D20 | 0.0257641 | 2.44151 |
| SHISA3 | 0.0252495 | 2.03938 |
| SIPA1L3 | 0.00495103 | 2.50235 |
| SLC16A3 | 0.000123416 | 1.85549 |
| SLC16A7 | 0.0224313 | 1.66472 |
| SLC17A9 | 0.000487903 | 2.44726 |
| SLC20A2 | 0.00421458 | 1.69988 |
| SLC25A22 | 0.00419286 | 1.66757 |
| SLC25A4 | 0.019519 | 1.59931 |
| SLC2A3 | 0.00611322 | 2.17307 |
| SLC38A1 | 0.0198162 | 1.62009 |
| SLC45A4 | 0.00135059 | 1.71703 |
| SLC7A1 | 0.0024201 | 2.3587 |
| SLC7A5 | 0.0063327 | 2.50018 |
| SMC2 | 0.00700466 | 1.56097 |
| SMC4 | 0.0425135 | 1.71585 |
| SNAR-A3 | 0.00147711 | 2.95034 |
| SNRPA1 | 0.00370797 | 1.6928 |
| SPAG5 | 0.00573074 | 2.35519 |
| SPCS3 | 0.0114513 | 1.89835 |
| SPOCD1 | 0.00229441 | 3.00299 |
| SPRED1 | 0.0371546 | 1.54089 |
| SRM | 0.00110966 | 1.53056 |
| STAC | 0.0156102 | 1.93657 |
| STAMBPL1 | 0.00490136 | 1.57093 |
| STBD1 | 0.0459733 | 1.64556 |
| STEAP1 | 0.00123189 | 1.54119 |
| STIP1 | 0.00226165 | 1.61676 |
| STK17B | 0.00345971 | 1.52569 |
| STRN | 0.0401385 | 2.14506 |
| SULF1 | 0.00873385 | 1.55318 |
| SUV39H2 | 0.049487 | 1.52759 |
| TACC3 | 0.0219367 | 2.39267 |
| TAGLN | 0.0020595 | 2.36846 |
| TBC1D2 | 0.0229801 | 1.59851 |
| TCF19 | 0.00440783 | 2.25187 |
| TEAD4 | 0.00643645 | 1.61356 |
| THC2593778 | 0.00933236 | 2.37596 |
| THOC4 | 0.00754107 | 1.526 |
| THOP1 | 0.00302452 | 1.57303 |
| TIMELESS | 0.0331972 | 1.58239 |
| TIMM17A | 0.0115451 | 1.56084 |
| TIMM8A | 0.0235305 | 1.68671 |
| TIPIN | 0.0277199 | 1.5917 |
| TMCO7 | 0.0129978 | 1.59073 |
| TMEM22 | 0.0407767 | 1.84448 |
| TMEM99 | 0.0191929 | 1.54011 |
| TNFRSF11B | 0.034583 | 1.51911 |
| TNFRSF12A | 0.000246553 | 2.09696 |
| TOMM34 | 0.00467134 | 1.54459 |
| TOP2A | 0.0267857 | 116.7 |
| TPM1 | 0.000817651 | 2.43472 |
| TPM4 | 0.000482746 | 1.54807 |
| TRAIP | 0.0283375 | 1.70982 |
| TRAV20 | 0.0283795 | 1.5613 |
| TRMT6 | 0.0331748 | 1.64325 |
| TRPC4 | 0.00250818 | 1.54722 |
| TSR1 | 0.00229199 | 1.53789 |
| TSSC1 | 0.000766802 | 1.50523 |
| TTF2 | 0.00109972 | 1.95605 |
| TTLL8 | 1.47E-05 | 1.59477 |
| TUBA1A | 0.000189693 | 2.0263 |
| TUBA1B | 0.000307532 | 2.55966 |
| TUBA1C | 0.00275773 | 2.28727 |
| TUBB | 0.000128623 | 1.58247 |
| TUBB2A | 0.00171098 | 1.92259 |
| TUBB2C | 0.00153433 | 2.18605 |
| TUBB3 | 0.0219865 | 1.53965 |
| TUBB6 | 0.00331305 | 2.33181 |
| TUBG1 | 0.0029748 | 1.65645 |
| UAP1 | 0.0175324 | 2.02468 |
| UBE2C | 0.000747885 | 3.00806 |
| UBE2S | 0.029568 | 1.71448 |
| UBE2T | 0.0059958 | 2.26357 |
| UCK2 | 0.00238769 | 1.7149 |
| UHRF1 | 0.0127624 | 2.94356 |
| UNC5A | 0.0316455 | 1.86466 |
| URB1 | 0.0110665 | 2.13122 |
| USP46 | 0.0182436 | 2.27864 |
| UTP15 | 0.0273911 | 1.54478 |
| UXS1 | 0.0118151 | 1.62919 |
| VASP | 0.019953 | 1.73135 |
| VKORC1L1 | 0.00028225 | 1.56896 |
| VPS37A | 0.0424994 | 6.12918 |
| VRK1 | 0.0327379 | 2.00335 |
| WASH3P | 0.0136968 | 1.55316 |
| WDHD1 | 0.0197321 | 1.93152 |
| WDR1 | 0.00481682 | 1.87615 |
| WDR62 | 0.0367843 | 1.97946 |
| WFDC1 | 0.0112207 | 3.46838 |
| WNT5B | 0.00298566 | 1.84357 |
| WSB2 | 0.00411354 | 1.74892 |
| YIF1B | 0.0287723 | 1.61378 |
| ZDHHC13 | 0.0125196 | 1.75483 |
| ZNF215 | 0.0190651 | 1.89346 |
| ZNF225 | 0.0216354 | 1.87138 |
| ZNF430 | 0.0295005 | 1.55677 |
| ZNF595 | 0.00931538 | 1.51648 |
| ZNF649 | 0.0147774 | 3.1283 |
| ZNF710 | 0.0378279 | 3.06219 |
| ZWILCH | 0.019438 | 1.70371 |
| ZWINT | 0.00433806 | 2.75434 |

**Additional Table 2: Down-regulated mRNAs in healthy ASM cells after stimulation with FCS.**

| **Gene Symbol** | ***P* value** | **Fold-Change** |
| --- | --- | --- |
| ABCA1 | 0.00697862 | -2.01802 |
| ABCA7 | 0.00525003 | -2.29264 |
| ABCC6 | 0.0046221 | -1.7338 |
| ABHD14A | 0.00075711 | -1.53863 |
| ABI3BP | 0.0428495 | -1.83925 |
| ABTB1 | 0.015233 | -1.63173 |
| ACBD6 | 0.001466 | -1.7306 |
| ACCN1 | 0.0122147 | -1.93931 |
| ACER1 | 0.00026845 | -1.76091 |
| ACTRT2 | 0.0121919 | -2.38655 |
| ADAM8 | 0.0301276 | -1.86849 |
| ADAMTS10 | 0.0283683 | -1.86355 |
| AF143325 | 0.00337029 | -2.78789 |
| AGPAT4 | 0.0007566 | -1.7031 |
| AHCYL2 | 0.00465628 | -1.52966 |
| AK022088 | 0.00249777 | -1.9298 |
| AK027069 | 0.0259371 | -1.92735 |
| AK056419 | 0.0493965 | -1.53281 |
| AK096443 | 0.00466235 | -2.474 |
| AK123300 | 0.00197179 | -2.02984 |
| AK123797 | 0.00519586 | -3.03104 |
| AK124041 | 0.00355306 | -1.7933 |
| AK127966 | 0.0124415 | -1.67888 |
| AK309505 | 0.0105135 | -1.86298 |
| ALDH1A3 | 0.00523657 | -2.81955 |
| ALDH3A2 | 0.0156318 | -1.5651 |
| ALS2CL | 0.0421984 | -1.9487 |
| AMOT | 0.0327397 | -2.08699 |
| ANKDD1A | 0.0415391 | -1.68796 |
| ANKRD13C | 0.0273003 | -1.51539 |
| ANKRD33B | 0.041914 | -1.62934 |
| AOC2 | 0.0292134 | -2.92473 |
| APOC1 | 0.00584438 | -1.74546 |
| ARHGAP28 | 0.00969408 | -1.62347 |
| ARHGAP33 | 0.00054096 | -1.79879 |
| ARID3C | 0.0179544 | -2.03276 |
| ARL3 | 0.00042055 | -1.9921 |
| ARMCX4 | 0.014307 | -2.8891 |
| ARRB1 | 0.00591499 | -2.24769 |
| ASGR1 | 0.00325688 | -1.66822 |
| ASPRV1 | 0.04287 | -2.10317 |
| ATP6V1G2 | 0.0132952 | -1.86315 |
| AX747372 | 0.0475703 | -2.25486 |
| AX747437 | 0.00541857 | -2.58597 |
| AX747534 | 0.00145914 | -2.6965 |
| AX747676 | 0.00194282 | -3.45335 |
| AX747706 | 0.0323213 | -1.75876 |
| AX747836 | 0.00692359 | -3.37472 |
| BHLHE23 | 0.0025552 | -2.42498 |
| BM703906 | 0.00014491 | -1.73779 |
| BMP6 | 0.0299852 | -2.5272 |
| BST2 | 0.00097169 | -2.11198 |
| BTG1 | 0.0165385 | -1.56716 |
| BTG2 | 0.00017956 | -1.70859 |
| BTN3A1 | 0.00191576 | -1.6501 |
| BTN3A2 | 0.00373209 | -1.54639 |
| BX092453 | 0.0254932 | -1.53348 |
| BZRAP1 | 0.0452238 | -1.94784 |
| C10orf27 | 0.00491788 | -1.58778 |
| C10orf58 | 0.0326003 | -1.68517 |
| C11orf45 | 0.010093 | -1.92877 |
| C11orf52 | 0.00275074 | -2.14844 |
| C12orf53 | 0.00188867 | -1.9537 |
| C12orf60 | 0.00162816 | -2.67237 |
| C12orf72 | 0.0403867 | -1.52103 |
| C14orf132 | 0.00807592 | -1.60823 |
| C14orf139 | 0.00165645 | -1.75206 |
| C14orf162 | 0.0186933 | -1.98994 |
| C14orf93 | 0.00090318 | -1.77396 |
| C15orf51 | 0.00047418 | -1.83639 |
| C16orf78 | 0.0208598 | -2.65055 |
| C17orf100 | 0.0476427 | -1.77801 |
| C17orf108 | 0.018801 | -1.60705 |
| C17orf69 | 0.00101622 | -1.55078 |
| C1orf213 | 0.00066049 | -2.31608 |
| C1orf220 | 0.0445791 | -1.59497 |
| C1orf228 | 0.0235911 | -1.84057 |
| C1QL1 | 0.0304071 | -2.18534 |
| C20orf132 | 0.00504737 | -1.6353 |
| C20orf95 | 0.0403836 | -2.08899 |
| C22orf36 | 0.0398039 | -1.62203 |
| C2orf81 | 0.00162312 | -1.75671 |
| C3orf17 | 0.00308558 | -1.8265 |
| C3orf18 | 0.0147357 | -1.51743 |
| C5 | 0.0169664 | -1.54385 |
| C5orf39 | 0.00695142 | -1.52157 |
| C5orf4 | 0.00468082 | -2.22418 |
| C5orf41 | 0.00185897 | -1.7652 |
| C6orf122 | 0.00734411 | -2.41458 |
| C6orf147 | 0.0287088 | -2.13371 |
| C6orf154 | 0.00230847 | -1.70781 |
| C8G | 6.90E-05 | -1.54853 |
| C9orf106 | 0.0239609 | -1.99303 |
| C9orf139 | 0.00148394 | -3.15392 |
| C9orf169 | 0.00050604 | -1.57006 |
| C9orf24 | 0.00530183 | -2.0828 |
| C9orf7 | 0.00184493 | -1.58389 |
| C9orf9 | 0.00129577 | -1.50282 |
| C9orf96 | 0.00982553 | -2.21231 |
| C9orf98 | 0.0432906 | -1.80874 |
| CA12 | 0.0425827 | -1.76219 |
| CAPN5 | 0.016797 | -1.52674 |
| CARHSP1 | 0.00061125 | -1.58925 |
| CASZ1 | 0.0230826 | -1.54978 |
| CBLN3 | 0.0497352 | -1.95091 |
| CCDC102A | 0.00054828 | -1.59443 |
| CCDC102B | 0.0220139 | -2.06874 |
| CCDC103 | 0.00114918 | -2.11282 |
| CCDC154 | 0.0106494 | -2.02503 |
| CCDC21 | 0.0345365 | -2.29352 |
| CCDC23 | 0.0132711 | -1.67546 |
| CCDC30 | 0.0313069 | -2.73726 |
| CCDC88C | 0.00115919 | -1.61034 |
| CCKAR | 0.0210964 | -2.51162 |
| CCL11 | 0.00540288 | -5.07664 |
| CCL27 | 0.00379464 | -1.88682 |
| CCL7 | 0.038351 | -3.48602 |
| CCNG1 | 0.00073746 | -1.66384 |
| CD3D | 0.00497975 | -2.36269 |
| CD674797 | 0.00207141 | -1.78183 |
| CD82 | 0.00678088 | -1.72622 |
| CDK19 | 0.00068227 | -1.54769 |
| CDKN1C | 0.0170145 | -1.72462 |
| CDKN2B | 0.00180787 | -2.97134 |
| CECR4 | 0.00692698 | -1.67867 |
| CELA2B | 0.00777827 | -1.7659 |
| CELF2 | 0.0136844 | -1.52529 |
| CENPVL1 | 0.00929415 | -2.09744 |
| CEP68 | 0.0290225 | -1.79535 |
| CFB | 0.0453557 | -2.01426 |
| CH25H | 0.0310899 | -1.93761 |
| CHADL | 0.0336368 | -1.65503 |
| CHRD | 0.00812107 | -1.71295 |
| CILP | 0.0381664 | -3.31949 |
| CLCNKB | 0.00429945 | -1.87913 |
| CLDN15 | 0.0444003 | -1.57161 |
| CLEC12B | 0.0380597 | -1.66327 |
| CLEC3B | 6.65E-05 | -3.83958 |
| CLIC2 | 0.0316869 | -1.54762 |
| CMYA5 | 0.0319154 | -2.27659 |
| COL14A1 | 0.0197526 | -1.55844 |
| COL24A1 | 0.00073236 | -1.75158 |
| COL4A5 | 0.009473 | -1.91641 |
| COL9A3 | 0.00278875 | -1.70035 |
| CPAMD8 | 0.015032 | -2.10484 |
| CPT1B | 0.0265321 | -1.77996 |
| CPZ | 0.0191398 | -1.5251 |
| CR607250 | 0.00568383 | -1.684 |
| CR620599 | 0.0182817 | -1.54383 |
| CR739597 | 0.01195 | -1.9101 |
| CRABP2 | 0.00522919 | -1.82494 |
| CRTAP | 0.0140477 | -1.70593 |
| CRTC1 | 0.00804747 | -2.00269 |
| CRYBG3 | 0.0216741 | -1.60588 |
| CTSF | 0.0040411 | -1.6319 |
| CXCL1 | 0.0211651 | -5.06404 |
| CXCL12 | 0.0344235 | -2.06345 |
| CXCL2 | 0.0476977 | -2.70227 |
| CXorf42 | 0.00637727 | -2.48504 |
| CYS1 | 0.0115155 | -1.55187 |
| DA142060 | 0.0330543 | -2.1804 |
| DB074148 | 0.0201158 | -1.82322 |
| DB462629 | 0.0120879 | -2.04781 |
| DBT | 0.00168652 | -1.52261 |
| DCAF5 | 0.00105449 | -1.59693 |
| DCHS1 | 0.00628314 | -1.76835 |
| DCST1 | 0.00249387 | -2.76621 |
| DDB2 | 0.00028376 | -1.81671 |
| DERL3 | 0.0133346 | -1.51821 |
| DLG4 | 0.0283211 | -1.50601 |
| DNAH1 | 0.0328267 | -2.10634 |
| DNALI1 | 0.00716912 | -2.23669 |
| DNASE1L2 | 0.0435026 | -2.48599 |
| DNM3 | 0.0024522 | -1.86314 |
| DOCK4 | 0.00392464 | -1.81231 |
| DPT | 0.00022931 | -2.17034 |
| DQ895628 | 0.00135697 | -2.62086 |
| EBF1 | 0.00183354 | -1.98884 |
| EFCAB7 | 0.00525254 | -1.59661 |
| EGR2 | 0.022 | -2.85361 |
| ENST00000217224 | 0.00967839 | -1.89812 |
| ENST00000299234 | 0.00199467 | -1.57617 |
| ENST00000320486 | 0.0133566 | -1.71104 |
| ENST00000322069 | 0.0296774 | -1.5799 |
| ENST00000324945 | 0.0177289 | -1.95988 |
| ENST00000331346 | 0.0400823 | -1.68969 |
| ENST00000331733 | 0.0120203 | -1.6265 |
| ENST00000339692 | 0.0272295 | -1.83005 |
| ENST00000340798 | 0.0268956 | -1.94792 |
| ENST00000369159 | 0.00020701 | -1.56743 |
| ENST00000372591 | 0.00179265 | -2.72904 |
| ENST00000376536 | 0.00018462 | -1.52355 |
| ENST00000376964 | 0.0256955 | -2.71169 |
| ENST00000380242 | 0.0206066 | -1.68984 |
| ENST00000390268 | 0.00961821 | -1.95954 |
| ENST00000390431 | 0.0216309 | -1.86328 |
| ENST00000396468 | 0.0388438 | -1.89207 |
| ENST00000398617 | 0.0069792 | -2.285 |
| ENST00000399541 | 0.0277977 | -1.57262 |
| ENST00000399945 | 0.0149545 | -2.05122 |
| ENST00000403389 | 0.0019834 | -1.92324 |
| ENST00000409310 | 0.040627 | -1.94976 |
| ENST00000409554 | 0.00167371 | -3.21689 |
| ENST00000422553 | 0.0272331 | -1.68602 |
| ENST00000428332 | 0.0115752 | -1.55903 |
| ENST00000429183 | 0.0180525 | -2.63082 |
| ENST00000443866 | 0.0457578 | -1.91513 |
| EPHB3 | 0.00288621 | -1.60326 |
| EPHX2 | 0.0341255 | -1.60476 |
| ERMAP | 0.0105329 | -1.78461 |
| ESPNL | 0.0039744 | -1.89723 |
| ESRRG | 0.0114511 | -2.23679 |
| EU154352 | 0.0332319 | -1.97494 |
| F10 | 0.00364789 | -1.71244 |
| FAM117B | 0.00236341 | -1.95465 |
| FAM13A | 0.00105064 | -1.52487 |
| FAM198A | 0.0260313 | -2.20093 |
| FAM20A | 0.010097 | -2.91265 |
| FAM38B | 0.0183081 | -1.58043 |
| FAM48B1 | 0.0139611 | -2.09454 |
| FAM57B | 0.0414802 | -1.52673 |
| FAM71F1 | 0.0357019 | -1.67751 |
| FBXL14 | 0.0331503 | -2.41013 |
| FBXO15 | 0.0328355 | -1.84646 |
| FBXO2 | 0.00080488 | -1.90997 |
| FBXO22OS | 0.0457567 | -1.57657 |
| FBXO24 | 0.00449817 | -1.62964 |
| FDXR | 0.00145632 | -1.76945 |
| FGD2 | 0.0411921 | -1.87012 |
| FGF10 | 0.0266331 | -1.58225 |
| FGF18 | 0.0410238 | -3.02169 |
| FLJ13197 | 0.0138774 | -2.29402 |
| FLJ31713 | 0.0224083 | -1.54633 |
| FLJ41649 | 0.0424734 | -1.79425 |
| FLJ44790 | 0.0296422 | -1.92054 |
| FLJ45445 | 0.025556 | -1.67566 |
| FMO5 | 0.0202892 | -1.56914 |
| FOLR1 | 0.00018692 | -2.05172 |
| FOXD4 | 0.00190232 | -3.47364 |
| FOXO6 | 0.00619086 | -2.20966 |
| FOXP2 | 0.00435789 | -1.78458 |
| FRAT1 | 0.026951 | -1.53198 |
| FSTL5 | 0.00909879 | -2.99265 |
| GAS1 | 6.02E-05 | -2.14084 |
| GATAD2B | 0.00713309 | -1.64161 |
| GFRA1 | 0.00046796 | -1.64226 |
| GGA2 | 0.00603644 | -1.56331 |
| GLI3 | 0.00493657 | -1.6098 |
| GLIS1 | 0.0264764 | -1.58953 |
| GPR109A | 0.0347042 | -1.50782 |
| GPR162 | 0.00451163 | -1.7352 |
| GPR20 | 0.0191366 | -1.59713 |
| GPR62 | 0.0162333 | -1.92832 |
| GRK4 | 0.0170569 | -1.51418 |
| GTF2IRD2 | 0.03209 | -1.60104 |
| GXYLT2 | 0.00066286 | -1.74459 |
| H19 | 0.0455882 | -6.98269 |
| HBA2 | 0.00803206 | -3.13049 |
| HCFC1R1 | 0.0187693 | -1.62769 |
| HDAC5 | 0.010239 | -1.72172 |
| HECA | 0.00468956 | -1.53453 |
| HEXIM2 | 0.00303006 | -1.77702 |
| HHATL | 0.00471783 | -1.99539 |
| HHLA2 | 0.0159444 | -1.66531 |
| HIP1R | 0.0151118 | -1.54101 |
| HSD17B6 | 0.0222164 | -2.01038 |
| HSD17B8 | 0.0140345 | -1.5763 |
| HSPA12B | 0.00163158 | -3.55519 |
| HSPB2 | 0.0125954 | -1.52504 |
| HSPB9 | 0.00458144 | -1.7123 |
| HTRA3 | 0.0247119 | -2.93974 |
| HVCN1 | 0.0164719 | -1.61039 |
| ICAM4 | 0.0005566 | -2.37395 |
| IFIT1 | 0.00164474 | -1.62618 |
| IFT140 | 0.0337115 | -1.75025 |
| IGDCC4 | 0.0032925 | -2.18598 |
| IGLL1 | 0.0356818 | -1.88242 |
| IL17RD | 0.0114468 | -1.64791 |
| IL4I1 | 0.00370299 | -3.22767 |
| INSR | 0.00144856 | -1.66462 |
| IQCD | 0.0259108 | -1.88789 |
| ISG20 | 0.00093856 | -2.38481 |
| ISLR | 0.00629613 | -1.60028 |
| ITGB4 | 0.00146948 | -2.2568 |
| ITIH4 | 0.00380577 | -2.23681 |
| ITIH5L | 0.0368887 | -1.51252 |
| ITPKC | 0.00322039 | -1.51603 |
| JAG1 | 0.00101446 | -1.67955 |
| JAKMIP3 | 0.0259157 | -2.03597 |
| KCNK4 | 0.0252145 | -2.00469 |
| KIAA1407 | 0.0171847 | -2.2112 |
| KIAA1683 | 0.00832953 | -1.9789 |
| KIAA2026 | 0.0264928 | -2.01752 |
| KIFC2 | 0.0426302 | -1.55722 |
| KLF4 | 0.00052814 | -2.00055 |
| KLHDC1 | 0.0442986 | -2.03571 |
| KLHDC9 | 0.0419066 | -1.78469 |
| KLHL24 | 0.0155853 | -1.79066 |
| KRT85 | 0.0181777 | -1.71564 |
| KRTAP2-1 | 0.00679702 | -1.73611 |
| KRTAP6-1 | 0.00413709 | -1.61449 |
| LAG3 | 0.016262 | -1.55713 |
| LAMB2L | 0.0095458 | -2.01829 |
| LAMB3 | 0.0179455 | -1.54675 |
| LCN12 | 0.0374029 | -1.97631 |
| LDB2 | 0.0155814 | -1.69921 |
| LENG9 | 0.0205002 | -1.52368 |
| LETMD1 | 0.00430415 | -1.62123 |
| LGI4 | 0.0109025 | -2.80567 |
| LIAS | 0.00847173 | -1.51242 |
| LILRA4 | 0.0219441 | -1.70924 |
| LIPN | 0.0124328 | -1.93656 |
| LOC100049716 | 0.00943752 | -1.56888 |
| LOC100128055 | 0.0349769 | -1.80597 |
| LOC100128155 | 0.0332283 | -1.81554 |
| LOC100128163 | 0.0469868 | -1.59505 |
| LOC100128320 | 0.0216957 | -1.7234 |
| LOC100128328 | 0.00057102 | -1.69526 |
| LOC100128398 | 0.00995442 | -1.88914 |
| LOC100128460 | 0.0106676 | -1.545 |
| LOC100128511 | 0.0148655 | -2.1244 |
| LOC100128909 | 0.0136927 | -3.01647 |
| LOC100128913 | 0.0302836 | -1.7844 |
| LOC100129035 | 0.0156117 | -1.91893 |
| LOC100129203 | 0.0253826 | -2.1577 |
| LOC100129269 | 0.0445059 | -1.96269 |
| LOC100129531 | 0.0359211 | -1.87734 |
| LOC100129550 | 0.00443723 | -1.58352 |
| LOC100130000 | 0.00058135 | -2.15337 |
| LOC100130111 | 0.00276904 | -1.8049 |
| LOC100130387 | 0.0227905 | -2.40141 |
| LOC100130453 | 0.0235435 | -1.75038 |
| LOC100130557 | 0.00618496 | -1.92351 |
| LOC100130800 | 0.00957426 | -1.66391 |
| LOC100131326 | 0.00923259 | -1.66554 |
| LOC100131342 | 0.036421 | -1.50788 |
| LOC100131355 | 0.00565705 | -1.70525 |
| LOC100131372 | 0.0262034 | -1.75575 |
| LOC100131830 | 0.00145728 | -1.51645 |
| LOC100132593 | 0.00982822 | -2.03553 |
| LOC100133001 | 0.0492663 | -1.62939 |
| LOC100134937 | 0.008973 | -1.6031 |
| LOC144571 | 0.00251852 | -1.82331 |
| LOC145945 | 0.0176511 | -2.22565 |
| LOC147646 | 0.0322007 | -1.96635 |
| LOC153546 | 0.00381535 | -1.52241 |
| LOC154860 | 0.0241093 | -1.54151 |
| LOC157562 | 0.0222838 | -1.77389 |
| LOC255480 | 0.0389134 | -1.78965 |
| LOC282997 | 0.00204892 | -1.62941 |
| LOC283392 | 0.0199356 | -1.60014 |
| LOC284263 | 0.0307267 | -1.75858 |
| LOC284276 | 0.00989207 | -1.81011 |
| LOC284408 | 0.00165328 | -1.99447 |
| LOC284900 | 0.0258379 | -1.51739 |
| LOC285141 | 0.0320039 | -1.50351 |
| LOC285147 | 0.00635723 | -2.70196 |
| LOC286161 | 0.00577979 | -1.58986 |
| LOC340357 | 0.0486625 | -1.86338 |
| LOC375010 | 0.00843783 | -1.5953 |
| LOC439911 | 0.0285936 | -1.99503 |
| LOC440104 | 0.0120035 | -1.90576 |
| LOC55908 | 0.00593429 | -2.6717 |
| LOC572558 | 0.0322892 | -2.28307 |
| LOC643699 | 0.00316989 | -2.28546 |
| LOC644173 | 0.0215081 | -1.53146 |
| LOC644525 | 0.011288 | -2.17486 |
| LOC645427 | 0.00804996 | -1.50863 |
| LOC645685 | 0.0290932 | -1.81493 |
| LOC678655 | 0.0134933 | -1.80326 |
| LOC727869 | 6.84E-05 | -2.44919 |
| LOC728163 | 0.0379407 | -1.50807 |
| LOC728705 | 0.0327362 | -1.54399 |
| LOC729013 | 0.00400269 | -1.70173 |
| LOC729199 | 0.0152863 | -2.62112 |
| LOC730102 | 0.0128413 | -1.51254 |
| LOC730235 | 0.0141815 | -1.58384 |
| LOC730651 | 0.0127135 | -1.77602 |
| LOC90110 | 0.00261475 | -1.5337 |
| LOC92973 | 0.00476721 | -2.41088 |
| LOH12CR2 | 0.0128989 | -1.54348 |
| LPAR6 | 0.012144 | -1.75964 |
| LRDD | 0.0100229 | -1.75178 |
| LRRC10 | 0.0133667 | -1.7157 |
| LRRC20 | 0.00190546 | -1.57617 |
| LRRC24 | 0.0082574 | -1.54 |
| LRRC27 | 0.00010708 | -1.76391 |
| LRRK2 | 0.00404663 | -1.89717 |
| LTBP4 | 0.00643656 | -1.59364 |
| LUM | 0.0154056 | -1.68684 |
| MAF | 0.0329326 | -1.64253 |
| MAN2A2 | 0.0322019 | -1.68821 |
| MAP2K6 | 0.0115569 | -1.77604 |
| MAPK10 | 0.0106283 | -1.63506 |
| MAPT | 0.00620602 | -2.47255 |
| MAST1 | 0.0025605 | -1.67026 |
| MCPH1 | 0.0171771 | -2.04762 |
| MDFI | 0.0157319 | -1.83616 |
| MED28 | 0.0199466 | -1.94019 |
| MEGF8 | 0.0286648 | -1.56185 |
| MEIG1 | 0.0193803 | -2.63048 |
| METTL7A | 0.00263136 | -1.9747 |
| MFAP4 | 0.00956891 | -1.58343 |
| MGC31957 | 0.0129225 | -1.61621 |
| MGC42157 | 0.038865 | -1.5075 |
| MGC5566 | 0.018925 | -3.00276 |
| MICALL2 | 0.018573 | -1.52798 |
| MMP23B | 0.00562863 | -1.96165 |
| MST1 | 0.0185667 | -1.50664 |
| MSX2P1 | 0.013784 | -1.75347 |
| MUC8 | 0.016403 | -2.27774 |
| MURC | 0.0350392 | -1.7504 |
| MUSTN1 | 0.0167156 | -2.91232 |
| MX2 | 0.0158867 | -1.96304 |
| MXD4 | 0.0035274 | -1.7148 |
| MXI1 | 0.00049594 | -1.6678 |
| MXRA5 | 0.0139811 | -2.07221 |
| MYL4 | 0.00284965 | -2.19305 |
| MYLIP | 0.0459912 | -1.92418 |
| MYO7B | 0.0215031 | -1.92928 |
| MYOM1 | 0.0210774 | -1.7951 |
| N4BP2L1 | 0.0318774 | -2.44421 |
| NAIP | 0.0330237 | -1.99151 |
| NBEA | 0.00611376 | -1.60651 |
| NCAPH | 0.0149019 | -1.51139 |
| NCRNA00106 | 0.0393343 | -1.59184 |
| NEDD4L | 0.00738368 | -1.56874 |
| NEK11 | 0.0235669 | -1.57873 |
| NFATC4 | 0.00199543 | -2.11411 |
| NFE2 | 0.00978393 | -2.42908 |
| NFKBIE | 0.00348657 | -1.55798 |
| NFKBIZ | 0.0100366 | -1.79679 |
| NHLRC4 | 0.0326244 | -1.55589 |
| NINL | 0.0198357 | -1.51634 |
| NIPAL2 | 0.00126838 | -1.73365 |
| NKX6-3 | 0.0196904 | -2.06921 |
| NNAT | 0.0346275 | -1.89862 |
| NOTCH3 | 0.0459731 | -1.76246 |
| NOXA1 | 0.0434108 | -1.76053 |
| NPHP1 | 0.0145253 | -1.59891 |
| NR1H3 | 0.00136844 | -1.50898 |
| NR6A1 | 0.0329603 | -1.66975 |
| NRN1L | 0.00136503 | -2.48116 |
| NTN1 | 0.0289739 | -2.0237 |
| NTNG2 | 0.00064662 | -1.53872 |
| NUDT13 | 0.0304724 | -1.62895 |
| NUMBL | 0.00078424 | -1.64874 |
| NXPH3 | 0.00458855 | -2.43988 |
| NYNRIN | 0.00938078 | -1.75262 |
| ODF3B | 0.0196357 | -1.54555 |
| OGN | 0.0121143 | -2.27392 |
| OLFML1 | 0.00663884 | -1.99569 |
| OLFML2A | 0.00041463 | -3.82847 |
| OR10J5 | 0.0164523 | -2.00088 |
| OR1K1 | 0.0237546 | -2.25083 |
| OR2AG2 | 0.00668147 | -2.04473 |
| OR51I2 | 0.0109357 | -1.67789 |
| OR6C74 | 0.0113665 | -2.69253 |
| OR6Y1 | 0.00371803 | -2.06077 |
| OSR2 | 0.009941 | -2.04294 |
| OVGP1 | 0.0399041 | -1.57316 |
| OXER1 | 0.0402824 | -1.74548 |
| PALM | 0.00175841 | -1.66815 |
| PAPPAS | 0.0429692 | -1.69269 |
| PAQR6 | 0.0360072 | -1.83245 |
| PAQR8 | 0.00599899 | -2.25638 |
| PATE3 | 0.00482685 | -2.41531 |
| PATZ1 | 0.0403087 | -1.54481 |
| PAX8 | 0.00467385 | -1.74612 |
| PBX1 | 0.0268093 | -1.5622 |
| PCDHB14 | 0.0243173 | -1.89357 |
| PCDHB8 | 0.0477968 | -1.94049 |
| PCNXL2 | 0.00079946 | -2.60963 |
| PCSK4 | 0.0192554 | -1.51335 |
| PCSK9 | 0.0193312 | -2.1655 |
| PDCD4 | 0.0135174 | -1.84912 |
| PDGFRA | 0.0167617 | -1.56683 |
| PDGFRB | 0.00464275 | -1.56222 |
| PDK2 | 0.0190484 | -1.65348 |
| PGA3 | 0.00447282 | -2.29092 |
| PHF21A | 0.025883 | -1.5007 |
| PHF7 | 0.00337171 | -3.05325 |
| PIGZ | 0.0310595 | -1.53358 |
| PIK3C2B | 0.0274308 | -2.42403 |
| PIK3IP1 | 0.046845 | -1.78011 |
| PION | 0.00770339 | -1.80057 |
| PLA2R1 | 0.0403783 | -1.7811 |
| PLCD4 | 0.00125027 | -1.82204 |
| PLCL1 | 0.00541797 | -1.60909 |
| PLD2 | 0.00684566 | -1.50334 |
| PLK1S1 | 0.0130018 | -1.74677 |
| PLSCR4 | 0.00060408 | -1.51048 |
| PLTP | 0.00431702 | -1.85106 |
| PLXDC1 | 0.0011158 | -1.80241 |
| PMPCB | 0.00187659 | -1.57292 |
| PNPLA7 | 0.0332239 | -1.85728 |
| PNRC1 | 0.00278948 | -1.94228 |
| POLL | 0.00419854 | -1.50041 |
| PPAP2A | 0.0317407 | -1.86526 |
| PPAP2B | 0.0295061 | -1.56615 |
| PPL | 0.0180331 | -2.86177 |
| PPP1R1B | 0.046577 | -3.44713 |
| PPP1R3E | 0.0125434 | -1.85055 |
| PRELP | 0.0263994 | -2.53789 |
| PRICKLE1 | 0.0416314 | -1.53853 |
| PROCA1 | 0.0259899 | -1.63369 |
| PRRG3 | 0.0147154 | -1.90017 |
| PRRT1 | 0.0165626 | -1.91649 |
| PRRT2 | 0.0305519 | -2.67432 |
| PRSS36 | 0.0280329 | -1.52634 |
| PSPN | 0.0204953 | -1.60944 |
| PTBP2 | 0.0444032 | -1.52553 |
| PTGER4 | 0.0150857 | -1.57485 |
| PTGFRN | 0.0410646 | -1.80274 |
| PTPRD | 0.0177797 | -1.93606 |
| PTPRS | 0.00030207 | -1.62693 |
| PTPRU | 0.00756828 | -1.67952 |
| PURG | 0.0097724 | -1.67874 |
| QPRT | 0.00392315 | -1.70131 |
| RAB26 | 0.00393801 | -4.23985 |
| RAB7B | 0.0302493 | -1.71049 |
| RADIL | 0.0246198 | -1.67778 |
| RASA4 | 0.00915453 | -1.61323 |
| RASAL3 | 0.00066949 | -1.61838 |
| RASGEF1A | 0.0070235 | -2.49194 |
| RASL12 | 0.0351017 | -1.84584 |
| RASSF2 | 0.00576919 | -1.7715 |
| RASSF4 | 0.0161093 | -1.66753 |
| RASSF5 | 0.0443135 | -1.50768 |
| RAX2 | 0.0192167 | -2.20841 |
| RDH5 | 0.0384369 | -1.88394 |
| RELB | 0.00665261 | -1.96623 |
| RFTN2 | 0.0102231 | -1.55818 |
| RGL2 | 0.0158411 | -1.56403 |
| RGS22 | 0.0412112 | -1.78905 |
| RN5-8S1 | 0.00023347 | -1.69384 |
| RND2 | 0.0149727 | -3.21913 |
| RNF112 | 0.00655606 | -1.58857 |
| RNF151 | 0.0146506 | -1.60523 |
| ROM1 | 0.00469306 | -1.59117 |
| RPL3L | 0.0297286 | -1.51633 |
| RTN4RL1 | 0.00122609 | -1.98946 |
| RUNX1T1 | 0.0226093 | -1.54944 |
| SALL2 | 0.0154022 | -1.66752 |
| SATB1 | 0.00890588 | -1.5226 |
| SCAI | 0.00275677 | -1.60129 |
| SCARNA10 | 0.0153285 | -2.24079 |
| SCN2A | 0.0414266 | -1.61009 |
| SCN2B | 0.00501986 | -1.57636 |
| SCN7A | 0.00182735 | -3.13378 |
| SDCBP2 | 0.0399964 | -1.80957 |
| SELENBP1 | 0.00098318 | -1.99305 |
| SEMA3B | 0.00106982 | -2.15958 |
| SEMA3F | 0.0150743 | -1.82538 |
| SENP6 | 0.0096981 | -1.90529 |
| SEPP1 | 0.0177354 | -1.85901 |
| SESN3 | 0.0226974 | -1.91744 |
| SETBP1 | 0.0244478 | -1.56971 |
| SH3D19 | 0.0141843 | -1.76305 |
| SHANK3 | 0.00381411 | -2.15698 |
| SHBG | 0.00490741 | -2.36769 |
| SHC2 | 0.00359649 | -1.73242 |
| SIRT4 | 0.0355289 | -1.96565 |
| SIX5 | 0.0031781 | -1.76736 |
| SLC13A3 | 0.00103742 | -1.93889 |
| SLC25A23 | 0.00464343 | -1.55857 |
| SLC27A3 | 0.0401412 | -1.52968 |
| SLC44A1 | 0.00774372 | -1.63529 |
| SLC5A3 | 0.00418181 | -1.86105 |
| SLC6A16 | 0.00513484 | -2.48363 |
| SLC6A17 | 0.0327734 | -1.65436 |
| SLC7A8 | 0.0259705 | -1.55417 |
| SLC9A9 | 0.00478687 | -1.98908 |
| SMAD1 | 0.00893418 | -1.61458 |
| SMAD5OS | 0.0270401 | -2.07154 |
| SMOX | 0.0381185 | -1.62942 |
| SNAI2 | 0.0429141 | -1.50629 |
| SNHG7 | 0.00583094 | -1.554 |
| SNORA12 | 0.00105629 | -2.03437 |
| SNX1 | 0.00552262 | -1.57588 |
| SNX21 | 0.00286476 | -1.7138 |
| SOD3 | 0.00919768 | -1.709 |
| SPAG8 | 0.0474904 | -1.77878 |
| SPATA18 | 0.00106717 | -1.86113 |
| SPDYC | 0.0128553 | -2.47954 |
| SPTBN4 | 0.00796458 | -1.79902 |
| SSC5D | 0.0055637 | -1.90761 |
| ST8SIA1 | 0.00354432 | -2.0196 |
| STARD10 | 0.0144156 | -1.56945 |
| STON1 | 0.00657075 | -1.52343 |
| SULF2 | 0.00580506 | -1.98592 |
| SYNE2 | 0.0346488 | -1.67846 |
| TAC3 | 0.0362336 | -2.07286 |
| TARSL2 | 0.0227437 | -2.13865 |
| TBC1D10C | 0.0349813 | -1.73994 |
| TBC1D3B | 0.00567618 | -2.28755 |
| TCEA3 | 0.0204575 | -1.55808 |
| TCF4 | 0.00118427 | -1.51024 |
| TCF7L2 | 0.0312351 | -1.61667 |
| TCP11L2 | 0.00612874 | -1.65261 |
| THAP9 | 0.00651486 | -2.45628 |
| THBS4 | 0.0067613 | -1.81641 |
| THC2499708 | 0.0147853 | -2.39617 |
| THC2525522 | 0.0386956 | -1.81 |
| THC2533491 | 0.00145399 | -2.09313 |
| THC2544427 | 0.0316521 | -1.61439 |
| THC2556327 | 0.0273699 | -2.16831 |
| THC2564152 | 0.0403712 | -1.57836 |
| THC2614422 | 0.00757635 | -1.84592 |
| THC2655610 | 0.0171335 | -1.71949 |
| THC2728054 | 0.0415206 | -1.82085 |
| THC2764040 | 0.0323968 | -1.50152 |
| THC2775681 | 0.00566768 | -1.90485 |
| THC2786127 | 0.00290181 | -1.93806 |
| TLE2 | 0.00472166 | -1.6552 |
| TLE3 | 0.0350136 | -1.73379 |
| TLR2 | 0.0162706 | -2.01669 |
| TMEFF2 | 0.010788 | -2.1762 |
| TMEM130 | 0.00934813 | -1.92208 |
| TMEM132A | 0.00522125 | -1.53385 |
| TMEM151A | 0.0465898 | -1.84428 |
| TMEM151B | 0.00287779 | -4.56772 |
| TMEM184A | 0.0396513 | -1.75271 |
| TMEM190 | 0.00861938 | -2.66545 |
| TMEM229B | 0.0343936 | -1.77812 |
| TMEM35 | 0.026389 | -2.15406 |
| TMEM88 | 0.00143375 | -2.99037 |
| TMEM91 | 0.00189162 | -1.82376 |
| TMOD4 | 0.0204793 | -2.43409 |
| TNFRSF10C | 0.0165617 | -1.76877 |
| TNFRSF21 | 0.0242483 | -1.56772 |
| TNFRSF25 | 0.0487377 | -1.78364 |
| TNFSF10 | 0.00415803 | -1.9946 |
| TNFSF9 | 0.0280899 | -1.60852 |
| TNNC1 | 0.00266188 | -2.30281 |
| TNNC2 | 0.00145455 | -2.14191 |
| TNXB | 0.0179517 | -1.88012 |
| TP53INP1 | 0.00096149 | -1.79912 |
| TRIM38 | 0.00114355 | -1.58251 |
| TRIM47 | 0.00507192 | -1.77693 |
| TRIM54 | 0.0386449 | -1.52335 |
| TSKS | 0.0139153 | -2.91699 |
| TSPYL2 | 0.00203708 | -1.86622 |
| TSSK3 | 0.02209 | -1.75718 |
| TXNIP | 0.00035512 | -1.5148 |
| UBE2DNL | 0.0246351 | -1.70774 |
| UBN2 | 0.00655406 | -1.57533 |
| UCA1 | 0.00180579 | -1.86655 |
| UCN | 0.0054577 | -1.93092 |
| USP25 | 0.0334093 | -1.76628 |
| USP9X | 0.00668435 | -1.53449 |
| VILL | 0.00056507 | -1.94789 |
| VPS37D | 0.00518105 | -1.64045 |
| VWCE | 0.00101333 | -2.25964 |
| WBP1 | 0.00306692 | -1.55474 |
| WBSCR26 | 0.0166157 | -1.73596 |
| WDFY4 | 0.0263097 | -1.79242 |
| WDR63 | 0.00301121 | -1.58709 |
| WNT10B | 0.0329864 | -1.66644 |
| X58402 | 0.0290045 | -1.61202 |
| ZBTB46 | 0.0018129 | -2.07785 |
| ZC3H6 | 0.00581112 | -1.96345 |
| ZCWPW1 | 0.0007718 | -1.65822 |
| ZDHHC11 | 0.0404977 | -1.50119 |
| ZG16B | 0.0442191 | -1.9182 |
| ZMIZ2 | 0.00174845 | -1.53707 |
| ZNF177 | 0.0306716 | -1.85306 |
| ZNF204P | 0.0338678 | -2.51086 |
| ZNF345 | 0.0386646 | -1.52122 |
| ZNF354A | 0.0380468 | -1.67136 |
| ZNF446 | 0.00446972 | -1.51397 |
| ZNF524 | 0.00693352 | -1.61559 |
| ZNF564 | 0.0243093 | -1.65525 |
| ZNF575 | 0.00477276 | -1.59376 |
| ZNF581 | 0.0235138 | -1.77308 |
| ZNF606 | 0.00654946 | -1.89861 |
| ZNF608 | 0.018116 | -1.83093 |
| ZNF821 | 0.0277166 | -1.56467 |
| ZNRF1 | 0.00515262 | -1.54706 |
| ZSCAN1 | 0.00751506 | -2.65388 |

**Additional Table 3: Up-regulated mRNAs in healthy ASM cells after stimulation with Dex + FCS.**

| **Gene Symbol** | ***P* value** | **Fold-Change** |
| --- | --- | --- |
| AB385485 | 0.00958571 | 2.40924 |
| ABCA6 | 2.07E-05 | 1.96318 |
| ABCC3 | 0.000411824 | 2.39447 |
| ABCE1 | 0.00366345 | 1.69815 |
| ABHD15 | 2.44E-05 | 1.92677 |
| ABL2 | 0.00594073 | 2.0323 |
| ABT1 | 0.0482669 | 1.59006 |
| ABTB2 | 0.0179342 | 1.54118 |
| ACOT1 | 0.000167133 | 1.71078 |
| ACOT4 | 0.0215577 | 2.31647 |
| ACOT7 | 0.00115225 | 1.68233 |
| ACPT | 0.0266227 | 2.11652 |
| ACSS1 | 0.0383963 | 1.66843 |
| ACTA2 | 0.000546674 | 4.43551 |
| ACTB | 0.0231448 | 1.52819 |
| ACTBL2 | 4.94E-05 | 3.59524 |
| ACTG1 | 0.0152182 | 1.80901 |
| ACTG2 | 0.000238692 | 8.53801 |
| ACTN1 | 0.000680909 | 2.31715 |
| ACTN3 | 0.00334731 | 2.60909 |
| ACTR2 | 0.00710896 | 1.73971 |
| ACTR3 | 0.000864125 | 1.89162 |
| ADAM19 | 0.00235526 | 1.93186 |
| ADAM28 | 0.0010882 | 2.39429 |
| ADAMTS1 | 0.000285696 | 4.86296 |
| ADAMTS4 | 0.0228085 | 1.80917 |
| ADAMTS9 | 0.00145725 | 2.82468 |
| ADAP1 | 2.95E-05 | 2.73053 |
| ADARB1 | 0.000524783 | 3.85605 |
| ADAT1 | 0.0118013 | 1.80264 |
| ADM | 0.0378363 | 1.94757 |
| ADPGK | 0.0209916 | 1.72667 |
| ADPRH | 0.00086141 | 1.64759 |
| ADRA1B | 0.00804742 | 11.63 |
| ADRB2 | 0.0111093 | 3.05407 |
| AFAP1L1 | 0.000529331 | 2.42435 |
| AGK | 0.0139454 | 1.7286 |
| AGMAT | 0.0464042 | 1.88872 |
| AGPAT9 | 0.0255631 | 1.94718 |
| AIDA | 0.0416476 | 1.56308 |
| AK123826 | 0.0309655 | 2.39148 |
| AK311627 | 0.00648582 | 1.60243 |
| AKAP2 | 0.00653993 | 3.20101 |
| AKAP7 | 0.0432358 | 1.53373 |
| ALCAM | 0.00189678 | 3.03926 |
| ALDH1B1 | 0.00274853 | 2.09332 |
| ALDH4A1 | 0.00120577 | 1.53799 |
| ALG2 | 0.000458746 | 1.56757 |
| AMD1 | 0.00285129 | 1.7452 |
| AMOTL2 | 0.0391252 | 1.84239 |
| AMPH | 0.0340696 | 1.5053 |
| ANKLE2 | 0.014109 | 1.57918 |
| ANKRD27 | 0.037249 | 1.50254 |
| ANKRD36BP1 | 0.000316967 | 2.10511 |
| ANO3 | 0.00171058 | 3.34793 |
| ANPEP | 0.00120912 | 1.95652 |
| ANXA1 | 0.000593985 | 1.76672 |
| ANXA2 | 0.000152869 | 1.98842 |
| ANXA2P1 | 0.00390162 | 2.39944 |
| ANXA2P3 | 0.00142802 | 2.31248 |
| ANXA6 | 0.00879453 | 1.67592 |
| AOX1 | 0.00024594 | 3.42006 |
| AP1M1 | 0.0116689 | 1.83664 |
| AP4S1 | 0.0256814 | 1.67389 |
| APBB2 | 3.45E-05 | 1.83728 |
| APOOL | 0.0253003 | 1.60458 |
| ARF6 | 0.00365441 | 1.98234 |
| ARG2 | 0.0320446 | 1.97039 |
| ARHGAP29 | 0.00230693 | 2.52509 |
| ARHGDIB | 0.00159286 | 4.87621 |
| ARMC8 | 2.47E-05 | 2.76154 |
| ARNT2 | 0.0150995 | 1.76472 |
| ARPC4 | 0.00203968 | 1.75518 |
| ARPC5 | 0.00212381 | 2.05487 |
| ARPM1 | 0.0247442 | 1.57868 |
| ARRDC2 | 0.00340837 | 1.91748 |
| ARSG | 0.0460203 | 1.85117 |
| ARSJ | 0.0156947 | 2.07785 |
| ARSK | 0.000101939 | 1.89973 |
| ASPN | 0.0384997 | 3.1773 |
| ASTN2 | 0.00137851 | 2.25553 |
| ATOH8 | 0.000233629 | 2.16941 |
| ATP10A | 0.00257731 | 3.24521 |
| ATP13A2 | 0.00407353 | 1.51483 |
| ATP13A3 | 0.0333815 | 1.50518 |
| ATP1A4 | 0.0203891 | 1.59385 |
| ATP1B1 | 0.00603952 | 1.85699 |
| ATP2A2 | 0.000232322 | 1.63714 |
| ATP5G1 | 0.00355027 | 1.61715 |
| ATP6V1B2 | 0.0188754 | 1.95525 |
| ATP6V1C2 | 0.0158022 | 2.08612 |
| AVEN | 0.00312669 | 1.53322 |
| AX747335 | 0.000760454 | 4.04412 |
| AX747582 | 0.0361158 | 1.7374 |
| AX748282 | 0.0197788 | 1.94973 |
| AY358103 | 0.0445426 | 1.7632 |
| AY358253 | 0.000392495 | 1.76492 |
| B3GALT2 | 0.000806877 | 7.85671 |
| B3GNT5 | 0.0134795 | 2.30062 |
| B3GNTL1 | 0.0127107 | 1.50439 |
| B4GALT4 | 0.000425824 | 2.0318 |
| BAG3 | 0.00412093 | 2.29295 |
| BAIAP2L2 | 6.07E-05 | 2.73754 |
| BATF3 | 0.00898632 | 2.54313 |
| BC015720 | 0.0259338 | 2.09099 |
| BC018676 | 0.0285543 | 1.5449 |
| BCAT2 | 0.0201657 | 1.72932 |
| BCL2L1 | 0.0380866 | 1.79018 |
| BCL7A | 0.0215287 | 1.63895 |
| BDH1 | 0.0175928 | 1.91553 |
| BDP1 | 0.00970422 | 1.92587 |
| BF089459 | 0.00943964 | 1.80747 |
| BHLHE41 | 0.0448045 | 1.75143 |
| BOLA3 | 0.00383653 | 1.79354 |
| BRI3BP | 0.0135042 | 2.01561 |
| BRIX1 | 0.0169819 | 1.67272 |
| BTNL8 | 0.00425311 | 1.65842 |
| BUB1B | 0.000435752 | 1.94163 |
| BX117927 | 0.0209721 | 1.70318 |
| BX281397 | 0.026255 | 1.7411 |
| BYSL | 0.0271653 | 1.51243 |
| C10orf10 | 0.00353821 | 3.31929 |
| C10orf114 | 0.00568972 | 1.72221 |
| C10orf35 | 0.0335529 | 3.7892 |
| C11orf82 | 0.0194213 | 4.43999 |
| C12orf11 | 0.0238508 | 1.50801 |
| C13orf1 | 0.000122074 | 2.62598 |
| C13orf15 | 2.62E-07 | 14.6714 |
| C15orf27 | 0.00404498 | 2.50256 |
| C16orf52 | 0.00390259 | 1.73424 |
| C17orf60 | 0.000858454 | 2.03635 |
| C17orf86 | 0.025318 | 1.83197 |
| C18orf25 | 0.0260589 | 1.56918 |
| C18orf45 | 0.00397139 | 1.50172 |
| C19orf28 | 0.00430595 | 1.65854 |
| C1GALT1 | 0.00215705 | 1.59805 |
| C1orf133 | 0.0202853 | 2.34807 |
| C1orf212 | 0.00220568 | 1.62493 |
| C20orf24 | 0.00369923 | 1.50642 |
| C21orf122 | 0.0166038 | 2.05472 |
| C21orf70 | 0.00668768 | 1.6896 |
| C2CD2 | 0.00262878 | 3.06126 |
| C2CD3 | 0.0060453 | 1.73463 |
| C4orf41 | 0.0402781 | 2.01326 |
| C4orf49 | 0.016416 | 1.95274 |
| C5orf30 | 0.00612348 | 1.74397 |
| C5orf62 | 0.00104575 | 2.68798 |
| C6orf115 | 0.0354937 | 1.54506 |
| C6orf132 | 0.0421775 | 1.8966 |
| C6orf145 | 0.00469937 | 2.7741 |
| C7orf11 | 0.000410434 | 1.59829 |
| C7orf40 | 0.0117825 | 1.72171 |
| C7orf58 | 0.00730116 | 1.88498 |
| C8orf33 | 0.0196014 | 1.54245 |
| C8orf34 | 0.00246914 | 1.62667 |
| C8orf41 | 0.004661 | 2.24102 |
| C9orf3 | 0.000724113 | 1.70108 |
| CACNB2 | 0.00131958 | 6.52336 |
| CACNG1 | 0.000386188 | 2.89126 |
| CALCOCO2 | 0.000105814 | 2.53902 |
| CALD1 | 0.000591277 | 1.656 |
| CALM1 | 0.0143639 | 1.58041 |
| CALM2 | 0.00141987 | 1.74122 |
| CAP1 | 0.00126086 | 2.14115 |
| CAPN13 | 0.0184631 | 1.57646 |
| CAPZB | 0.0165891 | 1.5644 |
| CASC5 | 0.0356005 | 1.61707 |
| CAV1 | 0.00738071 | 1.78148 |
| CAV2 | 0.000366802 | 2.18943 |
| CC2D1B | 0.0078038 | 1.56607 |
| CCBE1 | 0.0141097 | 1.60769 |
| CCDC107 | 1.65E-05 | 2.40807 |
| CCDC3 | 0.00668644 | 2.1364 |
| CCDC69 | 0.0156379 | 1.88798 |
| CCK | 0.0121874 | 4.09324 |
| CCL26 | 0.00408139 | 2.02649 |
| CCND3 | 0.0010531 | 2.04972 |
| CCNYL1 | 0.00902398 | 1.96634 |
| CD14 | 0.0145938 | 1.80287 |
| CD302 | 2.78E-05 | 2.54987 |
| CD3EAP | 0.000780794 | 2.15231 |
| CDC25A | 0.048407 | 2.06136 |
| CDC42SE2 | 0.00186692 | 1.56152 |
| CDCA8 | 0.0109292 | 1.92704 |
| CDH15 | 0.00986084 | 1.61866 |
| CDH4 | 9.06E-05 | 7.75212 |
| CDK1 | 0.0442114 | 1.54541 |
| CDK2AP2 | 0.00030524 | 1.81775 |
| CDK7 | 0.000617578 | 1.57751 |
| CDR2 | 0.0140856 | 1.93635 |
| CDS2 | 0.00213159 | 1.54322 |
| CECR5 | 0.00215084 | 1.53166 |
| CEP72 | 0.0140295 | 1.9912 |
| CEPT1 | 0.0119064 | 1.5541 |
| CFL2 | 0.00229546 | 1.82062 |
| CFLAR | 3.76E-05 | 2.07114 |
| CHAC2 | 0.00634691 | 3.03771 |
| CHML | 0.0130746 | 1.73727 |
| CHMP7 | 0.0169167 | 1.50612 |
| CHORDC1 | 0.00534041 | 1.56456 |
| CHST3 | 0.0100516 | 1.74095 |
| CITED2 | 0.00290997 | 2.99945 |
| CITED4 | 0.00168274 | 1.67453 |
| CLDN7 | 0.0176379 | 1.68259 |
| CLIC3 | 0.00977455 | 2.27497 |
| CLIC4 | 0.0478357 | 1.66809 |
| CLN6 | 0.0140069 | 1.54237 |
| CLOCK | 0.00107842 | 1.50946 |
| CLPB | 0.00109815 | 1.53449 |
| CMAS | 0.00218293 | 1.65089 |
| CMPK1 | 0.00105891 | 1.56146 |
| CMTM4 | 0.000703957 | 2.05852 |
| CNN1 | 0.000927996 | 2.13755 |
| CNN2 | 0.00447309 | 1.82267 |
| COL4A1 | 0.0242385 | 2.3458 |
| COL4A4 | 0.000146372 | 4.38547 |
| COL8A1 | 0.000677188 | 2.87244 |
| COMP | 0.0179911 | 3.79376 |
| COMTD1 | 0.00114069 | 1.66216 |
| COPS8 | 0.000920585 | 1.56762 |
| COQ2 | 0.00824758 | 1.86736 |
| CORIN | 0.00011104 | 18.926 |
| CORO2A | 0.000627059 | 2.30149 |
| CORO6 | 0.0356902 | 1.96686 |
| COTL1 | 0.00475406 | 1.95613 |
| COX11 | 0.000564414 | 1.5896 |
| CPD | 0.00862876 | 1.52138 |
| CPNE7 | 0.0094181 | 2.2319 |
| CPPED1 | 7.56E-05 | 2.82902 |
| CR591764 | 0.00103703 | 1.58209 |
| CR613891 | 0.00076072 | 1.70398 |
| CREB3L2 | 0.0111498 | 1.73183 |
| CRHR1 | 0.00164673 | 1.72203 |
| CRISPLD2 | 0.00425381 | 5.98415 |
| CRLS1 | 0.000287506 | 1.58396 |
| CRYAB | 0.00125352 | 2.04061 |
| CSRP1 | 0.00783795 | 1.745 |
| CTGF | 0.00182002 | 4.64746 |
| CTHRC1 | 0.0296163 | 1.77053 |
| CTPS | 0.000648912 | 2.80147 |
| CUL1 | 0.0281425 | 1.655 |
| CXorf64 | 0.0328745 | 1.7743 |
| CYB5R2 | 0.000420705 | 1.55885 |
| CYCS | 0.0050165 | 1.80026 |
| CYP1B1 | 0.041785 | 1.68606 |
| CYP39A1 | 0.00327377 | 1.80831 |
| CYR61 | 0.000615664 | 3.3776 |
| CYTH3 | 0.00241759 | 1.77818 |
| DARS2 | 0.0311911 | 2.12351 |
| DCAF6 | 0.0022425 | 1.58922 |
| DCUN1D3 | 0.00250732 | 1.6707 |
| DDAH1 | 0.000510116 | 3.87664 |
| DDX21 | 0.00700511 | 2.01531 |
| DGKI | 0.011094 | 2.09812 |
| DHODH | 0.0043643 | 1.65446 |
| DHRS3 | 8.32E-05 | 2.86433 |
| DHX37 | 0.00214721 | 1.62903 |
| DIMT1L | 0.0014444 | 1.5748 |
| DIO3 | 0.000113238 | 2.0257 |
| DKFZp451A211 | 0.000301512 | 1.87984 |
| DKFZp667E0512 | 0.0342509 | 3.14038 |
| DKK1 | 0.0164912 | 3.45284 |
| DLAT | 0.00271738 | 1.50594 |
| DLK2 | 0.000324658 | 2.51508 |
| DMD | 0.000111666 | 2.59256 |
| DNAJB11 | 0.0144614 | 1.54622 |
| DNAJB14 | 0.00687034 | 1.61754 |
| DNAJB4 | 0.000652459 | 2.78787 |
| DNAJC6 | 0.00467892 | 7.15504 |
| DNM1L | 0.00187597 | 1.5099 |
| DNMT1 | 0.0108799 | 1.67168 |
| DOCK5 | 0.0268186 | 1.73493 |
| DPP3 | 0.00315159 | 1.94291 |
| DSP | 5.25E-05 | 2.06855 |
| DSTN | 0.000116642 | 1.8011 |
| DTL | 0.00948385 | 2.87563 |
| DUS2L | 0.0111845 | 1.6062 |
| DUSP1 | 0.000517894 | 4.46036 |
| DUSP14 | 0.00645673 | 1.75777 |
| DUSP2 | 0.00576529 | 5.47548 |
| DUSP23 | 0.000784954 | 1.85629 |
| DUSP5 | 0.00272524 | 3.76434 |
| DZIP1L | 0.00728414 | 1.84314 |
| EARS2 | 0.0354672 | 1.96077 |
| EBNA1BP2 | 0.0412444 | 1.50937 |
| ECE2 | 0.00453902 | 1.82662 |
| EDN1 | 0.000744979 | 4.15572 |
| EEF1E1 | 0.0176039 | 1.95278 |
| EIF3J | 0.00344654 | 1.69225 |
| EIF5A | 0.00687237 | 1.53395 |
| ELL2 | 0.0030431 | 1.68095 |
| ELN | 0.000137263 | 2.74494 |
| ELOVL5 | 0.000177332 | 1.6439 |
| ELOVL6 | 0.00118634 | 2.43023 |
| EMB | 0.00344032 | 1.5338 |
| EMG1 | 0.000239813 | 1.79926 |
| EMID2 | 0.0204304 | 1.85558 |
| EML2 | 0.00148712 | 1.99023 |
| EMP1 | 0.00286905 | 2.21706 |
| ENDOD1 | 0.00202579 | 3.32077 |
| ENST00000309178 | 0.0112816 | 2.17851 |
| ENST00000342645 | 0.0171677 | 1.72318 |
| ENST00000342688 | 0.0386791 | 1.69366 |
| ENST00000344759 | 0.0376936 | 2.55786 |
| ENST00000370129 | 0.000765317 | 3.18594 |
| ENST00000371468 | 0.0406794 | 1.58155 |
| ENST00000375322 | 0.0223741 | 1.65451 |
| ENST00000379816 | 0.00873272 | 6.17667 |
| ENST00000380464 | 0.0210921 | 2.54818 |
| ENST00000381177 | 0.00955047 | 2.70819 |
| ENST00000381747 | 0.0137814 | 1.86971 |
| ENST00000391684 | 0.00502906 | 3.4201 |
| ENST00000398570 | 0.00570546 | 1.68525 |
| ENST00000401723 | 0.02165 | 1.82102 |
| ENST00000402541 | 0.0387437 | 1.94574 |
| ENTPD6 | 0.00113044 | 1.52246 |
| EPDR1 | 0.0112792 | 1.52477 |
| EPSTI1 | 0.00164476 | 1.76095 |
| EPT1 | 0.00325121 | 2.15321 |
| ERGIC1 | 0.000600872 | 1.61379 |
| ERI2 | 0.0311751 | 1.7135 |
| ERMP1 | 0.0392327 | 1.50686 |
| ERRFI1 | 6.51E-06 | 6.72975 |
| ETV5 | 0.0470441 | 2.27653 |
| EXOC6 | 0.00374814 | 1.79155 |
| EXOC8 | 0.0361861 | 1.62254 |
| F12 | 0.0191964 | 2.0356 |
| F3 | 0.00587573 | 2.93463 |
| FADS3 | 0.017443 | 2.3229 |
| FAIM3 | 0.0263128 | 1.66778 |
| FAM105A | 0.000585894 | 4.17269 |
| FAM107A | 0.00118348 | 18.8137 |
| FAM180A | 0.00622425 | 1.57075 |
| FAM18B | 0.00870345 | 1.55305 |
| FAM196A | 0.00573893 | 3.96213 |
| FAM199X | 0.00158461 | 1.71268 |
| FAM35A | 0.00628827 | 1.7023 |
| FAM38A | 0.021029 | 1.62738 |
| FAM3C | 0.00469855 | 1.84533 |
| FAM43A | 0.0138677 | 1.84512 |
| FAM63B | 0.0294288 | 1.68436 |
| FAM71F1 | 0.0292779 | 1.72651 |
| FAM82A1 | 0.00242605 | 1.59938 |
| FAM83D | 0.0157448 | 4.18493 |
| FAM98A | 0.00269096 | 1.781 |
| FARSB | 0.014691 | 1.6357 |
| FBN2 | 0.00591977 | 5.94083 |
| FBXL16 | 0.00684902 | 3.83202 |
| FCRL1 | 0.0309379 | 1.55178 |
| FEM1B | 0.0425797 | 1.78095 |
| FEN1 | 0.0121872 | 2.7482 |
| FERMT2 | 0.0017139 | 1.6455 |
| FGD4 | 5.68E-05 | 3.07367 |
| FIBIN | 0.0202119 | 2.49963 |
| FICD | 0.00574315 | 1.71092 |
| FJX1 | 0.00260063 | 2.52088 |
| FKBP11 | 0.00112502 | 1.71624 |
| FKBP1B | 0.0010907 | 1.7838 |
| FKBP5 | 4.47E-05 | 24.5415 |
| FKRP | 0.0141173 | 1.5027 |
| FLJ35946 | 0.0234003 | 1.57815 |
| FLJ36031 | 0.000154042 | 1.8524 |
| FLJ39639 | 0.0428632 | 2.13514 |
| FLJ45121 | 0.0492281 | 1.59918 |
| FLNB | 0.00238152 | 1.65867 |
| FLVCR1 | 0.00672422 | 1.54057 |
| FMO2 | 0.042389 | 4.66511 |
| FMO3 | 0.0479171 | 3.20939 |
| FOS | 0.0426497 | 1.6522 |
| FOXO1 | 0.00298527 | 3.98727 |
| FOXO3 | 5.76E-05 | 1.64091 |
| FRMD6 | 0.00244582 | 1.59677 |
| FRMPD4 | 0.0221268 | 1.53845 |
| FSTL3 | 2.28E-05 | 3.36628 |
| FUS | 0.00614092 | 2.09592 |
| FZD4 | 0.00030571 | 1.51035 |
| FZD6 | 0.0154349 | 2.32915 |
| FZD8 | 0.00307759 | 2.07083 |
| GABBR2 | 0.000606108 | 4.2317 |
| GABRP | 0.00172278 | 1.8987 |
| GADD45B | 7.19E-05 | 5.09742 |
| GALE | 0.00138644 | 1.80455 |
| GALNT10 | 0.00413617 | 1.57958 |
| GALNTL2 | 0.0372564 | 2.1186 |
| GAS6 | 0.0305083 | 2.37005 |
| GBP1 | 0.0379236 | 1.91162 |
| GCA | 0.0364882 | 1.61797 |
| GCAT | 0.00163249 | 2.01689 |
| GCHFR | 0.0162625 | 1.98733 |
| GCNT1 | 0.000165363 | 2.66657 |
| GCNT4 | 0.0453917 | 1.89081 |
| GDNF | 0.0155861 | 2.2092 |
| GFOD1 | 0.00345263 | 1.87827 |
| GFPT2 | 0.0147677 | 1.70647 |
| GGT5 | 1.57E-07 | 31.6064 |
| GHRLOS | 0.0310255 | 1.59209 |
| GIT1 | 0.00343361 | 2.62461 |
| GLO1 | 0.00473179 | 1.50251 |
| GLRX | 0.00331842 | 2.07595 |
| GLRX2 | 0.00177317 | 1.68739 |
| GLS | 0.00505895 | 1.94256 |
| GLT25D1 | 0.00101826 | 1.61507 |
| GLUL | 0.0011947 | 7.15485 |
| GMFB | 0.000446641 | 1.61869 |
| GMPR | 0.000898012 | 2.3838 |
| GNAQ | 0.0386193 | 1.52305 |
| GNB4 | 0.0018707 | 1.65659 |
| GNL2 | 0.0174807 | 1.50562 |
| GORASP1 | 0.0219735 | 2.74537 |
| GPATCH4 | 0.00964751 | 1.87799 |
| GPC4 | 0.00550563 | 3.51117 |
| GPD1L | 0.0118705 | 1.73073 |
| GPM6B | 0.000350995 | 9.13434 |
| GPN3 | 0.00836536 | 1.6516 |
| GPRC5B | 0.00197273 | 2.29286 |
| GPX3 | 4.29E-05 | 8.01139 |
| GRAMD3 | 0.0012679 | 3.48387 |
| GRPEL1 | 0.000456194 | 1.77487 |
| GSTA2 | 3.80E-05 | 2.82349 |
| GSTO1 | 0.00857013 | 1.50741 |
| GSTT2 | 0.00629414 | 1.69291 |
| GSTT2B | 0.00922715 | 1.65416 |
| GTF2H3 | 0.0358904 | 1.57761 |
| GTPBP4 | 0.00824767 | 1.76664 |
| GYS1 | 6.20E-05 | 1.9165 |
| H2AFJ | 7.03E-05 | 1.82289 |
| HAP1 | 0.0123286 | 1.62579 |
| HBM | 0.0487227 | 1.56113 |
| HEG1 | 0.00335769 | 1.91159 |
| HES4 | 0.0328722 | 1.88126 |
| HHEX | 0.0317244 | 1.94519 |
| HIAT1 | 0.0441837 | 1.75803 |
| HIF1A | 0.000344957 | 2.07644 |
| HIGD1A | 0.000152984 | 2.16616 |
| HIP1 | 0.000727501 | 2.20309 |
| HIST1H2AJ | 0.0235796 | 1.71767 |
| HIST2H3A | 0.0114325 | 2.35158 |
| HK2 | 0.00371552 | 2.10503 |
| HMG20A | 0.00592095 | 1.68022 |
| HMGN4 | 0.0274894 | 1.60598 |
| HMHA1 | 0.0389258 | 1.85781 |
| HMMR | 0.00678003 | 4.14906 |
| HN1 | 0.0142202 | 1.73664 |
| HN1L | 0.029468 | 1.50678 |
| HNMT | 0.00102323 | 1.90179 |
| HNRNPAB | 0.000598532 | 1.80191 |
| HPR | 0.0482795 | 1.56309 |
| HPS5 | 0.00313115 | 3.1152 |
| HRCT1 | 0.0258425 | 2.24307 |
| HSD11B1 | 1.97E-06 | 8.26383 |
| HSP90B3P | 0.0374495 | 1.56387 |
| HSPA2 | 0.00258998 | 6.03287 |
| HSPA4 | 0.047252 | 3.88146 |
| HSPB1 | 0.000434503 | 1.70551 |
| HSPB3 | 0.00260407 | 5.36834 |
| ID1 | 0.00148081 | 11.5961 |
| ID2 | 0.0289192 | 1.53116 |
| ID3 | 6.66E-05 | 4.99738 |
| ID4 | 0.0192484 | 2.54012 |
| IFI44L | 0.0028703 | 5.45103 |
| IFRD2 | 0.00183076 | 1.57166 |
| IGF2BP2 | 5.09E-05 | 1.79861 |
| IGFBP2 | 4.23E-05 | 21.7862 |
| IL7R | 0.0152012 | 3.47129 |
| IMPA1 | 0.0103166 | 1.50331 |
| IMPA2 | 4.10E-05 | 3.03187 |
| IMPAD1 | 0.0112029 | 1.55092 |
| IMPDH1 | 2.26E-05 | 1.56363 |
| INHBA | 0.0199413 | 1.68187 |
| INHBB | 0.0325609 | 6.34658 |
| INMT | 0.00439142 | 2.26035 |
| INPP5A | 0.00466474 | 1.83103 |
| INSIG2 | 0.00322164 | 1.8438 |
| INTS7 | 0.00352402 | 1.62503 |
| IRAK1 | 0.000523001 | 1.56195 |
| IRAK3 | 0.00206313 | 1.7672 |
| IRS2 | 0.00239697 | 2.03994 |
| ISCA1 | 0.00059426 | 1.61281 |
| ITGA1 | 4.02E-05 | 5.22321 |
| ITGA10 | 0.000114165 | 15.3188 |
| ITGA4 | 0.00208014 | 1.93316 |
| ITGA5 | 0.000328959 | 2.38929 |
| ITGAM | 0.0171557 | 1.53203 |
| ITGAV | 0.0166644 | 1.61931 |
| ITGBL1 | 0.000451531 | 2.1489 |
| ITPR1 | 0.00485954 | 1.92171 |
| ITPRIPL1 | 0.00475445 | 2.66566 |
| ITSN1 | 0.000427323 | 1.77027 |
| JAK1 | 0.0320985 | 1.98615 |
| JAK2 | 0.0207233 | 1.72166 |
| JMJD6 | 0.00192298 | 2.03299 |
| KANK1 | 0.00195516 | 2.28631 |
| KCNJ8 | 0.000440746 | 1.55589 |
| KCNK1 | 0.00182328 | 1.71816 |
| KCNK6 | 1.36E-05 | 14.1623 |
| KCNS3 | 0.00249143 | 2.57135 |
| KCTD10 | 0.00761122 | 1.68378 |
| KCTD20 | 0.00226884 | 1.88703 |
| KCTD9 | 4.78E-05 | 1.76099 |
| KIAA0355 | 0.00374466 | 1.69792 |
| KIAA1279 | 0.00310948 | 1.58319 |
| KIAA1715 | 0.00129522 | 1.69758 |
| KIAA1919 | 0.047804 | 2.39783 |
| KIF2C | 0.0314705 | 1.77891 |
| KIF4A | 0.00596994 | 1.65738 |
| KLF6 | 5.05E-05 | 2.02588 |
| KLF7 | 0.00853556 | 1.9368 |
| KLF9 | 7.94E-05 | 3.49055 |
| KLHL18 | 0.00386521 | 1.59815 |
| KLK4 | 0.0163475 | 1.84584 |
| KRT34 | 0.0474932 | 2.88706 |
| KRT7 | 0.00456122 | 1.74104 |
| KRT83 | 0.0131571 | 2.45492 |
| KRTAP1-5 | 0.01191 | 3.40727 |
| KRTAP19-1 | 0.0119469 | 1.74836 |
| KRTAP19-5 | 0.0162567 | 1.90894 |
| LACTB | 0.00900954 | 1.97928 |
| LAMA2 | 0.0355337 | 2.46079 |
| LARP4 | 0.000435263 | 1.52557 |
| LASP1 | 0.00813053 | 1.84552 |
| LCE1A | 0.00208654 | 1.69475 |
| LCLAT1 | 0.00284145 | 1.81298 |
| LDHA | 2.86E-05 | 2.74005 |
| LEPROT | 0.0443616 | 1.61567 |
| LGALS8 | 3.79E-05 | 1.76165 |
| LGI3 | 0.0249623 | 1.63375 |
| LIMS2 | 0.00136723 | 2.04097 |
| LIX1L | 0.000967884 | 1.62422 |
| LMCD1 | 0.00456519 | 2.75796 |
| LMO1 | 0.0487749 | 2.21126 |
| LMO7 | 0.00218403 | 2.45906 |
| LMOD1 | 7.70E-05 | 2.08724 |
| LOC100128054 | 0.00905401 | 5.8884 |
| LOC100128170 | 0.0303555 | 3.01491 |
| LOC100128469 | 0.0287304 | 2.78275 |
| LOC100128572 | 0.0248181 | 2.10543 |
| LOC100130431 | 0.0160118 | 2.61199 |
| LOC100130433 | 0.0035588 | 2.52466 |
| LOC100130539 | 0.00055612 | 1.57521 |
| LOC100131907 | 0.0234913 | 1.60614 |
| LOC100132509 | 0.00655627 | 1.87943 |
| LOC100132724 | 0.0100022 | 1.55313 |
| LOC100133047 | 0.00505914 | 1.87653 |
| LOC100133478 | 0.00619115 | 1.66708 |
| LOC100134228 | 0.0149882 | 1.71291 |
| LOC100287322 | 0.0325158 | 2.20346 |
| LOC100292409 | 0.0298446 | 1.87766 |
| LOC145694 | 0.00263699 | 2.3731 |
| LOC148709 | 0.012854 | 2.03727 |
| LOC153577 | 0.0226333 | 1.53363 |
| LOC171220 | 0.00155567 | 1.76534 |
| LOC284441 | 0.00440985 | 1.60981 |
| LOC338620 | 0.00431317 | 1.74271 |
| LOC344382 | 0.000644331 | 1.62984 |
| LOC389842 | 0.00200008 | 1.79748 |
| LOC390424 | 0.00537042 | 1.5698 |
| LOC391334 | 0.0001362 | 3.18579 |
| LOC400743 | 0.00229257 | 1.77153 |
| LOC440028 | 0.0349686 | 1.75812 |
| LOC642513 | 0.00107543 | 1.82094 |
| LOC643650 | 0.026226 | 1.51865 |
| LOC644196 | 0.0236459 | 1.54117 |
| LOC644538 | 0.00960877 | 1.63489 |
| LOC644992 | 0.0319259 | 1.52208 |
| LOC646048 | 6.96E-05 | 2.1439 |
| LOC646821 | 0.00509764 | 1.86225 |
| LOC648740 | 0.000201191 | 1.85684 |
| LOC650638 | 0.000511304 | 1.55213 |
| LOC728975 | 0.026075 | 1.60045 |
| LOC729314 | 0.00417713 | 2.76233 |
| LOC729454 | 0.000483034 | 2.74461 |
| LOC732043 | 0.0157444 | 2.13893 |
| LOH3CR2A | 0.0343335 | 4.92904 |
| LOX | 0.00481438 | 1.73426 |
| LOXL4 | 0.00158302 | 1.85236 |
| LPCAT1 | 0.035448 | 2.08812 |
| LPO | 0.0312063 | 1.64222 |
| LRCH2 | 0.0154795 | 2.18619 |
| LRCH3 | 0.0314238 | 1.70589 |
| LRP8 | 0.0157511 | 2.64518 |
| LRRC1 | 0.000724046 | 1.74413 |
| LRRC16A | 0.00184406 | 2.35228 |
| LRRC59 | 0.00112062 | 2.06838 |
| LRRFIP1 | 0.00120508 | 1.73099 |
| LTBP1 | 0.0182432 | 1.78559 |
| LYN | 0.02124 | 1.54022 |
| MAFF | 0.00918433 | 1.76228 |
| MAK16 | 0.000694019 | 1.94727 |
| MAMDC2 | 0.0436927 | 3.5881 |
| MANF | 0.0070126 | 1.87631 |
| MAOA | 0.000549328 | 5.70575 |
| MAP2 | 0.000470111 | 4.27124 |
| MAP2K1 | 0.00609203 | 1.66206 |
| MAP2K3 | 0.00075281 | 1.77848 |
| MAP4K3 | 0.00114237 | 2.57093 |
| MAP6D1 | 0.0231176 | 2.09888 |
| MAPKAP1 | 0.0080473 | 1.55029 |
| MAPRE1 | 0.00122769 | 1.7136 |
| MAT2B | 0.0121626 | 1.54906 |
| MBNL1 | 0.00193886 | 1.83515 |
| MBNL3 | 0.0136999 | 1.52966 |
| MCAM | 0.0010333 | 2.67544 |
| MCTP1 | 0.0349265 | 1.78987 |
| MELK | 0.0202536 | 2.01987 |
| MET | 0.00275927 | 1.7376 |
| METTL7A | 0.00157971 | 2.12329 |
| MFSD6 | 0.00273228 | 2.00061 |
| MGC13005 | 0.0223342 | 1.79279 |
| MGLL | 0.00365998 | 1.97383 |
| MICAL2 | 0.00480053 | 2.35379 |
| MID2 | 0.0324754 | 1.88763 |
| MLX | 0.0254497 | 1.58706 |
| MLYCD | 0.0267758 | 1.94727 |
| MMD | 0.000770253 | 3.10212 |
| MMP24 | 0.00128928 | 3.42594 |
| MOBKL2B | 9.03E-06 | 4.48098 |
| MOCS1 | 0.032875 | 1.70975 |
| MORF4L2 | 0.00131217 | 2.4464 |
| MOSPD1 | 0.0452301 | 1.70849 |
| MOSPD2 | 0.00107444 | 2.19909 |
| MPHOSPH6 | 0.0186178 | 1.55903 |
| MPP3 | 0.0322682 | 1.84216 |
| MREG | 0.019026 | 1.89738 |
| MRM1 | 0.000390098 | 1.96207 |
| MRPL15 | 0.00247567 | 1.59242 |
| MRPL24 | 0.00214621 | 1.51102 |
| MRPL35 | 0.0356573 | 1.6102 |
| MRVI1 | 0.0167316 | 2.10683 |
| MSI1 | 0.00623841 | 1.62439 |
| MSRB3 | 5.95E-06 | 2.10393 |
| MST152 | 0.0189731 | 1.60769 |
| MT1A | 0.0100966 | 4.33904 |
| MT1B | 0.00894133 | 5.28435 |
| MT1E | 0.00887961 | 4.30571 |
| MT1H | 0.0115931 | 5.13602 |
| MT1L | 0.0136289 | 5.08289 |
| MT1M | 0.00143241 | 3.83056 |
| MT1X | 0.00787725 | 5.56449 |
| MT2A | 0.0269966 | 3.07828 |
| MTMR10 | 3.03E-06 | 1.98148 |
| MTP18 | 0.02161 | 2.19339 |
| MTRR | 0.0399784 | 1.99409 |
| MYADM | 5.96E-05 | 2.60941 |
| MYBBP1A | 0.00391775 | 2.17415 |
| MYC | 0.00134459 | 2.44254 |
| MYCBP | 0.00711403 | 1.80784 |
| MYH9 | 0.0171027 | 1.65611 |
| MYL12A | 0.00141238 | 2.03355 |
| MYL6 | 0.000413247 | 1.85318 |
| MYLK | 0.000152546 | 1.80313 |
| MYO16 | 0.0400855 | 1.87721 |
| MYO1E | 0.0032029 | 2.38892 |
| MYOF | 0.00278865 | 1.5228 |
| NAA15 | 0.0307623 | 1.70389 |
| NALCN | 0.0113119 | 1.90744 |
| NANOS1 | 0.00118451 | 3.00316 |
| NANS | 0.00877281 | 1.7245 |
| NAP1L1 | 0.00034077 | 1.59209 |
| NAPG | 0.00227128 | 1.6776 |
| NAV3 | 0.000306592 | 3.30819 |
| NCEH1 | 0.0349027 | 1.52558 |
| NCLN | 0.00073739 | 1.50773 |
| NCOA3 | 0.0415141 | 2.48846 |
| NCRNA00086 | 0.040504 | 1.53802 |
| NCRNA00181 | 0.00467047 | 1.56421 |
| NDFIP2 | 0.00601615 | 1.63551 |
| NEDD4 | 0.000374131 | 1.7314 |
| NEDD9 | 0.00361089 | 2.68927 |
| NEFH | 0.0117534 | 2.35413 |
| NEGR1 | 0.000278763 | 1.54388 |
| NEXN | 4.00E-05 | 3.79921 |
| NF2 | 0.0154318 | 1.64741 |
| NFYB | 0.0143623 | 1.65111 |
| NIPSNAP3A | 0.000575389 | 1.91787 |
| NLN | 0.000158388 | 2.5042 |
| NLRP1 | 0.0275868 | 1.51269 |
| NNMT | 0.00843306 | 3.05479 |
| NOC3L | 0.00673936 | 1.55059 |
| NOP16 | 0.00196456 | 2.43156 |
| NOP2 | 0.00168314 | 1.65084 |
| NOX4 | 0.0308222 | 2.00879 |
| NPAS1 | 0.0110908 | 1.86472 |
| NPAS2 | 0.00497926 | 2.44902 |
| NPTN | 0.00135166 | 1.61457 |
| NR2C2AP | 0.0104311 | 1.77092 |
| NT5DC3 | 0.00810746 | 4.10666 |
| NUAK2 | 0.0190824 | 2.29629 |
| NUDCD2 | 0.000662481 | 1.78942 |
| NUDT19 | 0.0388857 | 1.5359 |
| NUP93 | 0.00381581 | 1.84318 |
| ODC1 | 0.0116225 | 1.83711 |
| ODZ2 | 0.0323452 | 2.68394 |
| OGFRL1 | 0.0155681 | 2.00771 |
| OMD | 0.00406637 | 5.04036 |
| OPN1MW | 0.00373567 | 1.59801 |
| OPN3 | 0.00970076 | 1.742 |
| OR5V1 | 0.0194198 | 2.21916 |
| ORMDL2 | 0.000628362 | 1.61841 |
| OSTM1 | 0.00818916 | 1.51149 |
| OXCT1 | 7.82E-05 | 1.61689 |
| OXCT2 | 0.0330097 | 1.8024 |
| OXNAD1 | 0.0275821 | 2.14378 |
| OXSR1 | 0.00552271 | 1.55198 |
| OXTR | 0.00663212 | 4.02779 |
| P704P | 0.00146524 | 2.26153 |
| PACSIN2 | 0.00252185 | 1.85807 |
| PALLD | 0.00437558 | 1.84501 |
| PAPSS2 | 0.0346081 | 1.94547 |
| PAQR4 | 0.00484029 | 3.53125 |
| PAQR7 | 0.0263947 | 1.57708 |
| PAWR | 0.000667527 | 3.06445 |
| PCDH10 | 0.00441603 | 1.72327 |
| PCDH7 | 0.0206115 | 4.16855 |
| PCGF5 | 0.00274295 | 1.81379 |
| PCSK7 | 0.0223914 | 1.6155 |
| PCTP | 0.00112485 | 1.70939 |
| PDE12 | 0.00588446 | 1.60991 |
| PDIA6 | 0.00937482 | 1.60215 |
| PDLIM5 | 0.00162246 | 2.93322 |
| PDPN | 0.00185418 | 2.60925 |
| PDSS1 | 0.0105606 | 1.90148 |
| PER1 | 0.000305321 | 4.92767 |
| PFKP | 0.00163806 | 2.1042 |
| PGAM5 | 0.00695763 | 2.15301 |
| PGK1 | 3.96E-05 | 1.78875 |
| PGM1 | 0.000709579 | 2.31422 |
| PGM2 | 0.0209391 | 1.55956 |
| PHC2 | 0.000417673 | 2.1495 |
| PHF17 | 0.0168954 | 1.6189 |
| PHLDA2 | 0.0319418 | 1.87031 |
| PHLPP2 | 0.0370219 | 1.60966 |
| PICALM | 0.00690502 | 1.56393 |
| PIGF | 0.000449647 | 1.53771 |
| PIK3CA | 0.00985671 | 1.53998 |
| PIK3R1 | 0.015084 | 2.29741 |
| PITRM1 | 0.0223871 | 1.60955 |
| PKMYT1 | 0.0280647 | 1.90622 |
| PLAUR | 0.0494297 | 1.73145 |
| PLB1 | 0.000120762 | 1.92092 |
| PLCB2 | 0.0287452 | 2.35605 |
| PLCB4 | 0.00850634 | 2.11344 |
| PLIN2 | 0.02004 | 1.55784 |
| PLSCR4 | 0.00024403 | 1.62773 |
| PNO1 | 0.0211988 | 1.56016 |
| PNP | 0.0113601 | 2.28148 |
| POLA2 | 0.00257854 | 1.91417 |
| POLR2D | 0.00346678 | 1.54752 |
| POMP | 0.00797329 | 1.60143 |
| POP1 | 0.00364109 | 1.93839 |
| POTEE | 0.00139713 | 2.3438 |
| POTEF | 0.00219608 | 2.66877 |
| POTEKP | 0.00339494 | 2.35116 |
| POU5F1 | 0.0317949 | 1.58527 |
| PPARG | 0.00317051 | 2.82902 |
| PPIF | 0.00923463 | 1.68058 |
| PPME1 | 0.00416942 | 3.44018 |
| PPP1R12B | 0.00212291 | 2.20193 |
| PPP1R13L | 0.00278642 | 1.545 |
| PPP2CB | 0.0035529 | 1.63716 |
| PRDX3 | 0.0359474 | 1.59806 |
| PRELID2 | 0.0211553 | 2.12947 |
| PRKAA1 | 0.00239219 | 1.62941 |
| PRKAG2 | 0.00473294 | 2.57994 |
| PRRG1 | 0.00143288 | 1.72127 |
| PRUNE2 | 0.00344796 | 2.4795 |
| PS1TP4 | 0.00383827 | 2.04853 |
| PSMD14 | 0.0101928 | 1.5473 |
| PTGIR | 0.00422575 | 1.86789 |
| PTPDC1 | 0.0449958 | 1.623 |
| PTPLA | 0.000718643 | 2.26241 |
| PTPLB | 9.96E-05 | 2.7366 |
| PTPRG | 0.0208632 | 1.64082 |
| PTPRJ | 0.00418223 | 2.2317 |
| PTS | 0.00308934 | 1.90644 |
| PUS1 | 0.00188294 | 2.17629 |
| PUS7 | 0.0116894 | 1.76542 |
| PVR | 0.0111923 | 2.39503 |
| PYCRL | 0.00328686 | 1.55817 |
| QPCT | 0.0132233 | 1.81344 |
| QPCTL | 0.0372729 | 2.15939 |
| QTRTD1 | 0.000153599 | 1.67208 |
| RAB11A | 0.000233194 | 1.72663 |
| RAB11FIP1 | 0.0171026 | 2.21453 |
| RAB27A | 0.00878625 | 2.75972 |
| RAB3B | 0.000949471 | 1.9389 |
| RAB6A | 0.00527388 | 1.51121 |
| RAD1 | 0.0102075 | 1.75632 |
| RAD18 | 0.0218541 | 1.72537 |
| RAD51 | 0.00472895 | 2.27748 |
| RAF1 | 0.0091703 | 2.0559 |
| RAI14 | 2.40E-05 | 2.45364 |
| RAI2 | 0.0283049 | 1.54225 |
| RASAL2 | 0.0363796 | 1.79629 |
| RASD1 | 0.0483079 | 1.80964 |
| RASGRP2 | 0.00925799 | 2.1094 |
| RASL11A | 0.000143715 | 5.82576 |
| RASL11B | 4.44E-06 | 12.9278 |
| RASSF7 | 0.0271572 | 2.09258 |
| RBL1 | 0.0102877 | 2.14433 |
| RBM14 | 0.00170459 | 1.7779 |
| RBM28 | 0.0139659 | 1.54316 |
| RFC3 | 0.0212276 | 1.86182 |
| RFT1 | 0.00186395 | 1.54499 |
| RFTN1 | 0.0125043 | 2.12307 |
| RGNEF | 6.35E-05 | 3.21785 |
| RHEBL1 | 0.0245076 | 1.99678 |
| RHOB | 0.0335521 | 1.9914 |
| RHOBTB3 | 0.0438078 | 1.66322 |
| RNASEH1 | 0.000947574 | 1.65913 |
| RND1 | 0.0288267 | 1.57681 |
| RNF10 | 0.0037688 | 1.50764 |
| RNF141 | 0.004625 | 1.57507 |
| RNF144B | 0.00975745 | 4.50221 |
| RNFT2 | 0.018923 | 2.10411 |
| RNGTT | 0.0116454 | 1.59883 |
| ROCK1 | 0.00588993 | 1.65322 |
| ROR1 | 0.000479199 | 1.86101 |
| RP11-631M21.2 | 0.00342656 | 1.8068 |
| RP1L1 | 0.010002 | 1.99475 |
| RP6-213H19.1 | 0.0185728 | 1.67553 |
| RPS6KA2 | 0.00153592 | 1.92873 |
| RRP15 | 0.0220062 | 1.57668 |
| RRP9 | 0.00210564 | 1.58522 |
| RRS1 | 0.0165519 | 1.76946 |
| RSU1 | 0.00238831 | 1.60599 |
| RWDD4A | 0.0142216 | 2.70756 |
| SACM1L | 0.0122133 | 1.53201 |
| SAMD4A | 0.00106824 | 1.78158 |
| SAMD8 | 0.0154548 | 1.57266 |
| SAMHD1 | 0.000545215 | 4.53088 |
| SBDSP1 | 0.00428828 | 1.66906 |
| SBF2 | 0.0333762 | 1.56989 |
| SCHIP1 | 0.0190002 | 1.98449 |
| SCOC | 0.0278135 | 1.53804 |
| SCRG1 | 0.0325803 | 3.61707 |
| SDF2L1 | 0.00382385 | 1.97541 |
| SEC23B | 0.0189812 | 1.80933 |
| SEC24B | 0.0129562 | 1.71893 |
| SEC24D | 0.0200288 | 1.5229 |
| SEH1L | 0.00815479 | 1.60584 |
| SELS | 0.00332456 | 1.93291 |
| SEMA7A | 0.00189548 | 2.56132 |
| SENP3 | 0.0434118 | 1.78581 |
| SEPX1 | 0.000503526 | 1.80563 |
| SERPINB8 | 0.0168154 | 1.89805 |
| SERPINB9 | 0.0074541 | 2.36223 |
| SERPINE1 | 0.00451829 | 2.90668 |
| SFRS12IP1 | 0.0213983 | 1.54034 |
| SFRS2 | 0.00271854 | 1.71331 |
| SFRS3 | 0.0164271 | 1.53682 |
| SFRS7 | 0.00801897 | 1.82342 |
| SFT2D2 | 0.0143098 | 1.57541 |
| SFT2D3 | 0.00767714 | 1.5461 |
| SGEF | 0.00482695 | 3.46207 |
| SGTB | 0.000105275 | 1.87974 |
| SH3BP4 | 0.00919753 | 2.16152 |
| SH3D20 | 0.0346043 | 2.28084 |
| SH3RF3 | 0.0125153 | 2.24038 |
| SHMT1 | 0.0076553 | 2.53877 |
| SLC15A4 | 0.0171889 | 1.69066 |
| SLC16A3 | 6.51E-06 | 2.80281 |
| SLC16A7 | 0.0271554 | 1.62416 |
| SLC17A9 | 0.000241632 | 2.76718 |
| SLC19A2 | 0.000373128 | 1.97064 |
| SLC20A2 | 0.000381756 | 2.33007 |
| SLC25A22 | 0.00771211 | 1.56613 |
| SLC25A32 | 0.000165299 | 1.79321 |
| SLC25A4 | 0.00764102 | 1.79474 |
| SLC25A43 | 0.00264904 | 1.99935 |
| SLC26A6 | 0.00523721 | 1.76833 |
| SLC27A4 | 0.0379219 | 1.7268 |
| SLC2A10 | 0.0027216 | 1.79838 |
| SLC2A3 | 0.0279793 | 1.71771 |
| SLC30A9 | 0.02618 | 3.14354 |
| SLC33A1 | 0.0149954 | 2.01808 |
| SLC35A5 | 0.00677298 | 1.53463 |
| SLC35D1 | 0.00765663 | 1.93924 |
| SLC35F5 | 1.46E-05 | 1.96474 |
| SLC38A1 | 0.0142995 | 1.6862 |
| SLC38A2 | 6.89E-06 | 2.0386 |
| SLC39A13 | 0.00213497 | 1.65273 |
| SLC7A1 | 0.005509 | 2.06222 |
| SLC7A8 | 0.00862549 | 1.77724 |
| SLMAP | 0.0204256 | 1.61493 |
| SMAD2 | 0.00339065 | 1.91172 |
| SMAD7 | 0.0182292 | 1.64949 |
| SMARCD2 | 0.016608 | 2.01797 |
| SNAPC3 | 0.00450977 | 1.58933 |
| SNAR-A3 | 0.00255013 | 2.64077 |
| SNRPB | 0.0254895 | 1.56684 |
| SNX11 | 0.00377199 | 1.5693 |
| SNX24 | 0.000929584 | 1.60624 |
| SNX25 | 0.0166975 | 1.72335 |
| SOAT1 | 0.00558595 | 1.70016 |
| SORT1 | 0.000561637 | 6.95208 |
| SP6 | 0.00436942 | 2.0372 |
| SPARCL1 | 0.000137625 | 13.3686 |
| SPCS3 | 0.00336583 | 2.30769 |
| SPINT2 | 4.09E-05 | 9.59624 |
| SPOCD1 | 0.00476937 | 2.57589 |
| SPON1 | 0.043416 | 1.91547 |
| SPRED1 | 0.0099477 | 1.82514 |
| SPRYD4 | 0.000749324 | 1.53345 |
| SPTY2D1 | 0.0148008 | 1.65567 |
| SQRDL | 0.0142662 | 1.60701 |
| SRD5A3 | 0.0271147 | 1.79026 |
| SRFBP1 | 0.00119695 | 1.58983 |
| SRGN | 0.0106168 | 3.4918 |
| SSB | 0.00436797 | 1.88678 |
| SSH2 | 0.0085552 | 1.98942 |
| STARD4 | 0.0399617 | 1.63979 |
| STBD1 | 0.00778119 | 2.17862 |
| STEAP1 | 2.05E-05 | 2.47197 |
| STEAP2 | 0.0164272 | 1.55831 |
| STK17B | 0.00143338 | 1.65494 |
| STK39 | 0.0360824 | 1.61323 |
| STMN2 | 0.0124131 | 1.89696 |
| STRADB | 0.0475984 | 1.55374 |
| STX11 | 0.00877988 | 1.61584 |
| STX12 | 0.00476075 | 1.68866 |
| STXBP5 | 0.000161413 | 1.50957 |
| SUN5 | 0.046088 | 1.77629 |
| SUSD2 | 0.00334921 | 3.55869 |
| SYNGR2 | 0.0127017 | 2.10133 |
| SYNPO2 | 0.00861348 | 1.8403 |
| SYTL4 | 0.034545 | 1.87459 |
| TACC1 | 0.0219788 | 2.15643 |
| TAF5L | 0.00914092 | 1.72181 |
| TAGLN | 0.00127246 | 2.57925 |
| TBC1D2 | 0.0056754 | 1.91478 |
| TBC1D4 | 0.00288464 | 2.29773 |
| TBRG4 | 0.000972494 | 1.5395 |
| TCEAL1 | 0.0006735 | 1.985 |
| TCEAL2 | 0.000152814 | 2.53398 |
| TCEAL4 | 1.48E-07 | 2.66919 |
| TCEAL5 | 0.000181779 | 2.06662 |
| TCEAL6 | 5.78E-05 | 1.97459 |
| TCF19 | 0.00884368 | 2.00972 |
| TCL1A | 0.0262483 | 4.14782 |
| TEAD4 | 0.00446092 | 1.67768 |
| TEX2 | 0.00181082 | 3.17498 |
| TG | 0.00270778 | 1.69951 |
| TGFBR2 | 0.000542467 | 2.15237 |
| TGM2 | 0.0113118 | 3.10177 |
| THBS1 | 0.00419938 | 3.75451 |
| THC2512536 | 0.00282558 | 3.62487 |
| THC2518325 | 0.0283869 | 1.50972 |
| THC2577776 | 0.0424681 | 2.0161 |
| THC2582201 | 0.00422797 | 1.80013 |
| THC2593778 | 0.00275491 | 3.07198 |
| THC2712321 | 0.00347851 | 1.7806 |
| THC2725860 | 0.0254294 | 1.72317 |
| TIMM17A | 0.012793 | 1.54448 |
| TIMM23B | 0.046832 | 1.68669 |
| TIMM8A | 0.0279726 | 1.64819 |
| TIMP4 | 0.000596621 | 3.85772 |
| TIPARP | 0.0337525 | 2.25644 |
| TJP2 | 0.0395758 | 2.35949 |
| TLR4 | 0.0173275 | 1.65079 |
| TMCO7 | 0.00613852 | 1.7326 |
| TMEM102 | 0.000871043 | 2.8069 |
| TMEM125 | 0.0296226 | 2.06018 |
| TMEM150A | 0.00955452 | 1.59755 |
| TMEM165 | 0.00478942 | 1.54254 |
| TMEM2 | 0.00194436 | 2.11351 |
| TMEM204 | 0.000270621 | 1.64036 |
| TMEM22 | 0.00310699 | 3.07444 |
| TMEM33 | 0.0258675 | 2.11414 |
| TMEM47 | 0.00234727 | 2.01141 |
| TMEM64 | 0.0108316 | 2.12042 |
| TMEM87B | 0.000755833 | 1.86406 |
| TMPPE | 0.0137259 | 2.15756 |
| TMTC1 | 0.0071787 | 2.64456 |
| TNFRSF12A | 0.000272686 | 2.06902 |
| TNR | 0.018188 | 1.67231 |
| TOM1L1 | 0.00213174 | 1.73748 |
| TOM1L2 | 0.000841282 | 1.65143 |
| TP53I11 | 0.0044767 | 1.92896 |
| TP53INP2 | 0.0111494 | 2.12156 |
| TPD52L2 | 0.0393145 | 1.58191 |
| TPM1 | 0.000930686 | 2.38271 |
| TPM2 | 0.00137902 | 1.77676 |
| TPM3 | 0.00591919 | 1.68039 |
| TPM4 | 0.000122668 | 1.75103 |
| TPST2 | 0.026261 | 1.63398 |
| TRAF5 | 0.0463081 | 1.50109 |
| TRAM1 | 0.00273459 | 1.56355 |
| TRAM2 | 0.014686 | 1.54542 |
| TRIM7 | 0.0015507 | 2.32564 |
| TRMT6 | 0.0267684 | 1.69242 |
| TRNP1 | 0.000275421 | 4.63116 |
| TRPM4 | 0.0186554 | 1.83113 |
| TRPV2 | 0.000364361 | 1.56762 |
| TSC22D2 | 0.0498316 | 1.64322 |
| TSC22D3 | 8.11E-05 | 4.33259 |
| TSEN2 | 0.00351912 | 1.52746 |
| TSHB | 0.0116533 | 1.55534 |
| TSPAN17 | 0.019259 | 1.60173 |
| TSR1 | 0.000711684 | 1.71553 |
| TSSC1 | 0.000645801 | 1.52556 |
| TSTD1 | 0.00596731 | 1.53579 |
| TTC13 | 0.00678506 | 1.51201 |
| TTC32 | 0.0148858 | 1.68079 |
| TTF2 | 0.00358706 | 1.69994 |
| TTLL12 | 0.0108027 | 1.52064 |
| TUBA1A | 0.00254599 | 1.54122 |
| TUBA1B | 0.00155946 | 1.99865 |
| TUBA1C | 0.0137498 | 1.79207 |
| TUBB2A | 0.00105395 | 2.05039 |
| TUBB2C | 0.0156315 | 1.6096 |
| TUBB6 | 0.00246731 | 2.45679 |
| TXNDC11 | 0.000172809 | 1.80358 |
| TXNRD1 | 0.00462741 | 3.22285 |
| TYW1B | 0.0254874 | 2.82952 |
| UAP1 | 0.00502418 | 2.55199 |
| UBE2N | 0.0230368 | 1.8434 |
| UBE3B | 0.00382182 | 1.64706 |
| UCP2 | 0.00754464 | 2.35107 |
| UGP2 | 0.000412595 | 1.5974 |
| UHMK1 | 0.00699323 | 1.85703 |
| UHRF1 | 0.0457472 | 2.16866 |
| URB1 | 0.0145262 | 2.03379 |
| USP46 | 0.0231137 | 2.17269 |
| USP53 | 0.00509784 | 3.95977 |
| UTP15 | 0.0108825 | 1.72564 |
| UTP20 | 0.0124564 | 1.58824 |
| UXS1 | 0.00273033 | 1.95299 |
| VASP | 0.00323802 | 2.28178 |
| VCL | 0.000621915 | 2.82458 |
| VKORC1L1 | 2.71E-05 | 1.97813 |
| VNN2 | 0.0128795 | 2.01627 |
| VOPP1 | 0.00249233 | 1.54591 |
| VPS13D | 0.0429495 | 1.79283 |
| VWA2 | 0.00176041 | 1.51037 |
| WASF3 | 0.00332458 | 1.7278 |
| WDFY4 | 0.0298136 | 1.75859 |
| WDR1 | 0.000449409 | 2.72168 |
| WDR44 | 0.00364537 | 1.59656 |
| WDR77 | 0.0100729 | 1.73492 |
| WFDC1 | 0.00210018 | 5.90847 |
| WIPI1 | 0.00093688 | 1.70344 |
| WNT5B | 0.00099515 | 2.13679 |
| WSB2 | 0.00219031 | 1.88821 |
| WWC1 | 0.0465345 | 2.05701 |
| XDH | 0.00759102 | 3.39458 |
| XPO5 | 0.00884296 | 1.83757 |
| YDJC | 7.43E-05 | 1.71016 |
| YEATS2 | 0.00748287 | 1.53245 |
| YIF1B | 0.000655722 | 2.94369 |
| YRDC | 0.0146938 | 1.58268 |
| ZBTB16 | 4.33E-05 | 23.8266 |
| ZBTB42 | 0.00117188 | 1.70358 |
| ZCCHC6 | 0.00027581 | 2.11975 |
| ZDHHC12 | 0.000702237 | 1.61604 |
| ZDHHC13 | 0.00669367 | 1.91122 |
| ZDHHC23 | 0.00446687 | 2.01525 |
| ZFP36 | 0.0194782 | 1.56314 |
| ZNF215 | 0.00530908 | 2.35259 |
| ZNF225 | 0.0279926 | 1.79707 |
| ZNF259 | 0.00169597 | 1.56064 |
| ZNF259P1 | 0.02034 | 1.71514 |
| ZNF281 | 0.00215305 | 1.71667 |
| ZNF295 | 0.00327077 | 2.13485 |
| ZNF343 | 0.0404732 | 1.6323 |
| ZNF430 | 0.0375029 | 1.51342 |
| ZNF432 | 0.0459262 | 1.59095 |
| ZNF566 | 0.0137787 | 1.67998 |
| ZNF607 | 0.00260995 | 1.62153 |
| ZNF622 | 0.00408912 | 1.50939 |
| ZNF789 | 0.0350477 | 1.80242 |
| ZRANB1 | 0.0107293 | 1.72402 |
| ZSWIM4 | 0.00644639 | 1.84829 |
| ZWILCH | 0.0379457 | 1.56263 |
| ZWINT | 0.0201522 | 2.04311 |

**Additional Table 4: Down-regulated mRNAs in healthy ASM cells after stimulation with Dex + FCS.**

| **Gene Symbol** | ***P* value** | **Fold-Change** |
| --- | --- | --- |
| ABCA1 | 0.00337052 | -2.2686 |
| ABCA11P | 0.000885648 | -2.19856 |
| ABCA7 | 0.0111946 | -2.017 |
| ABCB4 | 0.0120505 | -2.35195 |
| ABCB7 | 0.000366452 | -1.51218 |
| ABCC6 | 0.000771714 | -2.19191 |
| ABHD8 | 0.00256582 | -1.58857 |
| ACBD6 | 0.00256778 | -1.63374 |
| ACCN1 | 0.00383215 | -2.34896 |
| ACER1 | 0.000169795 | -1.84891 |
| ACOX2 | 0.00150934 | -1.68802 |
| ACP5 | 0.00401377 | -1.78766 |
| ADAL | 0.0110089 | -2.52092 |
| ADAM8 | 0.00762295 | -2.39094 |
| ADAMTS10 | 0.00779171 | -2.33965 |
| ADAMTS14 | 0.0211776 | -3.25308 |
| ADAMTS19 | 0.0170727 | -2.93207 |
| ADAMTSL1 | 0.00151837 | -2.76771 |
| ADC | 0.0015464 | -1.57007 |
| ADORA1 | 0.0301814 | -1.93422 |
| ADRBK2 | 0.0102167 | -1.53306 |
| AF495723 | 0.00257062 | -1.73031 |
| AFAP1 | 0.002664 | -1.88496 |
| AGAP2 | 0.00629662 | -1.64559 |
| AGRN | 0.0281925 | -1.82739 |
| AGT | 0.0019503 | -2.81734 |
| AHCYL2 | 0.000783773 | -1.83182 |
| AHDC1 | 0.0016161 | -1.51131 |
| AHNAK2 | 0.0037012 | -1.79328 |
| AIF1L | 0.0118844 | -1.98126 |
| AK022088 | 0.00129155 | -2.11416 |
| AK027069 | 0.0412108 | -1.78243 |
| AK096443 | 0.0226167 | -1.87735 |
| AK1 | 0.00252646 | -1.55115 |
| AK123300 | 0.0185267 | -1.54377 |
| AK123701 | 0.0273962 | -1.79757 |
| AK123797 | 0.00858001 | -2.70192 |
| AK124041 | 0.0123434 | -1.56028 |
| AK124695 | 0.041622 | -1.81637 |
| AK127786 | 0.0242506 | -1.72119 |
| AK309505 | 0.0161061 | -1.75542 |
| AKAP12 | 0.0186715 | -2.13806 |
| AKR1C3 | 0.0105627 | -2.26377 |
| AKR1C4 | 0.0068812 | -2.30739 |
| ALDH1A3 | 0.000137725 | -8.00914 |
| ALDH3A1 | 0.0122779 | -1.90613 |
| ALDH3A2 | 0.000344357 | -2.65239 |
| AMOT | 0.00492928 | -3.16801 |
| AMOTL1 | 0.00613699 | -1.68216 |
| ANGPTL2 | 0.00064754 | -3.53816 |
| ANKRD29 | 0.00147194 | -1.73934 |
| ANO8 | 0.0230479 | -2.09302 |
| ANXA8L2 | 0.018033 | -2.03521 |
| APOBEC3C | 0.0116001 | -1.90658 |
| APOBEC3F | 0.0036601 | -1.62546 |
| APOC1 | 0.00150136 | -2.08556 |
| APPBP2 | 0.00316744 | -1.50443 |
| AQP3 | 0.0425903 | -2.33781 |
| ARHGAP12 | 0.00115108 | -1.53139 |
| ARHGAP20 | 0.0382488 | -1.62607 |
| ARHGAP25 | 0.0424844 | -2.93613 |
| ARHGAP28 | 0.000252692 | -2.71388 |
| ARHGAP33 | 0.000598097 | -1.77932 |
| ARL15 | 0.0298473 | -1.68281 |
| ARL3 | 0.00123021 | -1.75609 |
| ARL4C | 5.63E-05 | -3.13209 |
| ARMCX1 | 0.00713824 | -1.55124 |
| ARMCX4 | 0.0369871 | -2.29515 |
| ARVCF | 0.00240991 | -1.57659 |
| ASGR1 | 0.00226376 | -1.7347 |
| ASPRV1 | 0.0162836 | -2.61136 |
| ASS1 | 0.0156226 | -1.87312 |
| ATF5 | 0.015897 | -1.80906 |
| ATP6AP1L | 0.0220437 | -1.52592 |
| ATP6V1G2 | 0.0164818 | -1.80588 |
| ATP8A1 | 0.0310125 | -1.69646 |
| ATP8B4 | 0.0212205 | -2.39156 |
| ATXN1 | 0.00565847 | -1.50798 |
| AX746564 | 0.0193989 | -1.74332 |
| AX747437 | 0.00355847 | -2.82295 |
| AX747534 | 0.00154871 | -2.66579 |
| AX747640 | 0.00245415 | -1.51683 |
| AX747706 | 0.00673828 | -2.2807 |
| AX747985 | 0.015693 | -1.61768 |
| BAALC | 6.71E-06 | -4.75955 |
| BAI2 | 0.000152263 | -2.43183 |
| BBC3 | 0.000110239 | -2.78025 |
| BBS9 | 0.00946285 | -1.59927 |
| BC029890 | 0.000592533 | -1.54224 |
| BC030764 | 0.000184487 | -2.52454 |
| BC038245 | 0.000801876 | -2.14475 |
| BCL2L11 | 0.00240471 | -1.73896 |
| BDKRB1 | 2.62E-05 | -6.72818 |
| BDKRB2 | 0.000138215 | -4.41221 |
| BDNFOS | 0.0203705 | -2.49075 |
| BEX2 | 0.0118464 | -2.05121 |
| BEX4 | 0.0443985 | -1.58268 |
| BF515046 | 0.0122988 | -1.78234 |
| BHLHE23 | 0.00237403 | -2.45717 |
| BIN1 | 0.000793404 | -1.5919 |
| BM932296 | 0.00162232 | -1.56873 |
| BMF | 0.00337792 | -1.70098 |
| BNC2 | 0.00643962 | -1.90852 |
| BRSK1 | 0.015551 | -1.64799 |
| BST2 | 0.00307718 | -1.81463 |
| BTG2 | 3.40E-05 | -2.05116 |
| BTN3A1 | 0.00136524 | -1.70776 |
| BTN3A2 | 0.000864742 | -1.7893 |
| BX350880 | 0.0226841 | -1.518 |
| BX398892 | 0.0349141 | -2.13114 |
| C10orf105 | 0.0191875 | -3.96839 |
| C10orf107 | 0.00847668 | -2.71686 |
| C10orf41 | 0.00101238 | -1.78929 |
| C10orf58 | 0.0402076 | -1.63583 |
| C11orf20 | 0.00115245 | -1.79734 |
| C11orf45 | 0.0114687 | -1.89245 |
| C11orf65 | 0.015335 | -1.93641 |
| C12orf60 | 0.00282146 | -2.41332 |
| C12orf72 | 0.0292802 | -1.58155 |
| C13orf29 | 0.0165615 | -2.86904 |
| C14orf132 | 0.000594516 | -2.23189 |
| C14orf159 | 0.00166644 | -1.7563 |
| C14orf56 | 0.04845 | -1.86465 |
| C14orf93 | 0.00106239 | -1.74322 |
| C15orf51 | 3.87E-05 | -2.58886 |
| C16orf74 | 0.000963937 | -2.17841 |
| C16orf78 | 0.0219724 | -2.61697 |
| C16orf89 | 0.0211068 | -1.88119 |
| C17orf108 | 0.0284825 | -1.53172 |
| C17orf58 | 0.00981777 | -1.56584 |
| C17orf76 | 0.0141189 | -2.01531 |
| C18orf56 | 0.0142583 | -2.47479 |
| C19orf66 | 0.00518606 | -1.67991 |
| C1orf127 | 0.0354215 | -2.18449 |
| C1orf213 | 0.00214998 | -1.95217 |
| C1orf228 | 0.0164799 | -1.94906 |
| C1orf38 | 0.0336393 | -1.72612 |
| C1orf46 | 0.0280495 | -1.66044 |
| C1orf51 | 0.000177671 | -2.13717 |
| C1QTNF2 | 0.0386469 | -1.75727 |
| C1QTNF6 | 0.0467443 | -1.51655 |
| C20orf132 | 0.00526429 | -1.62815 |
| C20orf96 | 0.0111141 | -2.31478 |
| C21orf89 | 0.0179394 | -1.93909 |
| C21orf93 | 0.0212572 | -1.5614 |
| C22orf36 | 0.0257131 | -1.72443 |
| C2orf89 | 0.00466322 | -2.04469 |
| C3 | 0.0435044 | -2.2691 |
| C3orf17 | 0.00962176 | -1.60329 |
| C3orf67 | 0.0317316 | -2.47151 |
| C4orf23 | 0.000332533 | -1.5921 |
| C4orf3 | 8.95E-05 | -1.68757 |
| C5 | 0.0180758 | -1.53354 |
| C5orf39 | 0.00792934 | -1.50341 |
| C5orf4 | 0.00425001 | -2.26092 |
| C5orf41 | 0.00678904 | -1.54451 |
| C6orf122 | 0.00601451 | -2.51118 |
| C6orf134 | 0.0451178 | -1.62175 |
| C6orf154 | 0.00172184 | -1.76339 |
| C6orf225 | 0.000694659 | -1.64321 |
| C6orf26 | 0.0119206 | -2.18367 |
| C8orf46 | 0.00147974 | -2.76141 |
| C8orf56 | 0.0369702 | -1.66515 |
| C9orf150 | 0.00460496 | -1.58697 |
| C9orf24 | 0.0224534 | -1.69088 |
| C9orf7 | 0.000130562 | -2.12299 |
| C9orf96 | 0.0114176 | -2.15362 |
| CA12 | 0.0048683 | -2.6082 |
| CACNA1G | 0.00476279 | -1.85968 |
| CACNA1H | 0.0192063 | -1.76806 |
| CADM1 | 0.00124398 | -1.7406 |
| CAPN5 | 0.00944769 | -1.62304 |
| CAPS2 | 0.00120446 | -1.90755 |
| CARD16 | 0.0310048 | -1.79266 |
| CARHSP1 | 0.000809709 | -1.55181 |
| CASP1 | 0.00346745 | -1.87892 |
| CATSPERG | 0.00230859 | -1.53091 |
| CBR3 | 0.0272544 | -1.54374 |
| CCDC102A | 0.000185302 | -1.7661 |
| CCDC102B | 0.0212628 | -2.08209 |
| CCDC103 | 0.00214903 | -1.93837 |
| CCDC120 | 0.00210492 | -1.58726 |
| CCDC154 | 0.00807923 | -2.11914 |
| CCDC159 | 0.00685485 | -1.53204 |
| CCDC23 | 0.0013558 | -2.30564 |
| CCDC28B | 0.000117916 | -1.68127 |
| CCDC30 | 0.0198542 | -3.10704 |
| CCDC64B | 0.00384874 | -1.50755 |
| CCDC74B | 0.00577911 | -1.67098 |
| CCKAR | 0.00181059 | -4.84231 |
| CCL11 | 0.00275305 | -6.48161 |
| CCL25 | 0.0102757 | -1.50656 |
| CCL27 | 0.00559887 | -1.79484 |
| CCL7 | 0.0143492 | -5.0024 |
| CCNG1 | 0.000531589 | -1.7182 |
| CCR10 | 0.00272212 | -1.56278 |
| CCRL1 | 0.0468433 | -1.91019 |
| CD248 | 0.00975461 | -2.29119 |
| CD83 | 1.44E-05 | -1.90592 |
| CDC25B | 0.0105577 | -1.65758 |
| CDH10 | 0.0349263 | -2.30201 |
| CDH18 | 0.0125113 | -5.70963 |
| CDH6 | 0.00496136 | -1.76768 |
| CDK19 | 0.000237287 | -1.6998 |
| CDK4 | 0.00742969 | -1.78012 |
| CDKN1A | 0.0172356 | -1.64006 |
| CDKN1B | 0.000858265 | -1.52385 |
| CDKN2A | 0.000675705 | -1.60614 |
| CDKN2B | 0.00238006 | -2.80415 |
| CECR1 | 0.00802452 | -1.59221 |
| CELA2B | 0.0121577 | -1.67161 |
| CEP57 | 0.000601932 | -1.5963 |
| CES2 | 0.0482109 | -1.58752 |
| CES3 | 0.0132291 | -1.52948 |
| CFB | 0.0244355 | -2.29379 |
| CGNL1 | 0.0105675 | -2.82046 |
| CH25H | 0.00503571 | -2.76651 |
| CHADL | 0.00783214 | -2.0536 |
| CHDH | 0.00627893 | -2.63689 |
| CHI3L1 | 0.034569 | -1.50761 |
| CHN1 | 0.0476482 | -1.75398 |
| CHRDL2 | 0.00289354 | -4.14768 |
| CIB2 | 0.000433414 | -1.69771 |
| CIC | 0.000954268 | -1.87953 |
| CLDN11 | 1.85E-06 | -2.65429 |
| CLDN15 | 0.0375473 | -1.60702 |
| CLEC12B | 0.0102806 | -2.02889 |
| CLEC3B | 0.000319516 | -2.76221 |
| CLIC2 | 0.000931208 | -2.57635 |
| CLSTN3 | 0.0436014 | -1.67586 |
| CLU | 0.025391 | -1.6455 |
| CNIH3 | 0.000754758 | -4.99123 |
| CNNM2 | 0.0120326 | -2.00003 |
| CNTNAP1 | 0.028124 | -1.50458 |
| COL14A1 | 0.00122491 | -2.24037 |
| COL15A1 | 0.0149457 | -2.85384 |
| COL21A1 | 0.025119 | -3.7501 |
| COL24A1 | 0.000981071 | -1.69946 |
| COL3A1 | 0.0228396 | -1.7193 |
| COL4A5 | 0.00338073 | -2.25197 |
| COL9A2 | 0.0154467 | -1.70562 |
| COL9A3 | 0.00641954 | -1.56171 |
| CORO2B | 0.00163774 | -1.79361 |
| COX19 | 0.00848543 | -1.61115 |
| CPAMD8 | 0.0419549 | -1.76642 |
| CPE | 0.00290763 | -1.50564 |
| CPEB1 | 0.000355329 | -1.63234 |
| CPT1B | 0.0212668 | -1.84123 |
| CPZ | 0.000442315 | -2.51533 |
| CR620599 | 0.0151458 | -1.57561 |
| CR997556 | 0.0392782 | -1.83595 |
| CRABP2 | 8.22E-05 | -3.75362 |
| CREB5 | 0.000233237 | -5.18935 |
| CRTAP | 0.020497 | -1.62742 |
| CRTC1 | 0.00597529 | -2.09859 |
| CRYM | 0.00711076 | -2.17883 |
| CSDC2 | 0.00644284 | -2.20623 |
| CTSF | 0.00655489 | -1.55555 |
| CTTNBP2 | 0.00104365 | -4.19407 |
| CTXN1 | 0.0058725 | -1.70051 |
| CXCL12 | 0.000452315 | -6.28683 |
| CXCL2 | 0.0289296 | -3.14021 |
| CXCR7 | 0.0313813 | -1.53725 |
| CXXC5 | 0.000647246 | -1.5332 |
| CYP21A2 | 0.0128271 | -1.64932 |
| CYS1 | 0.00102963 | -2.06586 |
| D13069 | 0.0278227 | -1.96378 |
| DACH1 | 0.0348001 | -2.63748 |
| DACT1 | 0.00429698 | -1.70309 |
| DACT3 | 0.0001587 | -2.10431 |
| DB074148 | 0.0317826 | -1.7041 |
| DB462629 | 0.0291272 | -1.77873 |
| DBP | 0.00450142 | -2.91241 |
| DC378344 | 0.0100224 | -1.80651 |
| DCAF5 | 0.000285149 | -1.81702 |
| DCHS1 | 0.000544756 | -2.52577 |
| DCLK1 | 0.0162316 | -2.3005 |
| DCST1 | 0.000446572 | -4.11795 |
| DDAH2 | 0.00223249 | -1.77114 |
| DDB2 | 4.04E-05 | -2.3262 |
| DDIT3 | 0.0045193 | -2.25625 |
| DENND1B | 0.00765511 | -1.68418 |
| DENND5B | 6.09E-05 | -2.02463 |
| DERL3 | 0.00758762 | -1.60779 |
| DGKZ | 0.00158587 | -1.5781 |
| DHRS13 | 0.0208402 | -1.8596 |
| DIRC3 | 0.0216455 | -2.43501 |
| DLG3 | 0.000530165 | -1.85838 |
| DLL1 | 0.00579148 | -2.36025 |
| DNAJC18 | 0.0033564 | -1.52521 |
| DNALI1 | 0.0293495 | -1.77458 |
| DNM3 | 7.88E-05 | -3.25123 |
| DOCK4 | 0.00611504 | -1.71819 |
| DPH5 | 9.17E-06 | -1.56404 |
| DPP4 | 0.00222951 | -2.09634 |
| DPYSL3 | 0.00263308 | -1.65327 |
| DQ895628 | 0.000464735 | -3.254 |
| DRAM1 | 0.030297 | -1.55033 |
| DSTYK | 0.0150662 | -1.57794 |
| DTWD1 | 0.000318942 | -2.15685 |
| DYNLRB2 | 0.00903427 | -1.71974 |
| DYRK1B | 0.00611565 | -1.74354 |
| DZIP1 | 0.00862525 | -1.71538 |
| EDA2R | 2.27E-05 | -2.56093 |
| EFCAB7 | 0.000445856 | -2.13708 |
| EFNA4 | 0.00489262 | -1.62868 |
| EFS | 0.0019763 | -1.549 |
| EGFL8 | 0.0457236 | -1.8858 |
| EGR2 | 0.00458437 | -4.49407 |
| EHMT2 | 0.00365313 | -1.59591 |
| ELTD1 | 0.00918741 | -1.96426 |
| EMP3 | 0.0231007 | -1.6792 |
| ENST00000217162 | 0.0397611 | -1.88042 |
| ENST00000244064 | 0.0453771 | -1.54521 |
| ENST00000308537 | 0.0405365 | -1.7253 |
| ENST00000320486 | 0.0163686 | -1.66789 |
| ENST00000322069 | 0.0424166 | -1.51295 |
| ENST00000331733 | 0.00587405 | -1.76987 |
| ENST00000339692 | 0.023964 | -1.86796 |
| ENST00000355040 | 0.000169737 | -1.78253 |
| ENST00000360087 | 0.0384037 | -1.6727 |
| ENST00000372591 | 0.0039483 | -2.35334 |
| ENST00000374440 | 0.00056361 | -1.52949 |
| ENST00000376376 | 0.00794156 | -1.71983 |
| ENST00000379496 | 0.00188069 | -1.61428 |
| ENST00000390376 | 0.0161087 | -1.55896 |
| ENST00000398617 | 0.0384201 | -1.72205 |
| ENST00000399541 | 0.00393834 | -2.03754 |
| ENST00000399945 | 0.0363935 | -1.77031 |
| ENST00000402876 | 0.0188196 | -1.9447 |
| ENST00000402883 | 0.0139062 | -2.0783 |
| ENST00000403389 | 0.000191527 | -2.75665 |
| ENST00000409554 | 0.00140573 | -3.34977 |
| ENST00000422553 | 0.0175383 | -1.79365 |
| ENST00000423322 | 0.00100443 | -5.06723 |
| EPHA5 | 0.00107295 | -3.31584 |
| EPHB2 | 0.0152646 | -2.0534 |
| EPHB3 | 7.52E-05 | -2.53947 |
| EPHB4 | 0.000751913 | -1.62144 |
| EPHX2 | 0.000637089 | -3.07599 |
| EVI2A | 0.0178962 | -2.59138 |
| EVX1 | 0.00333994 | -1.70303 |
| F2RL1 | 0.00254996 | -2.15908 |
| FABP3 | 0.0251799 | -2.39624 |
| FAIM2 | 0.000147835 | -2.13225 |
| FAM102A | 0.0015431 | -1.79913 |
| FAM110B | 0.0385376 | -1.76952 |
| FAM117B | 0.00103758 | -2.19738 |
| FAM129A | 4.86E-05 | -2.51442 |
| FAM134B | 0.0365961 | -1.73196 |
| FAM135A | 0.0410155 | -1.85985 |
| FAM149A | 0.0135766 | -1.65966 |
| FAM155A | 0.00256612 | -1.56852 |
| FAM161A | 0.000925492 | -1.5928 |
| FAM171B | 0.0134506 | -2.3432 |
| FAM175A | 0.00155973 | -1.57311 |
| FAM38B | 0.00203182 | -2.09513 |
| FAM46A | 0.00383044 | -2.43886 |
| FAM46C | 7.23E-05 | -5.33877 |
| FAM53B | 0.00307352 | -1.50224 |
| FAM57B | 0.0422207 | -1.52344 |
| FAM92A1 | 0.00321603 | -1.52485 |
| FANCL | 0.0145891 | -1.5193 |
| FBXO15 | 0.00512161 | -2.59649 |
| FBXO2 | 0.00387801 | -1.6072 |
| FBXO22OS | 0.0109749 | -1.93022 |
| FBXO44 | 0.0399437 | -1.94098 |
| FCGR2A | 0.000562221 | -1.95465 |
| FDXR | 7.08E-05 | -2.70195 |
| FES | 0.0141475 | -1.57707 |
| FEZ1 | 0.0162331 | -1.51134 |
| FGD1 | 0.0217648 | -1.51976 |
| FGD2 | 0.00765489 | -2.58916 |
| FGF10 | 0.00648055 | -1.89953 |
| FGF13 | 0.0186112 | -2.34614 |
| FGF7 | 0.0120252 | -1.76582 |
| FIGF | 0.0149954 | -4.10689 |
| FIGN | 0.0100575 | -1.53206 |
| FKBP7 | 0.032511 | -1.62313 |
| FLJ10357 | 0.000125904 | -2.00548 |
| FLJ13197 | 0.0326032 | -1.95121 |
| FLJ22536 | 0.00679291 | -2.4172 |
| FLJ26484 | 0.0391919 | -1.59169 |
| FLJ32065 | 0.0016143 | -1.50076 |
| FLJ32810 | 0.00549195 | -1.5456 |
| FLJ37644 | 0.0332904 | -2.89107 |
| FLJ41309 | 0.0327837 | -1.52259 |
| FLJ41484 | 0.0202312 | -3.24312 |
| FLJ42875 | 0.030639 | -1.62179 |
| FLJ43860 | 0.0379684 | -1.69347 |
| FLJ44790 | 0.0257676 | -1.96851 |
| FLJ45445 | 0.035061 | -1.60657 |
| FLJ46020 | 0.0350244 | -1.67868 |
| FLJ46026 | 0.00212369 | -2.33951 |
| FLJ90757 | 0.00810829 | -1.63652 |
| FLRT3 | 0.000827879 | -3.2644 |
| FLT3LG | 0.0100126 | -2.05869 |
| FNDC1 | 0.0047965 | -2.23177 |
| FOLR1 | 7.05E-05 | -2.35061 |
| FOXA3 | 0.0401592 | -2.20383 |
| FOXC1 | 0.03546 | -1.55328 |
| FOXD4 | 0.00138077 | -3.76533 |
| FOXF2 | 0.000444525 | -2.64844 |
| FOXO6 | 0.0263252 | -1.75615 |
| FOXP1 | 2.68E-06 | -1.53347 |
| FOXQ1 | 0.0292757 | -1.88613 |
| FRRS1 | 0.0116003 | -1.5452 |
| FST | 0.000287369 | -3.62066 |
| FSTL5 | 0.000633362 | -6.5532 |
| FXYD1 | 0.0152238 | -2.65912 |
| FXYD6 | 0.00612532 | -2.71062 |
| FZD9 | 0.00704489 | -1.6189 |
| G0S2 | 0.00363924 | -13.4015 |
| GABARAP | 0.0170425 | -1.54456 |
| GADD45G | 0.00269684 | -1.72747 |
| GALM | 0.00665162 | -1.90874 |
| GALNT12 | 0.0394454 | -2.36154 |
| GALNTL1 | 0.00786483 | -3.18488 |
| GAMT | 0.000865823 | -1.84709 |
| GAS2L3 | 0.0196811 | -2.3332 |
| GBP2 | 0.000318496 | -1.93821 |
| GCK | 0.00348516 | -1.52417 |
| GDF1 | 0.00392669 | -4.09131 |
| GDF10 | 0.0208886 | -3.86823 |
| GDF15 | 0.000281714 | -4.19523 |
| GEM | 0.0366173 | -1.66325 |
| GFRA1 | 5.55E-06 | -2.95069 |
| GGA2 | 0.00816809 | -1.51914 |
| GGTLC1 | 0.00328187 | -4.71643 |
| GJA3 | 0.0416187 | -1.94838 |
| GJC2 | 0.000562772 | -2.02996 |
| GLI1 | 0.00103644 | -3.29422 |
| GLI3 | 0.000205785 | -2.40644 |
| GLIS1 | 0.00557174 | -1.95125 |
| GLT8D2 | 0.0070032 | -1.61418 |
| GNL1 | 6.20E-07 | -1.57552 |
| GPC2 | 0.0097156 | -1.99794 |
| GPER | 0.000561204 | -2.83988 |
| GPNMB | 0.00845031 | -1.55111 |
| GPR125 | 0.00762116 | -2.11176 |
| GPR126 | 0.0341779 | -1.6473 |
| GPR162 | 0.00109564 | -2.07823 |
| GPR56 | 0.000773367 | -4.45661 |
| GPR62 | 0.003395 | -2.53169 |
| GPR68 | 0.00273182 | -5.9318 |
| GPRASP1 | 0.0061896 | -3.51041 |
| GPRASP2 | 0.00369497 | -1.55886 |
| GPRC5C | 0.00444735 | -1.62563 |
| GPX4 | 0.0144544 | -1.50945 |
| GRASP | 0.000549756 | -1.99351 |
| GSTA4 | 0.00425848 | -1.56566 |
| GSTM1 | 0.00355207 | -1.52944 |
| GSTM2 | 0.0354705 | -1.95441 |
| GSTM4 | 0.0223911 | -1.77358 |
| GSTM5 | 0.00206982 | -1.97382 |
| GXYLT2 | 1.96E-05 | -2.83828 |
| GYPC | 0.016101 | -1.60302 |
| H2BFM | 0.0026544 | -2.37092 |
| H2BFXP | 0.00254832 | -1.61009 |
| HAAO | 0.0191601 | -1.9891 |
| HBA2 | 0.0041181 | -3.73433 |
| HCFC1R1 | 0.00395126 | -1.99782 |
| HDAC5 | 0.0311647 | -1.51067 |
| HEXDC | 0.00839458 | -1.6764 |
| HEXIM2 | 0.000519701 | -2.24541 |
| HGF | 0.000576521 | -2.5086 |
| HHATL | 0.000962141 | -2.58291 |
| HHLA2 | 0.0294009 | -1.54734 |
| HIST1H4E | 0.0189035 | -2.03295 |
| HIST1H4K | 0.019328 | -1.52565 |
| HMCN1 | 0.0145004 | -2.34348 |
| HMGA1 | 0.0460645 | -1.65135 |
| HMGB3 | 0.0240097 | -1.53715 |
| HMHB1 | 0.0224203 | -1.5889 |
| HMOX1 | 0.00228393 | -1.59069 |
| HOXA2 | 0.00301885 | -2.74485 |
| HOXA3 | 0.00461775 | -1.9177 |
| HOXA4 | 0.00768808 | -1.60277 |
| HOXA5 | 0.00241968 | -1.6642 |
| HOXA6 | 0.0101962 | -1.52678 |
| HRH1 | 0.00246925 | -1.7906 |
| HSD11B2 | 0.0485652 | -3.71856 |
| HSD17B14 | 0.00086373 | -1.78142 |
| HSD17B6 | 0.00122308 | -3.69949 |
| HSP90AA5P | 0.00263235 | -1.74792 |
| HSPA12A | 0.000746952 | -2.08121 |
| HSPA12B | 0.00221542 | -3.30022 |
| HSPA4L | 0.00481015 | -3.95714 |
| HSPB9 | 0.00471674 | -1.70667 |
| HTR2B | 0.0138081 | -2.9556 |
| HTRA3 | 0.02973 | -2.79256 |
| HVCN1 | 0.0137971 | -1.6441 |
| ICAM4 | 0.0145841 | -1.55441 |
| ICOSLG | 0.0146076 | -1.71977 |
| IER3 | 0.0102621 | -2.11286 |
| IFFO1 | 0.00516284 | -1.67136 |
| IFI16 | 0.000933594 | -1.9876 |
| IFI30 | 0.00816339 | -1.73515 |
| IFI44 | 0.00294266 | -2.13683 |
| IFIT1 | 3.66E-06 | -4.24405 |
| IFIT2 | 0.002855 | -1.94848 |
| IFITM1 | 0.0219928 | -1.68116 |
| IFT140 | 0.0388304 | -1.71271 |
| IFT88 | 1.83E-05 | -1.86655 |
| IGDCC4 | 0.000445458 | -3.16309 |
| IGLL1 | 0.0432645 | -1.81997 |
| IGSF10 | 0.00414852 | -6.21351 |
| IL17RD | 0.000357851 | -2.72761 |
| IL32 | 0.00998055 | -2.07036 |
| IL4I1 | 0.000204087 | -7.67506 |
| ING4 | 0.0246156 | -1.55443 |
| INO80B | 0.0363666 | -1.62207 |
| IRF7 | 0.0483305 | -1.55338 |
| ISG20 | 0.000106868 | -3.64537 |
| ISLR | 0.00197344 | -1.81762 |
| ISYNA1 | 0.00592609 | -1.89084 |
| ITGA11 | 0.00102467 | -2.05107 |
| ITGA7 | 0.00203446 | -1.91225 |
| ITGA9 | 0.0402751 | -1.58119 |
| ITGB4 | 0.00043152 | -2.79057 |
| ITGB8 | 0.00183318 | -2.32187 |
| ITIH4 | 0.0257579 | -1.67997 |
| ITIH5 | 0.00457502 | -2.46853 |
| ITPKB | 0.00418384 | -2.40723 |
| JMJD7-PLA2G4B | 0.049032 | -1.71923 |
| JMY | 0.00212793 | -1.63709 |
| KATNAL2 | 0.0240768 | -1.63692 |
| KBTBD10 | 0.0316675 | -2.60332 |
| KCNA3 | 0.0100279 | -1.81704 |
| KCNE3 | 0.00333633 | -3.5031 |
| KCNK4 | 0.00692462 | -2.57256 |
| KCNMA1 | 0.00031283 | -2.35879 |
| KCTD12 | 0.00322643 | -4.44005 |
| KIAA0895L | 0.0314687 | -1.82547 |
| KIAA1009 | 0.0135572 | -1.54542 |
| KIAA1324 | 0.00884601 | -1.72442 |
| KIAA1324L | 0.00625483 | -2.24698 |
| KIAA1407 | 0.00961827 | -2.48342 |
| KIAA1549 | 0.0461066 | -1.67266 |
| KIAA1683 | 0.00297744 | -2.33816 |
| KIAA1908 | 0.0165017 | -1.57888 |
| KILLIN | 0.0318246 | -2.16461 |
| KIRREL3 | 0.0359543 | -1.67007 |
| KLF11 | 0.00160687 | -1.56423 |
| KLF12 | 0.00649048 | -1.53357 |
| KLF4 | 0.000228195 | -2.24482 |
| KLHDC7B | 0.00203235 | -3.81682 |
| KLHL3 | 0.0128552 | -2.59325 |
| KLKB1 | 0.0456935 | -1.83836 |
| KRT85 | 0.00413376 | -2.12471 |
| KRTAP19-2 | 0.0142807 | -1.67966 |
| LAMA4 | 3.14E-05 | -1.61635 |
| LAMB2L | 0.0374147 | -1.64702 |
| LAMB3 | 0.000800244 | -2.31597 |
| LARP6 | 3.05E-05 | -1.91153 |
| LASS1 | 0.0108901 | -4.23167 |
| LCE3B | 0.0285989 | -1.96675 |
| LDB2 | 0.000167332 | -3.72349 |
| LETMD1 | 0.00289122 | -1.68963 |
| LGI4 | 0.0308762 | -2.21822 |
| LGR4 | 0.00020215 | -2.15991 |
| LHFPL2 | 0.000201426 | -2.20369 |
| LIAS | 0.00261389 | -1.69994 |
| LIF | 0.00791231 | -3.05818 |
| LIN7B | 0.000801539 | -1.60087 |
| LIPC | 0.00318023 | -4.24995 |
| LNP1 | 0.000841802 | -1.78969 |
| LOC100049716 | 0.010993 | -1.54488 |
| LOC100128055 | 0.00864536 | -2.30443 |
| LOC100128184 | 0.00991274 | -1.65363 |
| LOC100128511 | 0.043268 | -1.76717 |
| LOC100128913 | 0.0447069 | -1.68054 |
| LOC100128934 | 0.00910616 | -2.63889 |
| LOC100129395 | 0.0126727 | -1.55159 |
| LOC100129447 | 0.0345289 | -1.52846 |
| LOC100130000 | 0.000774919 | -2.06841 |
| LOC100130111 | 0.000291899 | -2.4768 |
| LOC100130360 | 0.00262571 | -2.10303 |
| LOC100130453 | 0.00556035 | -2.18927 |
| LOC100130557 | 0.00399248 | -2.04994 |
| LOC100131326 | 0.0197735 | -1.53096 |
| LOC100131355 | 0.0070759 | -1.66267 |
| LOC100132167 | 0.00248085 | -1.52112 |
| LOC100132273 | 0.00821766 | -1.5241 |
| LOC100132649 | 0.0399879 | -1.63447 |
| LOC100132891 | 0.00743118 | -1.82092 |
| LOC100133050 | 0.0168327 | -1.64333 |
| LOC100133070 | 0.0136393 | -1.76504 |
| LOC100144602 | 1.95E-05 | -1.86852 |
| LOC100170939 | 0.0201065 | -1.83966 |
| LOC100272228 | 0.0294807 | -1.83813 |
| LOC100287428 | 0.00421506 | -1.75689 |
| LOC100288412 | 0.00478534 | -2.17072 |
| LOC143286 | 0.000724152 | -1.69314 |
| LOC144571 | 0.000715501 | -2.15287 |
| LOC145945 | 0.0493437 | -1.83366 |
| LOC147646 | 0.00629841 | -2.72097 |
| LOC157562 | 0.00857153 | -2.05346 |
| LOC282997 | 0.000124508 | -2.27708 |
| LOC283392 | 0.0293105 | -1.53031 |
| LOC283454 | 0.0256914 | -1.78732 |
| LOC283904 | 0.0245011 | -1.61977 |
| LOC284276 | 0.00153139 | -2.40191 |
| LOC284408 | 0.00074867 | -2.23401 |
| LOC285141 | 0.000154318 | -3.43267 |
| LOC285147 | 0.0357144 | -1.92295 |
| LOC286052 | 0.00196442 | -1.63879 |
| LOC339192 | 0.0220657 | -2.66666 |
| LOC375010 | 0.0134908 | -1.52094 |
| LOC388242 | 0.0136126 | -1.95682 |
| LOC388588 | 0.0213926 | -1.80366 |
| LOC389033 | 0.0157668 | -1.63995 |
| LOC401387 | 0.0199289 | -1.59626 |
| LOC401561 | 0.0172808 | -1.52638 |
| LOC554202 | 0.019159 | -1.73469 |
| LOC55908 | 0.000944365 | -4.14777 |
| LOC572558 | 0.0102366 | -2.99751 |
| LOC642340 | 0.0094147 | -2.20942 |
| LOC644277 | 0.0136501 | -1.53359 |
| LOC645427 | 0.00821307 | -1.50587 |
| LOC651721 | 0.00958862 | -1.90199 |
| LOC653391 | 0.0410683 | -1.87669 |
| LOC678655 | 0.0476184 | -1.52697 |
| LOC727721 | 0.0275509 | -1.65247 |
| LOC727869 | 0.000285531 | -2.00146 |
| LOC728392 | 8.61E-05 | -3.42297 |
| LOC729013 | 0.00562779 | -1.64002 |
| LOC729558 | 0.00932955 | -1.55925 |
| LOC730235 | 0.00627293 | -1.73887 |
| LOC80154 | 3.08E-05 | -2.00672 |
| LOC90110 | 0.00211373 | -1.56252 |
| LOC92973 | 0.0129598 | -2.02303 |
| LOH12CR1 | 0.0161339 | -1.5222 |
| LOXL1 | 0.00179634 | -1.77154 |
| LPIN1 | 0.00993043 | -1.5736 |
| LPPR4 | 0.00578132 | -2.0316 |
| LRDD | 0.000685076 | -2.6339 |
| LRP4 | 0.040302 | -1.80701 |
| LRRC15 | 0.0467885 | -2.21633 |
| LRRC17 | 0.000460846 | -4.18532 |
| LRRC20 | 0.000369806 | -1.86089 |
| LRRC46 | 0.0328257 | -1.5232 |
| LRRC49 | 2.61E-05 | -1.96794 |
| LRRN3 | 0.00125394 | -6.81565 |
| LRRN4CL | 0.0229621 | -2.23285 |
| LSAMP | 0.0276735 | -1.61285 |
| LSP1 | 0.0200238 | -1.53104 |
| LTBP4 | 0.00253352 | -1.76143 |
| LUM | 0.000188343 | -3.54602 |
| LY96 | 0.000108646 | -1.56802 |
| MAF | 0.00222066 | -2.50333 |
| MAFB | 0.001664 | -3.57235 |
| MAL | 0.0439612 | -3.05508 |
| MAMSTR | 0.00461417 | -1.74252 |
| MAP2K6 | 0.000866033 | -2.6668 |
| MAP3K4 | 0.00269569 | -1.57115 |
| MAPK10 | 0.000719849 | -2.34631 |
| MAPK8IP1 | 0.00205398 | -2.01349 |
| MAPRE2 | 0.00267757 | -1.68295 |
| MAPT | 0.0155719 | -2.08352 |
| MARCKS | 0.00427427 | -1.73791 |
| MARCKSL1 | 0.00121732 | -3.59932 |
| MAST4 | 0.00129612 | -1.91461 |
| MCCD1 | 0.0430965 | -1.91367 |
| MDGA1 | 0.00673419 | -1.64775 |
| MDK | 0.00193607 | -2.01919 |
| ME3 | 0.00272461 | -1.5856 |
| MEIS1 | 0.00174526 | -1.51691 |
| METTL12 | 0.000990385 | -1.64517 |
| MEX3A | 0.0272621 | -3.12419 |
| MEX3B | 0.00156926 | -3.24133 |
| MFAP2 | 0.0227491 | -1.67242 |
| MFAP4 | 0.0017428 | -1.92849 |
| MGC2848 | 0.00411277 | -3.12983 |
| MGC31957 | 0.0040959 | -1.85631 |
| MGC42105 | 0.0085945 | -2.31365 |
| MIB2 | 0.00370444 | -1.5535 |
| MICALL2 | 0.00919847 | -1.64935 |
| MIR155HG | 0.00589078 | -3.26779 |
| MKL2 | 0.00111121 | -1.93392 |
| MMP1 | 0.0197453 | -4.59366 |
| MMP11 | 0.0146734 | -1.95176 |
| MMP23B | 0.00218374 | -2.26866 |
| MOXD1 | 0.000658486 | -1.96759 |
| MPP2 | 0.01818 | -1.51498 |
| MRPS6 | 0.000531634 | -1.8743 |
| MSC | 0.0174744 | -3.87016 |
| MST1 | 0.0129279 | -1.56405 |
| MSX2P1 | 0.00368136 | -2.11954 |
| MTHFR | 0.0170215 | -1.63954 |
| MTMR9L | 0.0226235 | -2.2434 |
| MUC8 | 0.023118 | -2.12891 |
| MUSTN1 | 0.0249631 | -2.62836 |
| MX1 | 0.0031986 | -3.10733 |
| MX2 | 0.00495057 | -2.40438 |
| MXD4 | 0.00186996 | -1.84579 |
| MXI1 | 0.00162164 | -1.5043 |
| MXRA5 | 0.000112787 | -6.61196 |
| MYL6B | 0.0025835 | -1.52491 |
| MYLIP | 0.00795135 | -2.76891 |
| MYO1B | 0.00742986 | -1.52952 |
| MYO7B | 0.00517743 | -2.49059 |
| MYOM2 | 0.0267751 | -1.78304 |
| MZF1 | 0.0330306 | -1.53979 |
| NAB1 | 0.0154853 | -1.5143 |
| NAIP | 0.013549 | -2.37168 |
| NBEA | 0.00273455 | -1.75208 |
| NBPF15 | 0.0221718 | -1.77516 |
| NCKAP5 | 0.0214598 | -3.05655 |
| NCRNA00118 | 0.0127293 | -1.57556 |
| NCRNA00219 | 0.000542009 | -1.59712 |
| NDRG4 | 0.00376791 | -1.63609 |
| NEDD4L | 2.63E-05 | -3.676 |
| NEK10 | 0.000918758 | -2.11129 |
| NEK11 | 0.0142911 | -1.67663 |
| NES | 0.00221364 | -1.67106 |
| NEURL | 0.0220917 | -1.58502 |
| NEURL1B | 0.0457137 | -1.6499 |
| NEURL2 | 0.00304548 | -1.81009 |
| NFATC4 | 0.000643829 | -2.5359 |
| NFE2 | 0.00236413 | -3.31941 |
| NFIX | 0.00722297 | -1.66601 |
| NGF | 0.0455115 | -1.59465 |
| NHS | 0.0125752 | -1.68811 |
| NHSL2 | 0.0258492 | -1.58343 |
| NINJ1 | 9.70E-05 | -2.09874 |
| NINL | 0.00397874 | -1.81979 |
| NIPAL2 | 0.00475631 | -1.5245 |
| NIPSNAP1 | 0.00115861 | -1.59339 |
| NLGN1 | 0.0275781 | -1.56964 |
| NNAT | 0.03197 | -1.9258 |
| NOTCH3 | 0.00925623 | -2.34492 |
| NOTCH4 | 0.00338619 | -1.67581 |
| NOV | 0.000238284 | -6.57305 |
| NOVA1 | 0.000169011 | -1.92536 |
| NOXA1 | 0.0349419 | -1.82497 |
| NR0B1 | 0.0028387 | -3.47758 |
| NR1D1 | 0.0105332 | -1.59742 |
| NR3C1 | 6.15E-05 | -2.3011 |
| NR3C2 | 0.00652751 | -1.61346 |
| NRG1 | 0.0075969 | -3.00225 |
| NRM | 0.0165772 | -1.80243 |
| NRN1L | 0.00770786 | -1.88966 |
| NTM | 0.0115725 | -1.9089 |
| NTN3 | 0.0360317 | -1.85486 |
| NXPH4 | 0.0011612 | -3.19793 |
| NYNRIN | 0.00197258 | -2.17916 |
| OAF | 0.0383377 | -1.66624 |
| OGN | 0.00331776 | -2.97154 |
| OLFML1 | 0.000478361 | -3.204 |
| OLFML2A | 0.000389544 | -3.88829 |
| OLFML2B | 0.00343207 | -1.77123 |
| OPRL1 | 0.0044286 | -1.57771 |
| OR10P1 | 0.0449478 | -1.53738 |
| OR4K14 | 0.00970112 | -2.1812 |
| OR6K2 | 0.0493655 | -1.6713 |
| OR6Y1 | 0.00691578 | -1.88455 |
| OSBPL7 | 0.00729358 | -2.13269 |
| OSR2 | 0.000551179 | -3.60693 |
| OXER1 | 0.00297574 | -2.79366 |
| P2RX7 | 0.00209581 | -2.59779 |
| P4HA2 | 0.0027391 | -1.71428 |
| PACSIN3 | 0.0396374 | -1.51932 |
| PAFAH1B3 | 0.00178992 | -2.05074 |
| PAG1 | 0.0227027 | -1.58654 |
| PALM | 0.00116546 | -1.74077 |
| PAQR8 | 0.0158735 | -1.91942 |
| PARD6G | 0.0110255 | -2.47016 |
| PARP10 | 0.00315719 | -1.65689 |
| PARP14 | 0.0014606 | -1.60055 |
| PARP9 | 0.0315483 | -1.63645 |
| PAX8 | 0.0120458 | -1.57163 |
| PBX1 | 0.00246614 | -2.14758 |
| PBX3 | 0.0159351 | -1.55284 |
| PCDH18 | 0.00539425 | -1.7884 |
| PCDHB11 | 0.00612457 | -1.68966 |
| PCDHB14 | 0.00449753 | -2.56666 |
| PCDHB9 | 0.00792514 | -2.17716 |
| PCNXL2 | 0.000240408 | -3.312 |
| PCOLCE | 0.000965533 | -2.03994 |
| PCOTH | 0.0160149 | -1.89642 |
| PCSK4 | 0.00828006 | -1.65734 |
| PDCD4 | 0.00330088 | -2.30954 |
| PDE5A | 0.000730071 | -2.08728 |
| PDE7B | 0.0296554 | -1.6701 |
| PDGFC | 0.0153334 | -1.51348 |
| PDK2 | 0.0225559 | -1.61916 |
| PER3 | 0.0079978 | -2.24513 |
| PFN4 | 0.0344602 | -1.50601 |
| PGA3 | 0.00135848 | -2.8682 |
| PGM5 | 0.00798711 | -1.71679 |
| PGPEP1 | 0.00495742 | -1.68556 |
| PHACTR1 | 2.75E-05 | -1.99391 |
| PHC1 | 0.00300601 | -1.54639 |
| PHEX | 0.00511478 | -2.13194 |
| PHF1 | 0.0169093 | -1.59079 |
| PHF21A | 0.0119716 | -1.6336 |
| PHGDH | 0.00202365 | -2.92031 |
| PHLDA1 | 0.0276089 | -1.65337 |
| PHLDB3 | 0.000194634 | -2.0952 |
| PIK3C2B | 0.0234532 | -2.51522 |
| PIM1 | 0.00796185 | -1.6673 |
| PIP5KL1 | 0.000399187 | -2.27488 |
| PITPNM2 | 0.0280612 | -1.50789 |
| PITX1 | 2.65E-05 | -1.82927 |
| PKIA | 0.00135418 | -1.88719 |
| PKP3 | 0.000157652 | -4.81616 |
| PLA2G4C | 0.00715849 | -2.04206 |
| PLAU | 0.0100163 | -2.32725 |
| PLCD4 | 0.000755133 | -1.93577 |
| PLEKHA6 | 0.0415774 | -1.72858 |
| PLEKHG1 | 0.0450264 | -1.87302 |
| PLEKHG4 | 0.000709846 | -1.99657 |
| PLK1S1 | 0.00582212 | -1.95044 |
| PLTP | 0.00125174 | -2.20183 |
| PLXDC1 | 0.000106486 | -2.4764 |
| PLXNA1 | 0.0266608 | -1.67897 |
| PLXNB2 | 0.00933369 | -1.5538 |
| PMEPA1 | 0.0288022 | -1.74933 |
| PNMA5 | 0.014913 | -1.52639 |
| PNRC1 | 0.00448918 | -1.82538 |
| POLL | 0.00125751 | -1.67633 |
| POSTN | 0.00773519 | -2.32251 |
| POU6F1 | 0.0269239 | -1.58969 |
| PPAP2A | 0.0117931 | -2.22256 |
| PPAP2B | 0.00664958 | -1.89839 |
| PPAP2C | 0.00387186 | -1.76521 |
| PPM1D | 7.16E-05 | -1.66032 |
| PPP1R9B | 0.0237325 | -1.5028 |
| PRDM16 | 0.0106143 | -2.0447 |
| PRDM8 | 0.00204149 | -1.57569 |
| PRKCE | 0.000751323 | -2.34311 |
| PRKG1 | 0.00827243 | -2.06131 |
| ProSAPiP1 | 0.0326525 | -1.52488 |
| PRRG3 | 0.000241954 | -4.34569 |
| PRRT1 | 0.0130564 | -1.99095 |
| PRRT2 | 0.00794161 | -3.91347 |
| PRRX1 | 0.00149635 | -2.06272 |
| PRSS23 | 0.0221555 | -1.90674 |
| PRSS35 | 0.000284361 | -6.43722 |
| PRSS36 | 0.0132375 | -1.66526 |
| PRTFDC1 | 2.68E-05 | -1.72364 |
| PSCA | 0.00822062 | -2.82868 |
| PSD3 | 0.00225987 | -1.65775 |
| PSIP1 | 7.91E-05 | -1.79274 |
| PSMB9 | 0.00596274 | -1.54003 |
| PTBP2 | 0.0298904 | -1.60295 |
| PTDSS1 | 0.0053683 | -1.65471 |
| PTEN | 0.00124112 | -1.72218 |
| PTGER1 | 0.0028151 | -2.1194 |
| PTGFR | 0.00766231 | -2.00326 |
| PTGFRN | 0.0024098 | -3.1283 |
| PTGIS | 0.0294245 | -1.70447 |
| PTN | 0.00322103 | -1.6491 |
| PTOV1 | 0.00499393 | -1.54331 |
| PTPRS | 7.70E-05 | -1.86196 |
| PTPRU | 0.00511925 | -1.75828 |
| PURG | 0.00408091 | -1.87057 |
| PXK | 0.018806 | -1.72859 |
| PYROXD2 | 0.0229179 | -1.86132 |
| QPRT | 5.67E-05 | -3.23514 |
| RAB26 | 0.00695707 | -3.59473 |
| RAB33A | 0.00534555 | -2.914 |
| RAB3IL1 | 0.0115622 | -1.57609 |
| RAB40B | 0.000223648 | -1.52633 |
| RAB7B | 0.000188577 | -4.67543 |
| RALGDS | 0.000346466 | -1.54409 |
| RALGPS1 | 0.0273207 | -1.79464 |
| RARRES2 | 0.0414856 | -1.54264 |
| RARRES3 | 0.00457411 | -2.48674 |
| RASA4 | 0.00211574 | -1.9177 |
| RASGEF1A | 0.0147473 | -2.16113 |
| RASGRF2 | 0.0234962 | -2.1295 |
| RASL12 | 0.0143048 | -2.16281 |
| RASSF2 | 0.00103154 | -2.2457 |
| RASSF4 | 0.0267989 | -1.56785 |
| RBM4B | 0.00457046 | -1.53602 |
| RBMX2 | 0.00130211 | -1.59182 |
| RBP1 | 0.00560512 | -2.1517 |
| RCAN2 | 0.0258726 | -3.23056 |
| RCOR2 | 0.00308221 | -2.39521 |
| RDH5 | 0.00156386 | -3.70741 |
| RELB | 0.00265356 | -2.26958 |
| RFTN2 | 0.00202341 | -1.86767 |
| RFX2 | 0.0133072 | -1.87969 |
| RFX7 | 0.000320461 | -1.78556 |
| RGN | 0.00502041 | -1.81362 |
| RGS10 | 0.000873927 | -2.00413 |
| RGS12 | 0.00368098 | -1.586 |
| RGS20 | 0.0249518 | -1.56696 |
| RHOJ | 8.99E-05 | -2.11338 |
| RIBC1 | 0.00183561 | -1.77414 |
| RIN1 | 0.0407023 | -1.53661 |
| RNASET2 | 0.00264859 | -1.5709 |
| RND2 | 0.00339313 | -5.06339 |
| RNF112 | 0.00225086 | -1.78257 |
| RNF122 | 0.00666338 | -2.44886 |
| RNF150 | 0.0330287 | -1.60509 |
| RNF175 | 0.0474874 | -2.1546 |
| RNF208 | 0.000334897 | -1.76005 |
| RNU12 | 0.0313932 | -1.72533 |
| ROBO1 | 0.00111584 | -2.36531 |
| RPS6KA5 | 0.000960797 | -3.76008 |
| RPS6KA6 | 0.0447226 | -1.55095 |
| RSL24D1P3 | 0.0377094 | -1.92761 |
| RTN4RL1 | 0.00449849 | -1.69875 |
| RTP4 | 0.0372448 | -1.68832 |
| RUNX1T1 | 0.0122275 | -1.66316 |
| RXFP3 | 0.0321962 | -1.97576 |
| S100A2 | 0.000873983 | -1.66638 |
| S100A4 | 0.00185081 | -1.61493 |
| SAC3D1 | 0.0234585 | -1.59684 |
| SACS | 0.028433 | -1.78103 |
| SALL2 | 0.00114819 | -2.4243 |
| SAMD11 | 0.0127007 | -1.58971 |
| SAMD12 | 0.000374573 | -2.93684 |
| SAMD5 | 0.00226624 | -2.8496 |
| SAT2 | 0.00177676 | -1.52343 |
| SATB1 | 0.00566849 | -1.59082 |
| SBNO2 | 0.00486433 | -1.54904 |
| SCARNA10 | 0.0265661 | -2.01923 |
| SCG5 | 0.0249126 | -1.59649 |
| SCN2A | 0.00566186 | -2.16889 |
| SCN2B | 2.62E-05 | -3.35911 |
| SCN4B | 0.000866862 | -1.68537 |
| SEC1 | 0.00513463 | -3.51461 |
| SECTM1 | 0.00285394 | -2.64429 |
| SELENBP1 | 0.000184701 | -2.55775 |
| SEMA3A | 0.013957 | -2.65204 |
| SEMA3B | 0.000103622 | -3.25848 |
| SEMA3F | 0.0119077 | -1.88944 |
| SENP7 | 0.0107115 | -1.66 |
| SERPINE2 | 0.0170663 | -1.59405 |
| SERPINF1 | 0.0173761 | -1.58447 |
| SERTAD2 | 0.0126827 | -1.52287 |
| SESN2 | 0.0329101 | -1.72575 |
| SESN3 | 0.0187793 | -1.97944 |
| SETBP1 | 0.0362086 | -1.50037 |
| SH2B2 | 0.0109488 | -2.09179 |
| SH3BP5 | 0.044099 | -1.7395 |
| SH3GL3 | 0.038684 | -1.98545 |
| SHANK3 | 0.0140874 | -1.77878 |
| SHC2 | 0.000514032 | -2.23094 |
| SHF | 0.0338104 | -1.84102 |
| SIPA1L2 | 0.00280949 | -6.3882 |
| SIRPB1 | 0.000435815 | -1.50985 |
| SIRT4 | 0.00470612 | -2.98639 |
| SIX5 | 0.00116569 | -2.00291 |
| SLC13A3 | 0.00135512 | -1.87558 |
| SLC25A23 | 0.00351174 | -1.60059 |
| SLC27A3 | 0.0444639 | -1.51085 |
| SLC2A12 | 0.0267872 | -2.73605 |
| SLC2A4RG | 0.000219488 | -1.68404 |
| SLC35E2 | 0.00465198 | -1.76674 |
| SLC45A3 | 0.017139 | -1.72284 |
| SLC5A3 | 0.00179598 | -2.08998 |
| SLC6A9 | 0.00234784 | -5.05247 |
| SLC7A11 | 0.0312957 | -1.77331 |
| SLC7A14 | 2.92E-05 | -4.07478 |
| SLC9A7 | 0.00114714 | -1.61441 |
| SLC9A9 | 0.00226874 | -2.22908 |
| SLIT3 | 0.0448749 | -1.5683 |
| SLITRK6 | 0.0127975 | -6.72413 |
| SMAD1 | 0.000378468 | -2.46003 |
| SMAD3 | 0.00791425 | -1.51937 |
| SMAD5OS | 0.0106048 | -2.49934 |
| SMO | 0.000614542 | -3.17209 |
| SMOX | 0.00147228 | -2.77216 |
| SNAP91 | 0.00450094 | -1.5319 |
| SNAR-G1 | 0.0346137 | -2.22719 |
| SNHG7 | 0.00172345 | -1.7611 |
| SNHG8 | 0.000586301 | -1.64296 |
| SNX21 | 0.000344363 | -2.24426 |
| SNX30 | 0.00920782 | -1.54109 |
| SOBP | 0.0374233 | -1.60208 |
| SOCS1 | 0.00530843 | -2.37897 |
| SOCS2 | 0.0326779 | -1.76941 |
| SOCS3 | 0.0126105 | -2.17176 |
| SOD2 | 0.049056 | -1.95525 |
| SORL1 | 0.00516461 | -2.32197 |
| SOX4 | 0.00113342 | -4.67803 |
| SP3 | 0.00874532 | -1.53636 |
| SP5 | 0.0144951 | -2.71837 |
| SPAG8 | 0.0177881 | -2.11676 |
| SPATA18 | 0.00014483 | -2.45347 |
| SPATA7 | 0.0189819 | -1.57545 |
| SPEG | 0.0144484 | -1.76708 |
| SPIRE2 | 0.000429287 | -1.67665 |
| SPNS3 | 0.00515002 | -1.88591 |
| SPON2 | 0.00422607 | -1.89803 |
| SPTBN4 | 0.0127067 | -1.69535 |
| SPTLC3 | 0.00814044 | -3.26956 |
| SSBP4 | 0.00057015 | -1.58353 |
| SSC5D | 0.000706815 | -2.64854 |
| SSX2IP | 0.0153001 | -1.63718 |
| ST3GAL1 | 0.00350362 | -1.52312 |
| ST8SIA1 | 0.000222241 | -3.29701 |
| STARD10 | 0.00122739 | -2.13502 |
| STMN1 | 0.0436972 | -1.55882 |
| STOX2 | 0.0087824 | -2.6422 |
| SULF2 | 0.000296232 | -3.40861 |
| SULT1A2 | 0.0332803 | -1.5157 |
| SUV420H2 | 0.0121281 | -1.60937 |
| SYNPO | 0.00346081 | -1.94274 |
| SYT7 | 0.00837125 | -3.99296 |
| TAC3 | 0.00679401 | -2.99392 |
| TARSL2 | 0.00597059 | -2.82516 |
| TAS2R50 | 0.0207405 | -2.00074 |
| TBC1D19 | 0.00495959 | -1.63521 |
| TBX1 | 0.0174068 | -1.78198 |
| TBX10 | 0.00705472 | -1.61272 |
| TBX5 | 0.00283476 | -1.7672 |
| TCEA1 | 0.000103772 | -1.57448 |
| TCEA2 | 0.00586603 | -1.51856 |
| TCEA3 | 0.000367823 | -2.77167 |
| TCEAL7 | 0.00506363 | -1.6743 |
| TCF3 | 0.000346163 | -1.82579 |
| TCF4 | 0.000383117 | -1.66457 |
| TCF7 | 0.01363 | -1.62861 |
| THAP9 | 0.0121233 | -2.18468 |
| THBS4 | 0.00281251 | -2.04947 |
| THC2485300 | 0.00139514 | -2.3434 |
| THC2564554 | 0.0154105 | -1.54386 |
| THC2570319 | 0.0248244 | -1.87002 |
| THC2598564 | 0.0015877 | -1.62882 |
| THC2614422 | 0.00435323 | -1.99481 |
| THC2647746 | 0.00465474 | -1.62739 |
| THC2655610 | 0.0030673 | -2.21048 |
| THC2684001 | 0.0102557 | -2.61698 |
| THC2728054 | 0.0183804 | -2.10482 |
| THC2736258 | 0.0299503 | -2.21114 |
| THC2747565 | 0.0174457 | -2.8976 |
| THC2775681 | 0.000734446 | -2.63446 |
| THC2786127 | 0.00741079 | -1.72088 |
| THNSL1 | 0.00509726 | -1.89658 |
| TLR2 | 0.00723018 | -2.32945 |
| TMCC2 | 0.000169153 | -2.31247 |
| TMEFF2 | 0.00609909 | -2.41783 |
| TMEM119 | 0.0227461 | -2.12196 |
| TMEM130 | 0.000552413 | -3.17916 |
| TMEM132A | 0.000299981 | -2.10802 |
| TMEM133 | 0.0424819 | -2.03965 |
| TMEM151B | 0.00260617 | -4.71054 |
| TMEM158 | 0.000623312 | -2.40613 |
| TMEM174 | 0.0190403 | -2.20745 |
| TMEM200A | 0.0309285 | -1.65222 |
| TMEM217 | 0.00390167 | -3.11403 |
| TMEM35 | 0.000826778 | -5.0515 |
| TMEM37 | 0.00136453 | -3.33096 |
| TMEM51 | 0.0345364 | -1.53516 |
| TMEM88 | 0.00329762 | -2.52876 |
| TMOD4 | 0.034374 | -2.17319 |
| TMSB15A | 0.0399547 | -1.94635 |
| TNC | 0.0314633 | -1.99004 |
| TNFAIP2 | 0.0082581 | -2.36502 |
| TNFAIP6 | 5.34E-05 | -6.49534 |
| TNFRSF10B | 0.00199733 | -1.55243 |
| TNFRSF10C | 0.000274324 | -3.7162 |
| TNFRSF14 | 0.0386091 | -1.66503 |
| TNFRSF19 | 0.00780165 | -1.83976 |
| TNFRSF25 | 0.0152215 | -2.20035 |
| TNFRSF6B | 0.00509015 | -1.59862 |
| TNFSF10 | 0.000274343 | -3.20917 |
| TNFSF13B | 0.00355092 | -3.68827 |
| TNFSF9 | 0.00806837 | -1.90094 |
| TNNC1 | 0.00161172 | -2.5133 |
| TNNC2 | 0.000123972 | -3.324 |
| TNXB | 0.00118586 | -3.08917 |
| TP53INP1 | 0.000647881 | -1.88281 |
| TPPP3 | 0.031881 | -2.51502 |
| TPST1 | 0.00888198 | -1.86148 |
| TRERF1 | 0.00501907 | -1.73666 |
| TRIM2 | 0.00155004 | -1.96642 |
| TRIM21 | 0.0471284 | -1.53713 |
| TRIM45 | 0.00678146 | -4.55955 |
| TRIM47 | 0.00322903 | -1.8807 |
| TRIM5 | 0.0113209 | -1.73993 |
| TRO | 0.00317309 | -1.93016 |
| TRPC3 | 0.0133805 | -1.5637 |
| TRPV5 | 0.0167833 | -1.9719 |
| TSHZ1 | 0.0199357 | -1.8768 |
| TSHZ3 | 0.00902935 | -1.66139 |
| TSPAN11 | 0.00290098 | -2.23185 |
| TSPAN13 | 0.0259657 | -1.83311 |
| TSPO2 | 0.000862874 | -1.5376 |
| TSPYL2 | 0.00191566 | -1.88053 |
| TTC25 | 0.024425 | -2.96863 |
| TUBA4A | 0.000572019 | -1.53531 |
| TUBB3 | 0.00857235 | -1.7158 |
| TULP4 | 0.000689213 | -1.53058 |
| TXLNB | 0.0429225 | -1.84139 |
| UACA | 0.00589047 | -2.21522 |
| UBA7 | 0.0301087 | -1.65149 |
| UBXN11 | 0.0481713 | -1.71949 |
| UCA1 | 0.00786318 | -1.58343 |
| UCN | 0.00585486 | -1.912 |
| ULBP2 | 0.00251685 | -1.51659 |
| ULK1 | 0.00548808 | -1.57209 |
| UNK | 0.00226806 | -1.52174 |
| USP51 | 0.0236053 | -2.76066 |
| USP54 | 0.00228257 | -1.71923 |
| VASH2 | 0.00205828 | -3.13963 |
| VEGFA | 0.0275909 | -1.77203 |
| VEGFB | 0.00301141 | -1.52557 |
| VILL | 0.00155685 | -1.73271 |
| VLDLR | 0.0127673 | -1.50482 |
| VPS37D | 0.000783452 | -2.05663 |
| VWCE | 8.00E-05 | -3.65436 |
| VWF | 0.00915893 | -1.61966 |
| WARS | 0.0128619 | -1.61801 |
| WARS2 | 0.00228704 | -1.55172 |
| WBP1 | 0.00041743 | -1.91202 |
| WDR63 | 5.60E-05 | -2.63463 |
| WNT2 | 0.000369006 | -10.3133 |
| WNT2B | 0.0127353 | -1.78438 |
| WNT3 | 0.00517452 | -1.70115 |
| X58402 | 0.0248239 | -1.64457 |
| XAF1 | 0.000161067 | -2.28884 |
| XRCC6BP1 | 0.002951 | -1.50337 |
| YPEL4 | 0.00432978 | -2.06729 |
| ZBED3 | 0.000511679 | -2.36826 |
| ZBTB2 | 0.00617818 | -1.57653 |
| ZBTB22 | 0.00767246 | -1.52643 |
| ZBTB46 | 0.000292982 | -2.80514 |
| ZC3H6 | 0.00712688 | -1.90681 |
| ZCCHC24 | 0.00157062 | -1.59276 |
| ZCWPW1 | 0.000625022 | -1.69246 |
| ZFP36L1 | 0.0091692 | -1.73015 |
| ZFP90 | 0.000994499 | -1.58216 |
| ZFPM2 | 0.00131544 | -1.62492 |
| ZMYM3 | 0.00955178 | -1.63807 |
| ZNF117 | 0.0434581 | -1.9642 |
| ZNF165 | 0.0311185 | -1.80038 |
| ZNF167 | 0.000136029 | -1.58731 |
| ZNF177 | 0.0322846 | -1.83733 |
| ZNF184 | 0.00647526 | -1.92997 |
| ZNF204P | 0.0128013 | -3.24719 |
| ZNF213 | 0.0229246 | -1.65316 |
| ZNF226 | 0.00277834 | -1.5704 |
| ZNF236 | 0.016074 | -1.69116 |
| ZNF250 | 0.00468629 | -1.56646 |
| ZNF260 | 0.00180438 | -1.65852 |
| ZNF323 | 0.0165939 | -3.03617 |
| ZNF362 | 0.00889096 | -1.60682 |
| ZNF385A | 0.00377859 | -1.75319 |
| ZNF397 | 0.00233333 | -1.65168 |
| ZNF436 | 0.00816743 | -1.60184 |
| ZNF446 | 0.00239013 | -1.60248 |
| ZNF503 | 0.00410143 | -1.79243 |
| ZNF521 | 0.00148623 | -2.04815 |
| ZNF524 | 0.00825171 | -1.58662 |
| ZNF561 | 0.00114743 | -1.58857 |
| ZNF575 | 0.00369331 | -1.6352 |
| ZNF581 | 0.0104837 | -2.00658 |
| ZNF610 | 0.0451624 | -2.15479 |
| ZNF618 | 0.00578116 | -1.83583 |
| ZNF624 | 0.0419483 | -1.81446 |
| ZNF821 | 0.000567875 | -2.79319 |

**Additional Table 5: Baseline levels of miRNAs in healthy ASM.**

| **miRNA** | **Fold-Change** | **SEM** |
| --- | --- | --- |
| ebv-miR-BART13 | 6.44982 | 5.4868 |
| hsa-let-7a | 11.5206 | 10.0534 |
| hsa-let-7b | 10.3816 | 9.26035 |
| hsa-let-7c | 9.52603 | 8.07116 |
| hsa-let-7d | 8.09902 | 6.74566 |
| hsa-let-7e | 7.98888 | 6.67853 |
| hsa-let-7f | 11.0446 | 9.57629 |
| hsa-let-7g | 8.42421 | 7.36438 |
| hsa-let-7i | 9.0506 | 8.05988 |
| hsa-miR-100 | 10.3301 | 8.57039 |
| hsa-miR-101 | 6.18515 | 5.03737 |
| hsa-miR-103 | 8.60477 | 7.44435 |
| hsa-miR-106b | 7.27624 | 6.01185 |
| hsa-miR-107 | 8.34855 | 7.31325 |
| hsa-miR-10a | 7.31582 | 7.42243 |
| hsa-miR-10b | 5.44431 | 5.40083 |
| hsa-miR-1181 | 6.35927 | 6.5428 |
| hsa-miR-1202 | 7.43589 | 7.29248 |
| hsa-miR-1207-5p | 6.77824 | 7.45715 |
| hsa-miR-1208 | 4.90625 | 4.66898 |
| hsa-miR-1224-5p | 4.83203 | 4.70966 |
| hsa-miR-1225-3p | 4.76787 | 4.94543 |
| hsa-miR-1225-5p | 7.39969 | 6.9796 |
| hsa-miR-1226* | 4.68036 | 4.78402 |
| hsa-miR-1228 | 5.30919 | 5.10498 |
| hsa-miR-1234 | 5.76177 | 5.34312 |
| hsa-miR-1238 | 4.90339 | 4.83301 |
| hsa-miR-125a-3p | 5.47357 | 5.02469 |
| hsa-miR-125a-5p | 7.43139 | 6.55771 |
| hsa-miR-125b | 11.7657 | 10.6694 |
| hsa-miR-1260 | 11.775 | 11.5055 |
| hsa-miR-1260b | 11.5536 | 10.4901 |
| hsa-miR-1268 | 5.51134 | 5.27717 |
| hsa-miR-127-3p | 6.49103 | 6.1504 |
| hsa-miR-1274a | 11.5457 | 9.6109 |
| hsa-miR-1274b | 13.5951 | 12.3943 |
| hsa-miR-1275 | 5.25084 | 4.88932 |
| hsa-miR-128 | 5.13697 | 4.6789 |
| hsa-miR-1280 | 8.11756 | 7.61107 |
| hsa-miR-1288 | 4.88816 | 4.59809 |
| hsa-miR-1305 | 5.57741 | 5.05784 |
| hsa-miR-130a | 8.76669 | 7.84068 |
| hsa-miR-130b | 6.22079 | 5.47918 |
| hsa-miR-132 | 4.94636 | 4.87279 |
| hsa-miR-134 | 5.73584 | 5.83827 |
| hsa-miR-135a* | 5.24254 | 4.69557 |
| hsa-miR-136 | 6.45964 | 4.98278 |
| hsa-miR-136* | 5.09399 | 4.63324 |
| hsa-miR-137 | 6.3391 | 5.5862 |
| hsa-miR-138 | 6.64264 | 5.36288 |
| hsa-miR-140-3p | 5.78125 | 5.30064 |
| hsa-miR-140-5p | 6.11243 | 5.39719 |
| hsa-miR-143 | 5.93727 | 5.44854 |
| hsa-miR-145 | 7.39956 | 7.39581 |
| hsa-miR-146b-5p | 5.23249 | 4.73649 |
| hsa-miR-148a | 5.42323 | 5.08851 |
| hsa-miR-148b | 5.48067 | 4.89892 |
| hsa-miR-149 | 5.37864 | 4.858 |
| hsa-miR-150* | 5.71399 | 5.65919 |
| hsa-miR-151-3p | 5.916 | 5.13555 |
| hsa-miR-151-5p | 7.45087 | 6.25901 |
| hsa-miR-152 | 5.89897 | 5.0957 |
| hsa-miR-154 | 5.72244 | 4.80832 |
| hsa-miR-154* | 5.19993 | 4.98354 |
| hsa-miR-155 | 7.59891 | 5.95594 |
| hsa-miR-15a | 7.64006 | 6.39999 |
| hsa-miR-15b | 8.99713 | 7.90069 |
| hsa-miR-16 | 9.73432 | 8.3747 |
| hsa-miR-17 | 6.76942 | 5.58923 |
| hsa-miR-181a | 6.18289 | 5.76225 |
| hsa-miR-181b | 5.39287 | 5.0241 |
| hsa-miR-185 | 5.4235 | 4.82004 |
| hsa-miR-186 | 5.27861 | 4.77686 |
| hsa-miR-188-5p | 4.72168 | 4.77817 |
| hsa-miR-1914* | 5.09491 | 4.73945 |
| hsa-miR-1915 | 8.84066 | 8.06222 |
| hsa-miR-193a-3p | 8.4224 | 5.61365 |
| hsa-miR-193a-5p | 5.38303 | 5.18711 |
| hsa-miR-193b | 7.56989 | 6.94226 |
| hsa-miR-195 | 5.84155 | 5.45926 |
| hsa-miR-197 | 5.6528 | 5.12248 |
| hsa-miR-199a-3p | 10.7074 | 9.47989 |
| hsa-miR-199a-5p | 9.08186 | 7.45145 |
| hsa-miR-199b-5p | 8.85637 | 7.47836 |
| hsa-miR-19a | 6.91434 | 5.50596 |
| hsa-miR-19b | 8.11117 | 6.56554 |
| hsa-miR-20a | 7.38461 | 6.09673 |
| hsa-miR-20b | 6.15979 | 5.07666 |
| hsa-miR-21 | 12.3768 | 10.5414 |
| hsa-miR-21* | 6.13309 | 4.83083 |
| hsa-miR-210 | 5.62401 | 4.70592 |
| hsa-miR-212 | 5.60667 | 4.67619 |
| hsa-miR-214 | 7.0309 | 6.1275 |
| hsa-miR-22 | 9.94654 | 9.55864 |
| hsa-miR-22* | 5.6842 | 4.97574 |
| hsa-miR-221 | 9.69438 | 8.62268 |
| hsa-miR-221* | 5.66159 | 5.09327 |
| hsa-miR-222 | 7.93665 | 6.6994 |
| hsa-miR-23a | 10.9346 | 10.11 |
| hsa-miR-23b | 8.2862 | 8.24347 |
| hsa-miR-24 | 9.86797 | 9.10786 |
| hsa-miR-25 | 6.98125 | 5.93833 |
| hsa-miR-26a | 9.50157 | 8.17846 |
| hsa-miR-26b | 7.80522 | 6.89066 |
| hsa-miR-27a | 9.99156 | 8.48432 |
| hsa-miR-27b | 8.22047 | 7.94605 |
| hsa-miR-28-5p | 5.35636 | 5.00946 |
| hsa-miR-2861 | 5.84153 | 6.79683 |
| hsa-miR-299-5p | 6.16375 | 5.34446 |
| hsa-miR-29a | 11.4309 | 10.1962 |
| hsa-miR-29b | 10.8371 | 8.82748 |
| hsa-miR-29b-1* | 5.24599 | 4.8682 |
| hsa-miR-29c | 9.17171 | 7.60444 |
| hsa-miR-301a | 5.58386 | 4.87948 |
| hsa-miR-30a | 6.80831 | 6.82153 |
| hsa-miR-30a* | 5.12979 | 4.98846 |
| hsa-miR-30b | 9.8011 | 5.74434 |
| hsa-miR-30c | 6.4077 | 5.66451 |
| hsa-miR-30d | 6.44992 | 5.78742 |
| hsa-miR-30e | 6.04107 | 5.20929 |
| hsa-miR-30e* | 5.62085 | 4.69533 |
| hsa-miR-31 | 8.12424 | 6.61763 |
| hsa-miR-31* | 7.07237 | 5.60003 |
| hsa-miR-3125 | 4.91905 | 4.686 |
| hsa-miR-3127 | 4.89871 | 4.55 |
| hsa-miR-3132 | 5.72821 | 4.82103 |
| hsa-miR-3162 | 8.35427 | 7.23087 |
| hsa-miR-3188 | 5.04523 | 5.05659 |
| hsa-miR-3195 | 6.65964 | 6.38752 |
| hsa-miR-3196 | 6.62046 | 6.39915 |
| hsa-miR-3198 | 5.76379 | 5.11789 |
| hsa-miR-320a | 6.75757 | 5.69413 |
| hsa-miR-320b | 7.10446 | 6.10374 |
| hsa-miR-320c | 6.42133 | 5.75117 |
| hsa-miR-320d | 6.71385 | 5.92957 |
| hsa-miR-320e | 6.42645 | 5.75382 |
| hsa-miR-324-3p | 7.2762 | 5.98221 |
| hsa-miR-324-5p | 6.31641 | 5.58739 |
| hsa-miR-331-3p | 6.744 | 6.04189 |
| hsa-miR-337-3p | 5.81667 | 4.85922 |
| hsa-miR-337-5p | 5.79767 | 5.17291 |
| hsa-miR-342-3p | 6.00614 | 5.54105 |
| hsa-miR-34a | 8.94976 | 7.9938 |
| hsa-miR-34b* | 6.28681 | 5.68516 |
| hsa-miR-34c-5p | 5.03042 | 5.00104 |
| hsa-miR-361-5p | 6.54035 | 5.79227 |
| hsa-miR-3646 | 4.68674 | 4.63341 |
| hsa-miR-3648 | 5.42097 | 5.12363 |
| hsa-miR-365 | 9.06315 | 8.17039 |
| hsa-miR-3651 | 6.9068 | 5.64659 |
| hsa-miR-3653 | 5.32522 | 4.80283 |
| hsa-miR-3656 | 5.56079 | 5.6016 |
| hsa-miR-3659 | 5.73907 | 4.83301 |
| hsa-miR-3663-3p | 9.19703 | 6.33014 |
| hsa-miR-3665 | 9.1246 | 9.59628 |
| hsa-miR-3679-5p | 5.57002 | 5.26824 |
| hsa-miR-371-5p | 6.20479 | 5.5863 |
| hsa-miR-374a | 6.6345 | 5.45363 |
| hsa-miR-374b | 5.69459 | 5.1073 |
| hsa-miR-376a | 7.11609 | 6.21582 |
| hsa-miR-376a* | 5.27559 | 4.7091 |
| hsa-miR-376c | 7.1586 | 6.30386 |
| hsa-miR-377 | 6.84109 | 5.87505 |
| hsa-miR-379 | 5.89087 | 5.20699 |
| hsa-miR-381 | 6.022 | 5.18395 |
| hsa-miR-382 | 5.51678 | 4.96781 |
| hsa-miR-3907 | 5.16766 | 4.70865 |
| hsa-miR-409-3p | 5.93499 | 5.52708 |
| hsa-miR-410 | 5.41761 | 5.08486 |
| hsa-miR-423-5p | 5.38129 | 4.80665 |
| hsa-miR-424 | 8.28671 | 7.5054 |
| hsa-miR-425 | 5.48195 | 4.84013 |
| hsa-miR-4257 | 5.02835 | 4.83812 |
| hsa-miR-4261 | 5.4855 | 5.34439 |
| hsa-miR-4270 | 5.072 | 5.30909 |
| hsa-miR-4271 | 5.77293 | 5.60821 |
| hsa-miR-4281 | 10.0206 | 8.43091 |
| hsa-miR-4284 | 10.9568 | 9.30247 |
| hsa-miR-4286 | 11.7492 | 10.7272 |
| hsa-miR-4291 | 6.23626 | 5.21748 |
| hsa-miR-4299 | 6.81155 | 6.32255 |
| hsa-miR-4306 | 5.51597 | 4.95244 |
| hsa-miR-432 | 5.30161 | 4.97326 |
| hsa-miR-4324 | 6.06231 | 5.2973 |
| hsa-miR-4327 | 5.33001 | 5.34783 |
| hsa-miR-450a | 5.76803 | 5.02733 |
| hsa-miR-455-3p | 5.97582 | 5.52035 |
| hsa-miR-484 | 5.50677 | 5.00142 |
| hsa-miR-487b | 6.14873 | 5.38084 |
| hsa-miR-490-3p | 4.70415 | 4.78818 |
| hsa-miR-490-5p | 4.88571 | 4.86534 |
| hsa-miR-493* | 5.93713 | 5.55576 |
| hsa-miR-494 | 7.81044 | 6.92983 |
| hsa-miR-495 | 5.65425 | 5.08896 |
| hsa-miR-497 | 5.41416 | 5.10314 |
| hsa-miR-503 | 4.81379 | 4.89154 |
| hsa-miR-505 | 5.28726 | 4.87905 |
| hsa-miR-542-3p | 5.06178 | 4.79237 |
| hsa-miR-542-5p | 4.84664 | 4.78762 |
| hsa-miR-574-3p | 6.18761 | 5.65366 |
| hsa-miR-574-5p | 6.4126 | 5.40425 |
| hsa-miR-575 | 5.28162 | 4.99129 |
| hsa-miR-590-5p | 5.52101 | 4.72928 |
| hsa-miR-629* | 4.95535 | 5.02899 |
| hsa-miR-630 | 6.47528 | 5.77543 |
| hsa-miR-638 | 5.94952 | 6.58502 |
| hsa-miR-642b | 5.80777 | 5.43522 |
| hsa-miR-654-3p | 6.23317 | 5.49254 |
| hsa-miR-660 | 5.20978 | 4.63579 |
| hsa-miR-663 | 4.83675 | 4.95668 |
| hsa-miR-718 | 4.90086 | 4.63687 |
| hsa-miR-720 | 14.4069 | 14.3275 |
| hsa-miR-758 | 5.26591 | 4.90554 |
| hsa-miR-762 | 7.21309 | 7.00569 |
| hsa-miR-874 | 5.5823 | 5.20362 |
| hsa-miR-92a | 6.47466 | 5.98366 |
| hsa-miR-93 | 6.94965 | 5.84415 |
| hsa-miR-939 | 6.22924 | 5.64588 |
| hsa-miR-940 | 6.19237 | 6.5123 |
| hsa-miR-98 | 6.00866 | 5.05945 |
| hsa-miR-99a | 8.08689 | 6.31472 |
| hsa-miR-99b | 6.08955 | 5.55874 |
| hsv1-miR-H15 | 5.44308 | 4.76597 |
| hsv1-miR-H17 | 4.54397 | 4.73311 |
| hsv1-miR-H18 | 6.26273 | 5.7166 |
| hsv2-miR-H10 | 5.65744 | 6.18373 |
| hsv2-miR-H24 | 5.35061 | 5.42374 |
| hsv2-miR-H25 | 5.69903 | 5.40036 |
| hsv2-miR-H6 | 7.90198 | 6.4048 |
| kshv-miR-K12-3 | 7.36192 | 6.6909 |

**Additional Table 6: lncRNAs increased in expression in healthy ASM cells after stimulation with FCS.**

| **Class of lncRNA** | **Ensembl gene ID** | **Transcript** | **Name** | ***P* value** | **Fold change** |
| --- | --- | --- | --- | --- | --- |
| lincRNA | ENST00000455395 | FTX | FTX transcript, XIST regulator | 0.01564 | 4.36 |
| lincRNA | ENST00000531319 | RP11-627G23.1 |  | 0.01719 | 4.16 |
| Pseudogene | ENST00000452254 | NKAPP1 | NFKB activating protein pseudogene 1 | 0.03719 | 3.78 |
| lincRNA | ENST00000437696 | RP11-359G22.2 |  | 0.04609 | 2.63 |
| lincRNA | ENST00000444958 | DANCR-004 | Differentiation antagonizing non-protein coding | 0.01320 | 2.59 |
| lincRNA | ENST00000431616 | LINC00630-004 | Long intergenic RNA 630 | 0.00022 | 2.43 |
| lincRNA | ENST00000436187 | AC011747.4 |  | 0.00796 | 2.36 |
| lincRNA | ENST00000431411 | AC108868.5 |  | 0.00167 | 2.31 |
| lincRNA | ENST00000424786 | AC018359.1 |  | 0.03482 | 2.28 |
| lincRNA | ENST00000418006 | LINC00940-001 | Long intergenic RNA 940 | 0.01450 | 2.16 |
| lincRNA | ENST00000473636 | LINC00882-002 | Long intergenic RNA 882 | 0.04025 | 2.14 |
| lincRNA | ENST00000507241 | CTC-428G20.3 |  | 0.01732 | 2.09 |
| lincRNA | ENST00000451910 | RP11-344J7.2 |  | 0.00647 | 2.06 |
| lincRNA | ENST00000426635 | LINC00472 | Long intergenic RNA 472 | 0.04611 | 1.86 |
| lincRNA | ENST00000495228 | LINC00883-005 | Long intergenic RNA 883 | 0.03381 | 1.63 |
| lincRNA | ENST00000515871 | CTC-325J23.3 |  | 0.01419 | 1.60 |
| lincRNA | ENST00000523328 | PVT1 | Pvt1 oncogene | 0.00575 | 1.39 |

**Additional Table 7: lncRNAs decreased in expression in healthy ASM cells after stimulation with FCS.**

| **Class of lncRNA** | **Ensembl gene ID** | **Transcript** | **Name** | ***P* value** | **Fold change** |
| --- | --- | --- | --- | --- | --- |
| lincRNA | ENST00000418539 | BCYRN1 | Brain cytoplasmic RNA 1 | 0.04526 | -1.53 |
| lincRNA | ENST00000544717 | RP11-319E16.1 |  | 0.00186 | -1.54 |
| lincRNA | ENST00000505448 | RP11-774O3.3 |  | 0.00083 | -1.57 |
| Antisence | ENST00000420095 | LMCD1-AS1-001 |  | 0.01766 | -1.57 |
| lincRNA | ENST00000559211 | CTD-2034I4.1 |  | 0.04697 | -1.59 |
| lincRNA | ENST00000442316 | AC074363.1 |  | 0.01147 | -1.60 |
| lincRNA | ENST00000521456 | RP11-379I19.1 |  | 0.00083 | -1.60 |
| Antisence | ENST00000415647 | RP11-46A10.4 |  | 0.01087 | -1.62 |
| Antisence | ENST00000507963 | NR2F1-AS1-003 |  | 0.01525 | -1.66 |
| lincRNA | ENST00000464767 | LINC00341 | Long intergenic RNA 341 | 0.00454 | -1.69 |
| lincRNA | ENST00000518765 | RP11-527N22.1 |  | 0.04078 | -1.69 |
| lincRNA | ENST00000307533 | AC093323.3 |  | 0.03067 | -1.72 |
| Known sense overlapping | ENST00000314957 | CTD-2201E18.3 |  | 0.00674 | -1.73 |
| lincRNA | ENST00000433843 | SNHG5 | Small nucleolar RNA host gene 5 | 0.04458 | -1.74 |
| lincRNA | ENST00000444232 | AC093323.3 |  | 0.03067 | -1.75 |
| lincRNA | ENST00000523242 | CTB-43E15.1 |  | 0.04998 | -1.79 |
| lincRNA | ENST00000366185 | RP11-258C19.5 |  | 0.00314 | -1.79 |
| lincRNA | ENST00000414120 | LINC00887-001 | Long intergenic RNA 887 | 0.01041 | -1.81 |
| Known processed transcript | ENST00000446423 | SDCBP2-AS1-001 | SDCBP2 antisense RNA 1 | 0.02776 | -1.82 |
| lincRNA | ENST00000428276 | RP11-175B12.2 |  | 0.00001 | -1.83 |
| lincRNA | ENST00000456895 | AC068196.1 |  | 0.04126 | -1.83 |
| lincRNA | ENST00000515296 | CTB-35F21.1 |  | 0.04701 | -1.83 |
| Antisence | ENST00000496733 | TMEM161B-AS1-010 |  | 0.02974 | -1.90 |
| lincRNA | ENST00000431759 | SLC2A1-AS1-002 |  | 0.04943 | -1.95 |
| lincRNA | ENST00000520606 | RP11-10J21.5 |  | 0.03944 | -2.00 |
| lincRNA | ENST00000501652 | CTC-228N24.3 |  | 0.01010 | -2.02 |
| lincRNA | ENST00000522667 | RP11-419K12.1 |  | 0.04072 | -2.03 |
| lincRNA | ENST00000521128 | CTB-43E15.3 |  | 0.04998 | -2.07 |
| lincRNA | ENST00000448786 | AC007879.2 |  | 0.04139 | -2.19 |
| lincRNA | ENST00000515306 | CTB-35F21.2 |  | 0.02244 | -2.23 |
| lincRNA | ENST00000421002 | LINC00423 | Long intergenic RNA 423 | 0.02096 | -2.26 |
| lincRNA | ENST00000417485 | AC012668.2 |  | 0.00554 | -2.28 |
| Antisence | ENST00000507736 | TMEM161B-AS1-011 |  | 0.02974 | -2.33 |
| lincRNA | ENST00000499418 | RP11-383J24.5 |  | 0.02958 | -2.47 |
| lincRNA | ENST00000450531 | AC079776.1 |  | 0.02064 | -2.48 |
| Antisence | ENST00000509179 | MEF2C-AS1-002 |  | 0.02762 | -2.59 |
| lincRNA | ENST00000544034 | RP11-662M24.2 |  | 0.00488 | -2.73 |
| Known processed transcript | ENST00000453698 | SNHG11 | Small nucleolar RNA host gene 11 | 0.00113 | -2.85 |
| lincRNA | ENST00000444488 | TPRG1-AS1 | TPRG1 antisense RNA 1 | 0.00274 | -4.00 |
| lincRNA | ENST00000412788 | H19 | H19, imprinted maternally expressed transcript | 0.02451 | -6.60 |

**Additional Table 8: lncRNAs increased in expression in healthy ASM cells after stimulation with Dex + FCS.**

| **Class of lncRNA** | **Ensembl gene ID** | **Transcript** | **Name** | ***P* value** | **Fold change** |
| --- | --- | --- | --- | --- | --- |
| Antisence | ENST00000420095 | LMCD1-AS1-001 |  | 0.02043 | 5.65 |
| lincRNA | ENST00000442796 | LINC00312 | Long intergenic RNA 312 | 0.02043 | 3.97 |
| lincRNA | ENST00000444229 | RP11-527F13.1 |  | 0.00469 | 2.79 |
| lincRNA | ENST00000426635 | LINC00472 | Long intergenic RNA 472 | 0.02601 | 2.64 |
| lincRNA | ENST00000444958 | DANCR-004 | Differentiation antagonizing non-protein coding | 0.00305 | 2.38 |
| Known processed transcript | ENST00000456532 | RP5-1158E12.3 |  | 0.01899 | 2.38 |
| lincRNA | ENST00000418539 | BCYRN1 | Brain cytoplasmic RNA 1 | 0.04436 | 2.37 |
| Antisence | ENST00000425604 | ZNRD1-AS1 | ZNRD1 antisense RNA 1 | 0.01785 | 2.36 |
| lincRNA | ENST00000505254 | MIR143HG | MIR143 host gene | 0.01580 | 2.36 |
| lincRNA | ENST00000505155 | RP11-584P21.2 |  | 0.00048 | 2.34 |
| lincRNA | ENST00000512322 | RP11-792D21.2 |  | 0.00123 | 2.26 |
| lincRNA | ENST00000473636 | LINC00882-002 | Long intergenic RNA 882 | 0.04628 | 2.08 |
| lincRNA | ENST00000454185 | RP11-568A7.3 |  | 0.00320 | 2.06 |
| lincRNA | ENST00000521586 | RP11-382A18.2 |  | 0.01054 | 2.01 |
| lincRNA | ENST00000417947 | AC096574.5 |  | 0.00037 | 1.96 |
| lincRNA | ENST00000432894 | AC062020.1 |  | 0.02085 | 1.81 |
| Known sense overlapping | ENST00000314957 | CTD-2201E18.3 |  | 0.03776 | 1.78 |
| lincRNA | ENST00000440888 | RP11-315I14.2 |  | 0.02126 | 1.78 |
| lincRNA | ENST00000504245 | SRP14-AS1-001 | SRP14 antisense RNA1 (head to head) | 0.03739 | 1.78 |
| lincRNA | ENST00000495228 | LINC00883-005 | Long intergenic RNA 883 | 0.01735 | 1.78 |
| lincRNA | ENST00000414407 | LINC00398 | Long intergenic RNA 398 | 0.02941 | 1.72 |
| lincRNA | ENST00000515871 | CTC-325J23.3 |  | 0.00774 | 1.72 |
| lincRNA | ENST00000471357 | LINC00880-001 | Long intergenic RNA 880 | 0.00188 | 1.72 |
| lincRNA | ENST00000523328 | PVT1 | Pvt1 oncogene | 0.00066 | 1.66 |
| lincRNA | ENST00000451910 | RP11-344J7.2 |  | 0.02863 | 1.66 |
| lincRNA | ENST00000515769 | RP11-93L9.1 |  | 0.02316 | 1.63 |
| lincRNA | ENST00000529893 | RP1-80B9.2 |  | 0.03654 | 1.61 |

**Additional Table 9: Changes in expression of lncRNAs in healthy ASM cells after stimulation with Dex + FCS.**

| **Class of lncRNA** | **Ensembl gene ID** | **Transcript** | **Name** | ***P* value** | **Fold change** |
| --- | --- | --- | --- | --- | --- |
| lincRNA | ENST00000452028 | AF131217.1 |  | 0.04395 | -1.50 |
| lincRNA | ENST00000433843 | SNHG5 | Small nucleolar RNA host gene 5 | 0.00331 | -1.51 |
| lincRNA | ENST00000518765 | RP11-527N22.1 |  | 0.01786 | -1.53 |
| lincRNA | ENST00000444265 | LINC00340 | Long intergenic RNA 340 | 0.00551 | -1.55 |
| Known processed transcript | ENST00000430050 | RP11-134D3.1-001 |  | 0.02415 | -1.59 |
| lincRNA | ENST00000423943 | RP11-48O20.4 |  | 0.00585 | -1.60 |
| Antisence | ENST00000507963 | NR2F1-AS1-003 |  | 0.02035 | -1.61 |
| lincRNA | ENST00000442316 | AC074363.1 |  | 0.01033 | -1.62 |
| lincRNA | ENST00000444125 | RP11-65J3.1 |  | 0.03164 | -1.62 |
| lincRNA | ENST00000454635 | LINC00963-006 | Long intergenic RNA 963 | 0.00064 | -1.64 |
| lincRNA | ENST00000435043 | RP1-40E16.9 |  | 0.00361 | -1.64 |
| Antisence | ENST00000415647 | RP11-46A10.4 |  | 0.02185 | -1.65 |
| lincRNA | ENST00000437615 | LINC00242 | Long intergenic RNA 242 | 0.00120 | -1.65 |
| lincRNA | ENST00000420557 | LINC00574 | Long intergenic RNA 574 | 0.00120 | -1.65 |
| lincRNA | ENST00000505448 | RP11-774O3.3 |  | 0.00111 | -1.66 |
| Antisence | ENST00000434399 | AC005154.6 |  | 0.04284 | -1.70 |
| Known retained intron | ENST00000426529 | AC005154.6 |  | 0.04284 | -1.71 |
| Putative processed transcript | ENST00000504610 | RP11-356J5.12 |  | 0.04579 | -1.71 |
| lincRNA | ENST00000552334 | RP11-701H24.2 |  | 0.00868 | -1.71 |
| lincRNA | ENST00000443548 | RP11-435B5.4-003 |  | 0.01194 | -1.77 |
| lincRNA | ENST00000417485 | AC012668.2 |  | 0.00867 | -1.81 |
| Known processed transcript | ENST00000446423 | SDCBP2-AS1-001 | SDCBP2 antisense RNA 1 | 0.02817 | -1.82 |
| lincRNA | ENST00000430244 | RP11-166O4.5 |  | 0.00253 | -1.86 |
| lincRNA | ENST00000553704 | LINC-ROR-001 | Long intergenic non-protein coding RNA, regulator of reprogramming | 0.00463 | -1.93 |
| lincRNA | ENST00000414120 | LINC00887-001 | Long intergenic RNA 887 | 0.00634 | -1.93 |
| lincRNA | ENST00000515296 | CTB-35F21.1 |  | 0.03429 | -1.94 |
| lincRNA | ENST00000523242 | CTB-43E15.1 |  | 0.00112 | -2.02 |
| lincRNA | ENST00000454596 | RP11-69I8.2 |  | 0.00097 | -2.06 |
| lincRNA | ENST00000521128 | CTB-43E15.3 |  | 0.00112 | -2.06 |
| lincRNA | ENST00000431759 | SLC2A1-AS1-002 |  | 0.03541 | -2.09 |
| lincRNA | ENST00000513051 | CT49-002 | Cancer/testis antigen 49 (non-protein coding) | 0.03145 | -2.14 |
| Antisence | ENST00000456602 | RBM26-AS1 | RBM26 antisense RNA 1 | 0.01598 | -2.14 |
| lincRNA | ENST00000515306 | CTB-35F21.2 |  | 0.02686 | -2.15 |
| Antisence | ENST00000509179 | MEF2C-AS1-002 | MEF2C antisense RNA 1 | 0.00323 | -2.16 |
| Known processed transcript | ENST00000453698 | SNHG11 | Small nucleolar RNA host gene 11 | 0.00419 | -2.24 |
| lincRNA | ENST00000420187 | AC007317.1 |  | 0.00157 | -2.33 |
| lincRNA | ENST00000470712 | LINC00877-003 | Long intergenic RNA 877 | 0.04265 | -2.41 |
| lincRNA | ENST00000440438 | AC005154.9 |  | 0.04284 | -2.49 |
| Known processed transcript | ENST00000451141 | MIAT-014 | Myocardial infarction associated transcript | 0.04467 | -3.94 |
